# Alkoxy and Enediyne Derivatives Containing 1,4-Benzoquinone Subunits—Synthesis and Antitumor Activity

**DOI:** 10.3390/molecules22030447

**Published:** 2017-03-11

**Authors:** Monika Kadela-Tomanek, Ewa Bębenek, Elwira Chrobak, Małgorzata Latocha, Stanisław Boryczka

**Affiliations:** 1 Department of Organic Chemistry, School of Pharmacy with the Division of Laboratory Medicine in Sosnowiec, Medical University of Silesia in Katowice, 4 Jagiellońska Str., 41-200 Sosnowiec, Poland; ebebenek@sum.edu.pl (E.B.); echrobak@sum.edu.pl (E.C.); boryczka@sum.edu.pl (S.B.); 2 Department of Cell Biology, School of Pharmacy with the Division of Laboratory Medicine in Sosnowiec, Medical University of Silesia in Katowice, 8 Jedności Str., 41-200 Sosnowiec, Poland; mlatocha@sum.edu.pl

**Keywords:** alkoxy substituent, 1,4-benzoquinone, enediyne, antiproliferative activity, Lipinski Rule of Five, blood–brain barrier

## Abstract

The compounds produced by a living organism are most commonly as medicinal agents and starting materials for the preparation of new semi-synthetic derivatives. One of the largest groups of natural compounds consists of products containing a 1,4-benzoquinone subunit. This fragment occurs in three enediyne antibiotics, dynemicin A, deoxydynemicin A, and uncilamicin, which exhibit high biological activity. A series of alkoxy derivatives containing 1,4-naphthoquinone, 5,8-quinolinedione, and 2-methyl-5,8-quinolinedione moieties was synthesized. Moreover, the 1,4-benzoquinone subunit was contacted with an enediyne fragment. All obtained compounds were characterized by spectroscopy and spectrometry methods. The resulting alkane, alkene, alkyne and enediyne derivatives were tested as antitumor agents. They showed high cytotoxic activity depending on the type of 1,4-benzoquinone subunit and the employed tumor cell lines. The synthesized derivatives fulfill the Lipinski Rule of Five and have low permeability through the blood–brain barrier.

## 1. Introduction

Nature is a main source of novel compounds useful as medicinal agents and starting materials for the preparation of semi-synthetic derivatives [1,2,3,4,5,6,7]. The isolated products exhibit a broad spectrum of pharmacological effects, including antioxidant, anticancer, antibacterial, antiviral, and anti-inflammatory activities [2,5,6,7]. The derivatives containing a 1,4-benzoquinone subunit constitute one of the largest and diverse groups of secondary metabolites characterized by a broad range of biological properties [8,9]. The mechanism of their action has not been completely explained. However, a 1,4-benzoquinone unit plays an important role in oxidation processes. It should be noted that this moiety catalyzes oxidation of the intra- and extra-mitochondrial nicotinamide adenine dinucleotide phosphate (NAD(P)H), and the by-products of this process are reactive oxygen species that induce cell death [1,10,11]. The group of natural compounds that contains 1,4-benzoquinone also includes geldanamycin, mitomycin, bolinaquinone, and ubiquinone. Often, this subunit is connected with other fragments creating 1,4-naphthoquinone, anthracycline, and 5,8-quinolinedione, which occur in many chemotherapeutic drugs, such as plubmagin, anthracyclines, and streptonigrin. Unfortunately, the high toxicity of the antibiotics limits their use in the treatment of cancer [12,13,14,15,16].

**Figure 1:**
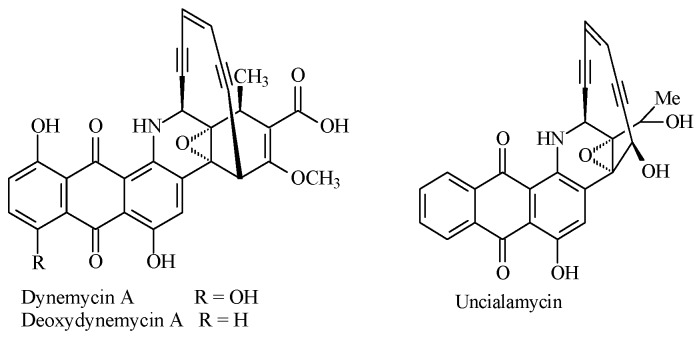
The structure of enediyne antibiotics.

A second interesting group of natural products have been isolated from bacteria enediyne antibiotics [3,5]. These natural products contain a unique nine- or ten-membered unsaturated ring composed of two triple bonds connected through a double bond. The compounds are characterized by an unusual mechanism of action. In the Bergman cyclization, the enediyne ring was converted to a 1,4-diradical, which interacts with DNA and causes breaks in single- and double-stranded molecules [3,5,17]. So far, eleven compounds belonging to the enediyne family have been discovered. Three of them, dynemicin A, deoxydynemicin A, and uncilamicin, contain a 1,4-benzoquinone moiety (Figure 1). These compounds affect the DNA cleavage on two difference routes, directly by reduction of the anthraquinone ring and opening of the epoxide ring and indirectly by the formation of a highly reactive 1,4-diradical intermediate from the (*Z*)-enediyne group [18,19,20]. Thereby, natural conjugates of the enediyne ring and the 1,4-benzoquinone subunit show high activity against Gram-positive and Gram-negative bacteria and antitumor activity against a broad spectrum of human and murine cancer cell lines [2,3,17,21,22,23]. Unlike the other enediyne antibiotics, dynemicin A exhibits low toxicity [22,23].

Continuing our research on synthesis and anticancer activity of alkoxy derivatives of the 5,8-quinolinedione moiety [24,25], alkoxy compounds containing 1,4-naphthoquinone and 2-methyl-5,8-quinolinedione subunit were received. Moreover, the connection of the 1,4-benzoquinone fragment with a 1,5-diyne-3-ene unit allows for the obtainment of enediyne products containing 1,4-naphthoquinone, 5,8-quinolinedione, and 2-methylo-5,8-quinolinedione moieties. For all synthesized derivatives, the antitumor activity against three human cancer cell lines was determined and the pharmacological parameters were calculated.

## 2. Results and Discussion

### 2.1. Chemistry

**Scheme 1:**
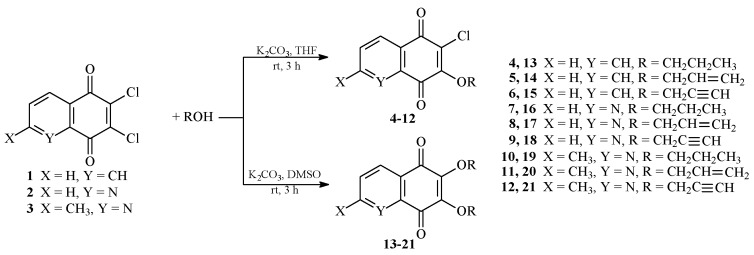
The synthesis of 7-mono and 6,7-disubstituted derivatives (**4**–**21**) containing 1,4-benzoquinone moiety.

The synthesis of alkoxy derivatives **4**–**21** originated from 2,3-dichloro-1,4-naphthoquinone (**1**) or 6,7-dichloro-5,8-quinolinedione (**2**) or 2-methyl-6,7-dichloro-5,8-quinolinedione (**3**). The 7-mono and 6,7-dialkoxy compounds were prepared according to our previously reported methods [24,25]. The synthetic procedures for the obtained compounds are summarized in Scheme 1.

All compounds were purified by flash chromatography. The 7-alkoxy derivatives **2**–**12** and 6,7-dialkoxy compounds **13**–**21** were obtained with 53–76% and 41–72% yields, respectively. The structures of the synthesized derivatives **4**–**21** were confirmed on the basis of ^1^H- and ^13^C-NMR, IR, and HR-MS spectra.

Extending our work in the synthesis of alkoxy derivatives of 1,4-naphthoquinone, 2-methyl-5,8-quinolinedione, and 5,8-quinolinedione, we tried to synthesize enediyne compounds.

(*Z*)-4-octen-2,6-diyn-1,8-diol (**22**) was obtained in the Sonogashira reaction between *cis*-1,2-dichloroethylene and propargyl alcohol. Its chemical structure was confirmed by ^1^H- and ^13^C-NMR, and IR spectra. The spectral data were consistent with those published in the literature [26,27].

**Scheme 2:**
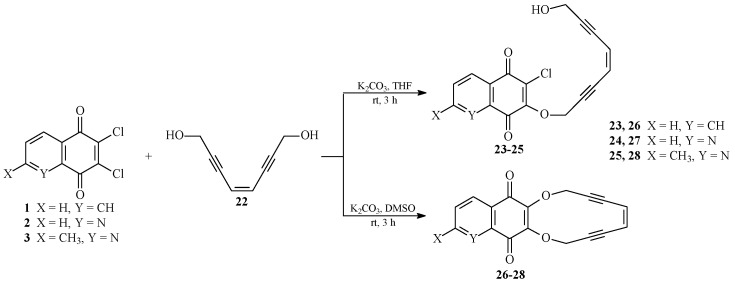
Synthesis of acyclic (**23**–**25**) and cyclic (**26**–**28**) derivatives containing the 1,4-benzoquinone subunit.

A condensation of the 1,4-benzoquinones **1**–**3** with Compound **22** in the presence of potassium carbonate and tetrahydrofuran led to the acyclic enediyne derivatives **23**–**25**. On the other hand, the change of solvent from tetrahydrofuran to dimethyl sulfoxide allowed for the obtainment of the cyclic compounds **26**–**28** (Scheme 2).

The major products of the reactions were separated by flash chromatography to yield the pure derivatives **23**–**28** in 35–51% yields. Chemical structures of the enediyne compounds **23**–**25** were elucidated with the aid of ^1^H-, ^13^C-NMR, IR spectroscopy, and HR-MS analysis.

### 2.2. Antiproliferative Activity

The obtained compounds **1**–**28** were evaluated for cytostatic activity against three human cancer cell lines: melanoma (C-32), glioblastoma (SNB-19), breast cancer (MDA-MB-231), and normal fibroblasts (HFF-1) cell lines. The results are presented in Table 1 and Table 2. Moreover, the selectivity index (SI), expressed as the ratio of IC50 of a normal fibroblast to IC50 of the corresponding cancer cell line, was calculated (Table S1; Supplementary Materials).

The compounds **4**–**6** and **13**–**15**, containing the 1,4-naphthoquinone subunit, had IC_50_ values in the range of 3.2–226.1 µM. Moreover, the introduction of an alkoxy group led to an increase in the antitumor activity against all cancer cell lines compared with 2,3-dichloro-1,4-naphthoquinone (**3**). The 2-alkoxy compounds **4**–**6** exhibited activity that was better than the 2,3-dialkoxy derivatives **13**–**15**. The antiproliferative activity of monoalkoxy derivatives of 1,4-naphthoquinone (**4**–**6**) depends on the type of the bond in the substituent the rank order of activity against the C-32 cancer cell line is as follows: propargyloxy > 2-propenoxy > propoxy. In the series of 2,3-dialkoxy compounds (**13**–**15**), the highest activity against the melanoma cell line (C-32) is exhibited by 2,3-di(2-propenoxy)-1,4-naphtoqinone (**14**).

The second group of the synthesized compounds consisted of the 5,8-quinolinedione derivatives **7**–**12** and **16**–**21**. Comparing the activity of the compounds containing 5,8-quinolinedione (**7**–**9** and **16**–**18**) and 2-methyl-5,8-quinolinedione (**10**–**12** and **19**–**21**), it was shown that the introduction of a methyl group at C-2 position of 5,8-quinolinedione unit led to a decrease in the antiproliferative activity against all tested cancer cell lines. The general trend of the antitumor activity showed that the introduction of alkoxy substituent to the 2-methyl-5,8-quinolinedione moiety led to a reduction of cytotoxicity against all cancer cell lines compared with 6,7-dichloro-2-methyl-5,8-quinolinedione (**2**). One could notice that the derivatives of 2-methyl-5,8-quinolinedione were characterized by a lower selectivity of action (Table S1; Supplementary Materials).

The analysis of the structure–activity relationship showed that the activity of alkoxy derivatives containing the 1,4-benzoquinone subunit depended on the nitrogen atom at the C-1 position and the group at the C-2 position.

The synthesized conjugates of enediyne with the 1,4-benzoquinone subunit exhibited a low antitumor activity against all tested cancer cell lines (Table 2). On the other hand, the obtained compounds showed a higher activity against all cancer cell lines than (*Z*)-4-octen-2,6-diyn-1,8-diol (**22**).

Among the acyclic enediyne compounds **23**–**25**, 6-chloro-7-(8-hydroxy-4-octen-2,6-diynyloxy)-5,8-quinolinedione (**24**) showed good activity against the melanoma (C-32) and glioblastoma (SNB-19) cancer cell lines. This compound exhibited 51-times and 5-times more activity against the C-32 cell compared to (*Z*)-4-octen-2,6-diyn-1,8-diol (**22**) and cisplatin, respectively. The analysis of the structure–activity relationship shows that the antiproliferative activity depends on the 1,4-benzoquinone subunit, the rank order of activity against C-32, and the SNB-19 cancer cells is as follows: 5,8-quinolinedione > 2-methyl-5,8-quinolinedione > 1,4-naphthoquinone.

The cyclic compounds containing 1,4-naphthoquinone (**26**) and 5,8-quinolinedione (**27**) exhibited activity against all tested cancer cell lines. However, only 8-octen-6,10-diynyl-1,4-dioxocyclododeca[2,3-*g*]-1,4-naphtoquinon (**27**) was characterized by a higher activity against the MDA-MB-231 cell than cisplatin.

Physicochemical parameters such as lipophilicity (clog P), molecular mass (M), topological polar surface area (PSA), the number of donors (nHD), the acceptors (nHA) of the hydrogen bond, and the number of rotatable bonds (nRTB) were used to assess absorption, distribution, metabolism, and excretion of the compound in a biological system [28,29,30]. An important property of the newly synthesized compounds is their penetration through the blood–brain barrier (BBB). Determination of log BB (drug penetration through the BBB) allows for predicting their neurotoxicity. All parameters were calculated using ACD/iLab software, and the values are presented in Table S2 (Supplementary Materials).

The obtained compounds **1**–**28** fulfill the Lipinski rules. The ACD/iLab software calculated the number of donors (nHD) of the hydrogen bond depending on the amounts of hydroxyl and amine groups in a compound. One might notice that the nHD number could be higher, because in the derivatives containing the 5,8-quinolinedione subunit, a weak hydrogen bond between the C–H group as a donor and the oxygen atom as an acceptor of the hydrogen bond was observed [24,25,31,32]. The topological polar surface area (PSA) obtained for the discussed compounds is in the range of 34.14–76.50 Å^2^, which determined a high oral bioavailability [33]. According to the literature [34,35], if the log BB value is higher than 0.3, the compounds pass the BBB rapidly; however, a value lower than −1 indicates poor distribution in the blood–brain barrier. The log BB values for the studied compounds were lower than 0.3, suggesting that they could have a low permeability through the blood–brain barrier. Moreover, three of them, **15**, **18**, and **21**, had a log BB value lower than −1, so they had no access to the central nervous system.

## 3. Materials and Methods

### 3.1. Chemistry

Melting points were established on an Electrothermal IA 9300 melting point apparatus. The ^1^H- and ^13^C-NMR spectra were determined using a Bruker Avance 600 spectrometer (Bruker, Billerica, MA, USA) in CDCl_3_; chemical shifts (δ) are reported in ppm and *J* values in Hz. The peak multiplicity is designated as singlet (s), doublet (d), triplet (t), doublet of doublets (dd), doublet of triplets (dt), and multiplet (m). High-resolution mass spectral analysis was measured on a Bruker Impact II instrument (Bruker). Infrared spectra were recorded on an IRAffinity-1 Shimadzu spectrophotometer (Shimadzu Corporation, Kyoto, Japan). Thin layer chromatography (TLC) was performed on silica gel 60 254F plates (Merck, Darmstadt, Germany) using a mixture of chloroform and ethanol (40:1 or 15:1, v/v) as an eluent. The spots were visualized by UV light (254 nm) and iodine. All new compounds were purified by flash chromatography (Reveleris Flash system, Grace technologies, Ellicott City, MD, USA). The column for flash chromatography was filled silica gel (Grace Technologies, Ellicott City, MD, USA). As a mobile phase was used chloroform–ethanol (40:1 or 15:1, v/v). It was isocratically eluted at a flow rate of 2.0 mL/min. The effluent was monitored by UV detector (254 nm) and peak fractions were collected according to the elution profile.

2,3-Dichloro-1,4-naphtoquinon (**1**) was from Sigma–Aldrich. 6,7-dichloro-5,8-quinolinedione (**2**) and 6,7-dichloro-2-methyl-5,8-quinolinedione (**3**) were obtained according to methods described previously [36]. (*Z*)-oct-4-ene-2,6-diyne-1,8-diol (**22**) was prepared according to the literature method [26,27].

#### 3.1.1. General Procedure for the Synthesis of Monoalkoxy Derivatives **4**–**12**

A mixture of 1,4-benzoquinone (**1**–**3**) (0.441 mmol), potassium carbonate (0.061 g, 0.441 mmol), and alcohol (1.2 eqv., 0.529 mmol) in dry tetrahydrofuran (1 mL) was stirred at room temperature for 3 h. The tetrahydrofuran was evaporated under reduced pressure. The residue was purified by silica gel chromatography (chloroform/ethanol, 40:1, v/v) to yield pure products **4**–**12**.

*2-Chloro-3-propoxy-1,4-naphtoquinon* (**4**): 84 mg (0.335 mmol), yield: 76%, m.p. 125–126 °C. The spectra data conformed to previously published literature [37].

*2-Chloro-3-(2-propenoxy)-1,4-naphthoquinon* (**5**): 86 mg (0.34 mmol), yield: 78%, m.p. 74–75 °C. ^1^H-NMR (CDCl_3_, 600 MHz) δ (in ppm): 5.12 (t, *J* = 1.2 Hz, *J* = 6.0 Hz, 2H, OCH_2_), 5.34 (dt, *J* = 1.2 Hz, *J* = 16.2 Hz, 1H, CH=CH_2_), 5.49 (dt, *J* = 1.2 Hz, *J* = 16.2 Hz, 1H, CH=CH_2_), 6.05–6.12 (m, 1H, CH=CH_2_), 7.76–7.78 (m, 2H, H-6, H-7), 8.11 (dd, *J*_57_ = 2.4 Hz, *J*_56_ = 8.4 Hz, 1H, H-5), 8.16 (dd, *J*_78_ = 2.4 Hz, *J*_68_ = 8.4 Hz, 1H, H-8). ^13^C-NMR (CDCl_3_, 150 MHz) δ (in ppm): 73.7 (OCH_2_), 118.5 (CH=CH_2_), 125.9 (C-8), 126.0 (C-5), 128.9 (C-3), 129.8 (C-9), 130.1 (C-10), 131.6 (CH=CH_2_), 132.9 (C-6), 133.3 (C-7), 155.2 (C-2), 177.6 (C-1), 178.8 (C-4). IR (KBr, cm^−1^) ν_max_: 3080–2886, 1680, 1595–1560, 1042. HR-MS (APCI) *m/z*: C_13_H_9_ClO_3_ [M + H]^+^, Calcd.: 249.0318; Found: 249.0311 (Figure S1).

*2-Chloro-3-(2-propynoxy)-1,4-naphthoquinon* (**6**): 71 mg (0.29 mmol), yield: 65%, m.p. 113–114 °C. ^1^H-NMR (CDCl_3_, 600 MHz) δ (in ppm): 2.60 (t, *J* = 2.4 Hz, 1H, CH), 5.31 (d, *J* = 2.4 Hz, 2H, OCH_2_), 7.78–7.80 (m, 2H, H-6, H-7), 8.13 (dd, *J*_57_ = 2.4 Hz, *J*_56_ = 8.4 Hz, 1H, H-5), 8.19 (dd, *J*_78_ = 2.4 Hz, *J*_68_ = 8.4 Hz, 1H, H-8). ^13^C-NMR (CDCl_3_, 150 MHz) δ (in ppm): 61.1 (OCH_2_), 77.4 (C=CH), 77.6 (C=CH), 127.0 (C-8), 127.2 (C-5), 130.7 (C-3), 131.1 (C-9), 131.4 (C-10), 134.1 (C-6), 134.4 (C-7), 154.9 (C-2), 178.4 (C-1), 179.7 (C-4). IR (KBr, cm^−1^) ν_max_: 3266, 2129, 1678, 1588‑1559, 1046. HR-MS (APCI) *m/z*: C_13_H_7_ClO_3_ [M + H]^+^, Calcd.: 247.0162; Found: 247.0164 (Figure S2).

*6-Chloro-2-methyl-7-propoxy-5,8-quinolinedione* (**10**): 58 mg (0.22 mmol), yield: 53%, m.p. 90–91 °C. ^1^H-NMR (CDCl_3_, 600 MHz) δ (in ppm): 1.07 (t, *J* = 7.2 Hz, 3H, CH_3_), 1.82-1.89 (m, 2H, CH_2_CH_3_), 2.78 (s, 3H, CH_3_), 4.61 (t, *J* = 6.6 Hz, OCH_2_), 7.56 (d, *J*_34_ = 7.8 Hz, 1H, H-3), 8.36 (d, *J*_34_ = 7.8 Hz, 1H, H-4). ^13^C-NMR (CDCl_3_, 150 MHz) δ (in ppm): 10.2 (CH_3_), 23.8 (CH_2_CH_3_), 25.2 (CH_3_), 77.5 (OCH_2_), 126.0 (C-4a), 127.9 (C-3), 128.0 (C-6), 135.0 (C-4), 146.2 (C-8a), 157.2 (C-7), 165.1 (C-2), 177.9 (C-8), 178.4 (C-5). IR (KBr, cm^−1^) ν_max_: 2968–2859, 1700, 1666, 1601–1558, 1077. HR-MS (APCI) *m/z*: C_13_H_12_ClNO_3_ [M + H]^+^, Calcd.: 266.0584; Found: 266.0591 (Figure S3).

*6-Chloro-2-methyl-7-(2-propenoxy)-5,8-quinolinedione* (**11**): 71 mg (0.27 mmol), yield: 65%, m.p. 86–87 °C. ^1^H-NMR (CDCl_3_, 600 MHz) δ (in ppm): 2.80 (s, 3H, CH_3_), 5.17 (dt, *J* = 1.2 Hz, *J* = 6.0 Hz, 2H, OCH_2_), 5.33 (dt, *J* = 1.2 Hz, *J* = 9.0 Hz, 1H, CH=CH_2_), 5.48 (dt, *J* = 1.2 Hz, *J* = 16.2 Hz, 1H, CH=CH_2_), 6.06–6.09 (m, 1H, CH=CH_2_), 7.56 (d, *J*_34_ = 7.8 Hz, 1H, H-3), 8.37 (d, *J*_34_ = 7.8 Hz, 1H, H-4). ^13^C-NMR (CDCl_3_, 150 MHz) δ (in ppm): 25.2 (CH_3_), 74.9 (OCH_2_), 119.9 (CH=CH_2_), 126.0 (C-4a), 127.9 (C-3), 129.0 (C-6), 132.5 (CH=CH_2_), 135.0 (C-4), 146.1 (C-8a), 156.6 (C-7), 165.2 (C-2), 177.7 (C-8), 178.3 (C-5). IR (KBr, cm^−1^) ν_max_: 3084–2926, 1690, 1667, 1598–1558, 1073. HR-MS (APCI) *m/z*: C_13_H_10_ClNO_3_ [M + H]^+^, Calcd.: 264.0427; Found: 264.0422 (Figure S4).

*6-Chloro-2-methyl-7-(2-propynoxy)-5,8-quinolinedione* (**12**): 74 mg (0.28 mmol), yield: 68%, m.p. 93–94 °C. ^1^H-NMR (CDCl_3_, 600 MHz) δ (in ppm): 2.59 (t, *J* = 2.4 Hz, 1H, CH), 2.80 (s, 3H, CH_3_), 5.34 (d, *J* = 2.4 Hz, 2H, OCH_2_), 7.57 (d, *J*_34_ = 7.8 Hz, 1H, H-3), 8.37 (d, *J*_34_ = 7.8 Hz, 1H, H-4). ^13^C-NMR (CDCl_3_, 150 MHz) δ (in ppm): 25.2 (CH_3_), 61.2 (OCH_2_), 77.3 (C=CH), 77.8 (C=CH), 126.1 (C-4a), 128.0 (C-3), 128.2 (C-6), 135.1 (C-4), 146.0 (C-8a), 155.4 (C-7), 165.7 (C-2), 177.7 (C-8), 178.1 (C-5). IR (KBr, cm^−1^) ν_max_: 3233, 2119, 1685, 1664, 1600–1560, 1073. HR-MS (APCI) *m/z*: C_13_H_8_ClNO_3_ [M + H]^+^, Calcd.: 262.0271; Found: 262.0267 (Figure S5).

Compounds **7**–**9** have been described in previously published literature [24,25].

#### 3.1.2. General Procedure for the Synthesis of Dialkoxy Derivatives **13**–**21**

A mixture of 1,4-benzoquinone **1**–**3** (0.441 mmol), potassium carbonate (0.125 g, 0.909 mmol), and alcohol (2.2 eqv., 0.970 mmol) in dry dimethyl sulfoxide (1 mL) was stirred at room temperature for 3 h. Subsequently, CH_2_Cl_2_ was added to the reaction mixture and extracted with water. The organic layer was dried over Na_2_SO_4_. After filtration, the solvents were removed by evaporation under reduced pressure. The residue was purified by silica gel chromatography (chloroform/ethanol, 40:1, v/v) to yield pure products **13**–**21**.

*2,3-Dipropoxy-1,4-naphtoquinon* (**13**): 65 mg (0.238 mmol), yield: 54%, m.p. 92–93 °C. The spectra data was conformed to the literature [38].

*2,3-di(2-Propenoxy)-1,4-naphthoquinon* (**14**): 74 mg (0.27 mmol), yield: 62%, m.p. 135–136 °C. ^1^H-NMR (CDCl_3_, 600 MHz) δ (in ppm): 4.88–4.89 (m, 4H, OCH_2_, OCH_2_), 5.30 (dt, *J* = 1.2 Hz, *J* = 16.2 Hz, 2H, CH=CH_2_, CH=CH_2_), 5.44 (dt, *J* = 1.2 Hz, *J* = 16.2 Hz, 2H, CH=CH_2_, CH=CH_2_), 6.05–6.12 (m, 2H, CH=CH_2_, CH=CH_2_), 7.71–7.73 (m, 2H, H-6, H-7), 8.07-8.09 (m, 2H, H-5, H-8). ^13^C-NMR (CDCl_3_, 150 MHz) δ (in ppm): 74.3 (OCH_2_, OCH_2_), 119.1 (CH=CH_2_, CH=CH_2_), 126.3 (C-5, C-8), 130.9 (CH=CH_2_, CH=CH_2_), 133.0 (C-9, C-10), 133.7 (C-6, C-7), 147.4 (C-2, C-3), 182.1 (C-1, C-4). IR (KBr, cm^−1^) ν_max_: 3077–2887, 1672, 1664, 1595–1577. HR-MS (APCI) *m/z*: C_16_H_14_O_4_ [M + H]^+^, Calcd.: 293.0770; Found: 293.0788 (Figure S6).

*2,3-di(2-Propynoxy)-1,4-naphthoquinon* (**15**): 85 mg (0.38 mmol), yield: 72%, m.p. 100–101 °C. ^1^H-NMR (CDCl_3_, 600 MHz) δ (in ppm): 2.60 (t, *J* = 2.4 Hz, 2H, CH, CH), 5.17 (d, *J* = 2.4 Hz, 2H, OCH_2_), 7.74–7.76 (m, 2H, H-6, H-7), 8.10–8.12 (m, 2H, H-5, H-8). ^13^C-NMR (CDCl_3_, 150 MHz) δ (in ppm): 60.8 (OCH_2_), 60.9 (OCH_2_), 76.9 (C=CH, C=CH), 77.9 (C=CH, C=CH), 126.4 (C-5, C-8), 130.8 (C-9, C-10), 133.9 (C-6, C-7), 146.0 (C-2, C-3), 181.6 (C-1, C-4). IR (KBr, cm^−1^) ν_max_: 3289–3243, 2127, 1668, 1657, 1614–1579. HR-MS (APCI) *m/z*: C_11_H_8_ClNO_3_ [M + H]^+^, Calcd.: 289.0472; Found: 289.0484 (Figure S7).

*2-Methyl-6,7-dipropoxy-5,8-quinolinedione* (**19**): 49 mg (0.17 mmol), yield: 41%, m.p. 145–146 °C. ^1^H-NMR (CDCl_3_, 600 MHz) δ (in ppm): 1.04–1.07 (m, 6H, CH_3_, CH_3_), 1.85 (q, 4H, *J* = 6.6 Hz, *J* = 7.2 Hz, CH_2_CH_3_, CH_2_CH_3_), 2.77 (s, 3H; CH_3_), 4.30–4.33 (m, 4H, OCH_2_, OCH_2_), 7.49 (d, *J*_34_ = 7.8 Hz, 1H, H-3), 8.28 (d, *J*_34_ = 7.8 Hz, 1H, H-4). ^13^C-NMR (CDCl_3_, 150 MHz) δ (in ppm): 22.5 (CH_3_), 24.1 (CH_3_), 28.7 (CH_2_CH_3_, CH_2_CH_3_), 74.7 (OCH_2_), 74.8 (OCH_2_), 124.5 (C-4a), 126.3 (C-3), 133.3 (C-4), 145.4 (C-6), 146.4 (C-7), 147.3 (C-8a), 163.6 (C-2), 179.8 (C-8), 180.2 (C-5). IR (KBr, cm^−1^) ν_max_: 2966–2879, 1669, 1607–1584. HR-MS (APCI) *m/z*: C_16_H_19_NO_4_ [M + H]^+^, Calcd.: 290.1392; Found: 290.1386 (Figure S8).

*2-Methyl-6,7-di(2-propenoxy)-5,8-quinolinedione* (**20**): 63 mg (0.22 mmol), yield: 53%, m.p. 98–99 °C. ^1^H-NMR (CDCl_3_, 600 MHz) δ (in ppm): 2.78 (s, 3H, CH_3_), 4.90–4.92 (m, 4H, 2× OCH_2_), 5.31 (dt, *J* = 1.2 Hz, *J* = 16.2 Hz, 2H, CH=CH_2_, CH=CH_2_), 5.44 (dt, *J* = 1.2 Hz, *J* = 16.2 Hz, 2H, CH=CH_2_, CH=CH_2_), 6.04–6.12 (m, 2H, CH=CH_2_, CH=CH_2_), 7.51 (d, *J*_34_ = 7.8 Hz, 1H, H-3), 8.29 (d, *J*_34_ = 7.8 Hz, 1H, H-4). ^13^C-NMR (CDCl_3_, 150 MHz) δ (in ppm): 24.2 (CH_3_), 73.3 (OCH_2_), 73.4 (OCH_2_), 118.2 (CH=CH_2_), 118.4 (CH=CH_2_), 124.5 (C-4a), 126.4 (C-3), 131.9 (2× CH=CH_2_), 133.4 (C-4), 145.3 (C-6), 146.0 (C-7), 146.8 (C-8a), 163.8 (C-2), 179.6 (C-8), 180.1 (C-5). IR (KBr, cm^−1^) ν_max_: 3081–2852, 1668, 1609–1584. HR-MS (APCI) *m/z*: C_16_H_15_NO_4_ [M + H]^+^, Calcd.: 286.1079; Found: 286.1080 (Figure S9).

*2-Methyl-6,7-di(2-propynoxy)-5,8-qunolinedione* (**21**): 63 mg (0.22 mmol), yield: 54%, m.p. 136–137 °C. ^1^H-NMR (CDCl_3_, 600 MHz) δ (in ppm): 2.59–2.60 (m, 2H, CH, CH), 2.78 (s, 3H, CH_3_), 5.18 (d, *J* = 2.4 Hz, 4H, OCH_2_, OCH_2_), 7.52 (d, *J*_34_ = 7.8 Hz, 1H, H-3), 8.30 (d, *J*_34_ = 7.8 Hz, 1H, H-4). ^13^C-NMR (CDCl_3_, 150 MHz) δ (in ppm): 25.2 (CH_3_), 60.9 (2× OCH_2_), 77.2 (2 C=CH), 77.3 (C=CH), 125.5 (C-4a), 127.6 (C-3), 134.5 (C-4), 145.8 (C-6), 146.2 (C-7), 146.2 (C-8a), 165.1 (C-2), 180.2 (C-8), 180.6 (C-5). IR (KBr, cm^−1^) ν_max_: 3223, 2126, 2116, 1676, 1667, 1610–1560. HR-MS (APCI) *m/z*: C_16_H_11_NO_4_ [M + H]^+^, Calcd.: 282.0766; Found: 282.0773 (Figure S10).

Compounds **16**–**18** have been described in previously published literature [24,25].

#### 3.1.3. General Procedure for the Synthesis of Acyclic Enediyne Derivatives **23**–**25**

The 1,4-benzoquinone **1**–**3** (0.441 mmol) and potassium carbonate were dissolved in dry THF (1 mL). (*Z*)-4-octen-2,6-diynyl-1,8-diol (**22**) (0.060 mg, 0.441 mmol) was then added portion-wise, and the reaction mixture was stirred for 3 h. The solvent was evaporated under reduced pressure. The residue was purified by silica gel chromatography (chloroform/ethanol, 15:1, v/v) to yield pure products **23**–**25**.

*2-Chloro-3-(8-hydroxy-4-octen-2,6-diynyloxy)-1,4-naphthoquinon* (**23**): 73 mg (0.23 mmol), yield: 51%, m.p. 100–102 °C. ^1^H-NMR (CDCl_3_, 600 MHz) δ (in ppm): 4.47 (s, 2H, CH_2_OH), 5.53 (s, 2H, OCH_2_), 5.82–5.94 (m, 2H, CH, CH), 7.81–7.84 (m, 2H, H-6, H-7), 8.14–8.22 (m, 2H, H-5, H-8). ^13^C-NMR (CDCl_3_, 150 MHz) δ (in ppm): 51.5 (CH_2_OH), 62.0 (OCH_2_), 82.2 (C≡C), 85.9 (C≡C), 90.3 (C≡C), 96.1 (C≡C), 118.4 (CH=CH, CH=CH), 120.8 (CH=CH, CH=CH), 127.1 (C-8, C-5), 127.3 (C-3), 130.7 (C-9), 131.1 (C-10), 134.3 (C-6), 134.5 (C-7), 155.2 (C-2), 178.6 (C-1), 179.8 (C-4). IR (KBr, cm^−1^) ν_max_: 3497–3323, 2955–2858, 1677, 1599–1574, 1291, 1094. HR-MS (APCI) *m/z*: C_18_H_11_ClO_4_ [M + H]^+^, Calcd.: 327.0424; Found: 327.0412 (Figure S11).

*6-Chloro-7-(8-hydroxy-4-octen-2,6-diynyloxy)-5,8-quinolinedione* (**24**): 55 mg (0.18 mmol), yield: 41%, m.p. 95–96 °C. ^1^H-NMR (CDCl_3_, 600 MHz) δ (in ppm): 4.54 (d, *J* = 2.4 Hz, 2H, CH_2_OH), 5.62 (d, *J* = 2.4 Hz, 2H, OCH_2_), 5.76 (dt, *J* = 2.4 Hz, *J* = 13.2 Hz, 1H, CH), 5.88 (dt, *J* = 2.4 Hz, *J* = 13.2 Hz, 1H, CH), 7.76 (dd, *J*_23_ = 4.8 Hz, *J*_34_ = 7.8 Hz, 1H, H-3), 8.54 (dd, *J*_24_ = 1.8 Hz, *J*_34_ = 7.8 Hz, 1H, H-4), 9.02 (dd, *J*_24_ = 1.8 Hz, *J*_23_ = 4.8 Hz, 1H, H-2). ^13^C-NMR (CDCl_3_, 150 MHz) δ (in ppm): 50.3 (CH_2_OH), 61.1 (OCH_2_), 80.4 (C≡C), 85.3 (C≡C), 88.6 (C≡C), 96.6 (C≡C), 117.1 (CH=CH, CH=CH), 121.8 (CH=CH, CH=CH), 127.1 (C-3), 127.2 (C-6), 129.2 (C-4a), 134.3 (C-4), 146.1 (C-8a), 153.3 (C-2), 154.7 (C-7), 176.4 (C-8), 176.5 (C-5). IR (KBr, cm^−1^) ν_max_: 3305, 3076–2864, 1692, 1680, 1596–1560, 1253, 1097. HR-MS (APCI) *m/z*: C_17_H_10_ClNO_4_ [M+H]^+^, Calcd: 328.0377; Found: 328.0367 (Figure S12).

*6-Chloro-7-(8-hydroxy-4-octen-2,6-diynyloxy)-2-methyl-5,8-quinolinedione (**25**):* 51 mg (0.15 mmol), yield: 36%, m.p. 115–116 °C. ^1^H-NMR (CDCl_3_, 600 MHz) δ (in ppm): 2.80 (s, 3H, CH_3_), 4.55 (d, *J* = 2.4 Hz, 2H, CH_2_OH), 5.60 (d, *J* = 2.4 Hz, 2H, OCH_2_), 5.74 (dt, *J* = 2.4 Hz, *J* = 13.2 Hz, 1H, CH), 5.88 (dt, *J* = 2.4 Hz, *J* = 13.2 Hz, 1H, CH), 7.85 (d, *J*_34_ = 7.8 Hz, 1H, H-3), 8.38 (d, *J*_34_ = 7.8 Hz, 1H, H-4). ^13^C-NMR (CDCl_3_, 150 MHz) δ (in ppm): 24.7 (CH_3_), 51.2 (CH_2_OH), 61.9 (OCH_2_), 81.3 (C≡C), 86.2 (C≡C), 89.8 (C≡C), 97.6 (C≡C), 118.3 (CH=CH, CH=CH), 122.8 (CH=CH, CH=CH), 126.1 (C-4a), 128.3 (C-3), 130.2 (C-6), 135.3 (C-4), 145.7 (C-8a), 155.4 (C-7), 165.2 (C-2), 177.5 (C-8), 177.9 (C-5). IR (KBr, cm^−1^) ν_max_: 3379–3299, 2929–2860, 1692, 1669, 1603–1560, 1258, 1098. HR-MS (APCI) *m/z*: C_18_H_12_ClNO_4_ [M + H]^+^, Calcd.: 342.0533; Found: 342.0551 (Figure S13).

#### 3.1.4. General Procedure for the Synthesis of Cyclic Enediyne Derivatives **26**–**28**

1,4-Benzoquinone (**1**–**3**) (0.441 mmol) and potassium carbonate was dissolved in dry DMSO (1 mL). (*Z*)-4-octen-2,6-diynyl-1,8-diol (**22**) (0.060 mg, 0.441 mmol) was then added portion-wise, and the reaction mixture was stirred for 3 h. Subsequently, CH_2_Cl_2_ was added to the reaction mixture, and water was extracted. The organic layer was dried over Na_2_SO_4_. After filtration, the solvents were removed by evaporation under reduced pressure. The residue was purified by silica gel chromatography (chloroform/ethanol, 15:1, v/v) to yield pure products **26**–**28**.

*8-Octen-6,10-diynyl-1,4-dioxocyclododeca[2,3-g]-1,4-naphthoquinon (**26**):* 54 mg (0.19 mmol), yield: 42%, m.p. 73–74 °C. ^1^H-NMR (CDCl_3_, 600 MHz) δ (in ppm): 5.51 (s, 4H, 2× CH_2_), 5.85 (s, 2H, 2× CH), 7.34–7.75 (m, 2H, H-6, H-7), 8.07–8.08 (m, 2H, H-5, H-8). ^13^C-NMR (CDCl_3_, 150 MHz) δ (in ppm): 60.3 (CH_2_), 87.2 (C≡C), 91.2 (C≡C), 119.6 (CH=CH, CH=CH), 125.4 (C-5, C-8), 123.0 (C-9, C-10), 132.9 (C-6, C-7), 147.1 (C-2, C-3), 181.1 (C-1, C-4). IR (KBr, cm^−1^) ν_max_: 2961–2853, 1665, 1655, 1604–1577, 1262. HR-MS (APCI) *m/z*: C_18_H_10_O_4_ [M + H]^+^, Calcd.: 291.0657; Found: 291.0621 (Figure S14).

*8-Octen-6,10-diynyl-1,4-dioxocyclododeca[2,3-g]-5,8-quinolinedione (**27**):* 49 mg (0.18 mmol), yield: 42%, m.p. 56–58°C. ^1^H-NMR (CDCl_3_, 600 MHz) δ (in ppm): 5.51 (s, 2H, OCH_2_), 5.54 (s, 2H, OCH_2_), 5.87 (d, *J* = 3.0 Hz, 2H, CH, CH), 7.69 (dd, *J*_23_ = 4.8 Hz, *J*_34_ = 7.8 Hz, 1H, H-3), 8.41 (dd, *J*_24_ = 1.8 Hz, *J*_34_ = 7.8 Hz, 1H, H-4), 9.04 (dd, *J*_24_ = 1.8 Hz, *J*_23_ = 4.8 Hz, 1H, H-2). ^13^C-NMR (CDCl_3_, 150 MHz) δ (in ppm): 60.6 (OCH_2_, OCH_2_), 87.4 (C≡C), 87.5 (C≡C), 90.7 (C≡C), 91.1 (C≡C), 119.4 (CH=CH), 119.9 (CH=CH), 126.7 (C-3), 126.8 (C-4a), 133.4 (C-4), 145.7 (C-8a), 146.9 (C-7), 148.2 (C-6), 153.7 (C-2), 179.3 (C-8), 180.2 (C-5). IR (KBr, cm^−1^) ν_max_: 2929–2853, 1682, 1668, 1616–1575, 1262. HR-MS (APCI) *m/z*: C_17_H_9_NO_4_ [M + H]^+^, Calcd.: 292.0610; Found: 292.0618 (Figure S15).

*8-Octen-6,10-diynyl-1,4-8-octen-6,10-diynyl-1,4-dioxocyclododeca[2,3-g]-2-methyl-5,8-quinolinedione (**28**):* 41 mg (0.15 mmol), yield: 35%, m.p. 45–47 °C. ^1^H-NMR (CDCl_3_, 600 MHz) δ (in ppm): 2.79 (s, 3H, CH_3_), 5.50 (s, 2H, OCH_2_), 5.51 (s, 2H, OCH_2_), 5.85 (d, *J* = 3.0 Hz, 2H, CH, CH), 7.53 (d, *J*_34_ = 7.8 Hz, 1H, H-3), 8.28 (d, *J*_34_ = 7.8 Hz, 1H, H-4). ^13^C-NMR (CDCl_3_, 150 MHz) δ (in ppm): 25.2 (CH_3_), 61.5 (OCH_2_, OCH_2_), 88.4 (C≡C), 88.5 (C≡C), 91.8 (C≡C), 92.1 (C≡C), 120.4 (CH=CH), 120.9 (CH=CH), 125.7 (C-4a), 127.6 (C-3), 134.5 (C-4), 146.3 (C-6), 147.6 (C-7), 148.8 (C-8a), 165.1 (C-2), 180.7 (C-8), 181.3 (C-5). IR (KBr, cm^−1^) ν_max_: 2964–2854, 1683, 1663, 1584, 1262. HR-MS (APCI) *m/z*: C_18_H_11_NO_4_ [M + H]^+^, Calcd.: 306.0766; Found: 306.0771 (Figure S16).

### 3.2. Biological Activity

#### 3.2.1. Cell Culture

Biological activity of the tested compounds was assessed in vitro using cultured cell lines: melanoma C-32 (ATCC, Rockville, MD, USA), glioblastoma SNB-19 (DSMZ, Braunschweig, Germany), breast cancer MDA-MB-231 (ATCC, Rockville, MD, USA), and normal human fibroblasts derived from foreskin HFF-1 (ATCC, Rockville, MD, USA). Cell cultures were maintained using DMEM (Lonza, Basel, Switzerland) supplemented with 10% fetal bovine serum (FBS) (Biological Industries Cromwell, CT, USA) and a mixture of penicillin (10,000 U/mL) and streptomycin (10 mg/mL) (Lonza, Basel, Switzerland).

#### 3.2.2. Effect of Compounds on Number and Viability of Cells

Cells were cultured using 96-well plates (Nunc Thermo Fisher Scientific, Waltham, MA, USA). Cells were seeded (5 × 10^3^ cells/well) and incubated for 24 h in a standard incubator (37 °C, 5% CO_2_, relative humidity 95%). Subsequently, the medium was replaced with a fresh aliquot containing an examined compound (0.01; 0.05; 0.1; 0.5; 1; 5; 10; 50; 100 µg/mL), and cells were further incubated for another 72 h. Upon the conclusion of incubation, a CVDE test (Aniara, West Chester, OH, USA) using crystal violet was performed to assess the relative number of cells in the culture. A WST-1 test (Roche) was performed to examine the metabolic activity of cells and test for the presence of LDH in the medium (Roche Diagnostics GmbH, Mannheim, Germany), allowing for the assessment of the relative number of dead cells (i.e., cytotoxicity of the examined compound). UVM340 microplate readers (BIOGENET, Józefów, Poland) were used to read absorbance values.

#### 3.2.3. WST-1 Test

WST-1 colorimetric assay for cell proliferation (Roche Diagnostics GmbH, Mannheim, Germany, reagent kit cat. 11644807001) is based on the viable cells’ ability to cleave the bright red-colored stable tetrazolium salt WST-1 to dark red soluble formazan. This bioreduction occurs under the influence of mitochondrial dehydrogenases (depends mostly on production of NAD(P)H in viable cells). The amount of formazan dye formed correlates directly with the number of metabolically active cells in the culture and is measured by absorbance (λ = 450 nm) following 1 h incubation of cells with the reagent.

## 4. Conclusions

In this study, we identified antiproliferative agents among a series of mono and dialkoxy derivatives containing 1,4-naphthoquinone, 5,8-quionolinedione, or 2-methyl-5,8-quinolinedione subunits. The general trend of the antitumor activity showed that the introduction of an alkoxy substituent to the 1,4-benzoquinone moiety leads to an increase in the activity against all tested cancer cell lines. In the series of enediyne compounds, the best activity against melanoma (C-32) and glioblastoma (SNB-19) cell lines was exhibited by 6-chloro-7-(8-hydroxy-4-octen-2,6-diynyloxy)-5,8-quinolinedione. The analysis of the calculated physicochemical parameters suggested that the synthesized derivatives were characterized by a high oral bioavailability and a low pass through blood–brain barrier.

## Figures and Tables

**Table 1:** The antiproliferative activity of Compounds **1**–**21** and cisplatin as a reference compound.

Compound	Human Cell Line/IC_50_ ± SD (µM)			
	C-32	SNB-19	MDA-MB-231	HFF-1
**1**	25.0 ± 0.3	26.6 ± 0.5	31.9 ± 2.7	15.6 ± 0.3
**2 ^a^**	22.8 ± 0.7	26.2 ± 0.8	25.1 ± 3.1	15.7 ± 0.5
**3**	10.5 ± 0.8	15.9 ± 0.3	23.3 ± 0.3	10.0 ± 1.4
**4**	16.8 ± 0.3	18.4 ± 1.7	1.4 ± 0.3	14.8 ± 0.9
**5**	4.0 ± 0.1	3.8 ± 0.1	6.9 ± 1.9	3.8 ± 0.8
**6**	3.2 ± 0.6	8.4 ± 1.2	21.0 ± 1.5	3.9 ± 0.5
**7 ^a^**	0.20 ± 0.1	0.4 ± 0.1	22.4 ± 2.7	Neg
**8 ^a^**	0.28 ± 0.1	0.3 ± 0.1	17.2 ± 2.8	Neg
**9 ^b^**	0.32 ± 0.1	20.4 ± 0.2	21.4 ± 3.4	3.3 ± 0.3
**10**	26.8 ± 1.9	19.8 ± 1.4	22.2 ± 1.4	15.8 ± 1.8
**11**	168.0 ± 6.2	33.8 ± 1.3	24.6 ± 2.9	20.1 ± 1.2
**12**	207.6 ± 4.6	19.5 ± 1.0	17.2 ± 2.5	14.0 ± 2.4
**13**	22.6 ± 0.8	19.9 ± 1.4	22.6 ± 2.3	10.8 ± 0.5
**14**	10.6 ± 0.6	19.6 ± 1.0	17.9 ± 0.8	4.4 ± 0.9
**15**	21.7 ± 0.6	18.6 ± 0.7	26.1 ± 0.3	13.5 ± 1.3
**16 ^a^**	1.1 ± 0.1	0.7 ± 0.02	3.0 ± 0.3	Neg
**17 ^a^**	1.5 ± 0.1	0.2 ± 0.1	12.6 ± 2.7	Neg
**18 ^b^**	0.3 ± 0.1	21.7 ± 1.1	1.8 ± 0.1	12.6 ± 0.9
**19**	131.4 ± 2.4	25.7 ± 1.4	71.3 ± 3.9	17.8 ± 1.8
**20**	18.9 ± 1.7	16.6 ± 3.0	26.8 ± 1.2	0.6 ± 0.2
**21**	21.1 ± 1.2	24.5 ± 1.1	23.2 ± 1.5	14.5 ± 2.6
**cisplatin**	16.4 ± 1.2	18.9 ± 0.4	25.5 ± 0.2	9.1 ± 1.8

Note: Neg: negative in the concentration used. ^a^ [24], ^b^ [25].

**Table 2:** The antiproliferative activity of Compounds **22**–**26** and cisplatin as a reference compound.

Compound	Human Cell Line/IC_50_ ± SD (µM)			
	C-32	SNB-19	MDA-MB-231	HFF-1
**22**	158.7 ± 4.6	250.4 ± 4.3	Neg	260.0 ± 5.6
**23**	144.6 ± 4.8	186.0 ± 5.0	22.5 ± 2.4	97.6 ± 4.0
**24**	3.1 ± 0.1	2.4 ± 0.1	110.2 ± 8.1	Neg
**25**	115.6 ± 2.9	168.7 ± 4.8	16.5 ± 1.9	27.1 ± 1.1
**26**	30.3 ± 3.1	22.8 ± 1.4	2.0 ± 0.2	17.5 ± 1.3
**27**	48.4 ± 8.9	31.5 ± 2.5	25.1 ± 2.4	13.0 ± 3.4
**28**	155.4 ± 3.7	157.1 ± 5.7	173.5 ± 5.9	27.8 ± 1.2
**cisplatin**	16.4 ± 1.2	18.9 ± 0.4	25.5 ± 0.2	9.1 ± 1.8

Note: Neg: negative in the concentration used.

## Supplementary Materials

The Supplementary Materials are available online.
