# Supplementary material for: Alkoxy and Enediyne Derivatives Containing 1,4-Benzoquinone Subunits—Synthesis and Antitumor Activity
Source: Molecules. 2017 Mar 11;22(3):447. doi: 10.3390/molecules22030447 (PMC6155387; doi:10.3390/molecules22030447)
Supplement: Supplementary file 1 [file molecules-22-00447-s001.pdf]

Supplementary Materials

Alkoxy and Enediyne Derivatives Containing 1.4-Benzoquinone Subunits—Synthesis and Antitumor Activity

Monika Kadela-Tomanek, Ewa Bębenek, Elwira Chrobak, Małgorzata Latocha, Stanisław Boryczka

Table S1: The selectivity index (SI) value for Compounds **1-28** and cisplatin.

Table S2: The parameters determined by computational methods such as lipophilicity (cLogP), molecular mass (M), number of donors (nHD) and acceptors (nHA) of hydrogen bonds, number of rotatable bonds (nRTB), topological polar surface area (PSA), and penetration drug by BBB (log BB).

Figure S1: (a) ^{1}H-NMR spectrum, (b) ^{13}C-NMR spectrum, (c) IR spectrum, (d) HR-MS spectrum of 2-chloro-3-(2-propenoxy)-1,4-naphthoquinon (**5**).

Figure S2: (a) ^{1}H-NMR spectrum, (b) ^{13}C-NMR spectrum, (c) IR spectrum, (d) HR-MS spectrum of 2-chloro-3-(2-propynoxy)-1,4-naphthoquinon (**6**).

Figure S3: (a) ^{1}H-NMR spectrum, (b) ^{13}C-NMR spectrum, (c) IR spectrum, (d) HR-MS spectrum of 6-chloro-2-methyl-7-propoxy-5,8-quinolinedione (**10**).

Figure S4: (a) ^{1}H-NMR spectrum, (b) ^{13}C-NMR spectrum, (c) IR spectrum, (d) HR-MS spectrum of 6-chloro-2-methyl-7-(2-propenoxy)-5,8-quinolinedione (**11**).

Figure S5: (a) ^{1}H-NMR spectrum, (b) ^{13}C-NMR spectrum, (c) IR spectrum, (d) HR-MS spectrum of 6-chloro-2-methyl-7-(2-propynoxy)-5,8-quinolinedione (**12**).

Figure S6: (a) ^{1}H NMR spectrum, (b) ^{13}C NMR spectrum, (c) IR spectrum, (d) HR-MS spectrum of 2,3-di(2-propenoxy)-1,4-naphthoquinon (**14**).

Figure S7: (a) ^{1}H-NMR spectrum, (b) ^{13}C-NMR spectrum, (c) IR spectrum, (d) HR-MS spectrum of 2,3-di(2-propynoxy)-1,4-naphthoquinon (**15**).

Figure S8: (a) ^{1}H-NMR spectrum, (b) ^{13}C-NMR spectrum, (c) IR spectrum, (d) HR-MS spectrum of 2-methyl-6,7-dipropoxy-5,8-quinolinedione (**19**).

Figure S9: (a) ^{1}H-NMR spectrum, (b) ^{13}C-NMR spectrum, (c) IR spectrum, (d) HR-MS spectrum of 2-methyl-6,7-di(2-propenoxy)-5,8-quinolinedione (**20**).

Figure S10: (a) ^{1}H-NMR spectrum, (b) ^{13}C-NMR spectrum, (c) IR spectrum, (d) HR-MS spectrum of 2-methyl-6,7-di(2-propynoxy)-5,8-qunolinedione (**21**).

Figure S11: (a) ^{1}H-NMR spectrum, (b) ^{13}C-NMR spectrum, (c) IR spectrum, (d) HR-MS spectrum of 2-chloro-3-(8-hydroxy-4-octen-2,6-diynyloxy)-1,4-naphtoquinon (**23**).

Figure S12: (a) ^{1}H-NMR spectrum, (b) ^{13}C-NMR spectrum, (c) IR spectrum, (d) HR-MS spectrum of 6-chloro-7-(8-hydroxy-4-octen-2,6-diynyloxy)-5,8-quinolinedione (**24**).

Figure S13: (a) ^{1}H-NMR spectrum, (b) ^{13}C-NMR spectrum, (c) IR spectrum, (d) HR-MS spectrum6-chloro-7-(8-hydroxy-4-octen-2,6-diynyloxy)-2-methyl-5,8-quinolinedione (**25**).

Figure S14: (a) ^{1}H-NMR spectrum, (b) ^{13}C-NMR spectrum, (c) IR spectrum, (d) HR-MS spectrum of 8-octen-6,10-diynyl-1,4-dioxocyclododeca[2,3-g]-1,4-naphthoquinon (**26**).

Figure S15: (a) ^{1}H-NMR spectrum, (b) ^{13}C-NMR spectrum, (c) IR spectrum, (d) HR-MS spectrum of 8-octen-6,10-diynyl-1,4-dioxocyclododeca[2,3-g]-5,8-quinolinedione (**27**).

Figure S16: (a) ^{1}H-NMR spectrum, (b) ^{13}C-NMR spectrum, (c) IR spectrum, (d) HR-MS spectrum of 8-octen-6,10-diynyl-1,4-8-octen-6,10-diynyl-1,4-dioxocyclododeca[2,3-g]-2-methyl-5,8-quinolinedione (**28**).

Table S1: The selectivity index (SI) value for Compounds **1-28** and cisplatin.

Compound	SI ($IC_{50\ HFF-1}/IC_{50\ cancer\ line}$)		
	**C-32**	**SNB-19**	**MDA-MB-231**
**1**	0.62	0.59	0.49
**2**	0.69	0.60	0.63
**3**	0.95	0.63	0.43
**4**	0.88	0.80	10.57
**5**	0.95	1.00	0.55
**6**	1.22	0.46	0.19
**7**	-	-	-
**8**	-	-	-
**9**	10.31	0.16	0.15
**10**	0.59	0.80	0.71
**11**	0.12	0.59	0.82
**12**	0.07	0.72	0.81
**13**	0.48	0.54	0.48
**14**	0.42	0.22	0.25
**15**	0.62	0.73	5.19
**16**	-	-	-
**17**	-	-	-
**18**	50.40	0.58	7.00
**19**	0.14	0.69	0.25
**20**	0.03	0.04	0.02
**21**	0.69	0.59	0.63
**22**	1.64	1.04	1.85
**23**	0.67	0.52	0.23
**24**	-	-	-
**25**	0.23	0.16	0.61
**26**	0.58	0.77	0.11
**27**	0.27	0.41	1.93
**28**	0.18	0.18	6.25
**cisplatin**	0.55	0.48	2.80

Table S2: The parameters determined by computational methods such as lipophilicity (cLogP), molecular mass (M), number of donors (nHD) and acceptors (nHA) of hydrogen bonds, number of rotatable bonds (nRTB), topological polar surface area (PSA), and penetration drug by BBB (log BB).

Compound	M [g/mol]	clopP	nHA	nHD	nRTB	PSA [Å^2]	logBB
**1**	227	2.55	2	0	0	34.14	- 0.45
**2**	242	2.69	3	0	0	47.03	- 0.61
**3**	227	2.17	3	0	0	47.03	- 0.25
**4**	250	2.33	3	0	3	43.38	- 0.36
**5**	248	2.59	3	0	3	43.38	- 0.16
**6**	246	2.21	3	0	2	43.38	- 0.71
**7**	266	2.41	4	0	3	56.27	- 0.42
**8**	264	2.29	4	0	3	56.27	- 0.14
**9**	263	1.75	4	0	2	56.27	- 0.85
**10**	251	1.95	4	0	3	56.27	- 0.19
**11**	249	1.57	4	0	3	56.27	- 0.04
**12**	247	1.15	4	0	2	56.27	- 0.76
**13**	275	2.57	4	0	6	52.61	- 0.06
**14**	271	2.28	4	0	6	52.61	- 0.06
**15**	266	1.77	4	0	4	52.61	- 1.25
**16**	290	2.20	5	0	6	65.50	- 0.21
**17**	287	1.84	5	0	6	65.50	- 0.10
**18**	284	1.49	5	0	4	65.50	- 1.27
**19**	278	2.06	5	0	6	65.50	- 0.06
**20**	271	1.84	5	0	6	65.50	- 0.06
**21**	367	1.02	5	0	4	65.50	- 1.16
**22**	136	1.01	2	2	0	40.46	- 0.57
**23**	326	3.20	4	1	2	63.60	- 0.54
**24**	328	2.57	5	1	2	76.50	- 0.83
**25**	342	2.23	5	1	2	76.50	- 0.69
**26**	291	3.23	4	0	0	52.61	- 0.41
**27**	292	2.45	5	0	0	65.50	- 0.70
**28**	306	2.15	5	0	0	65.50	- 0.57

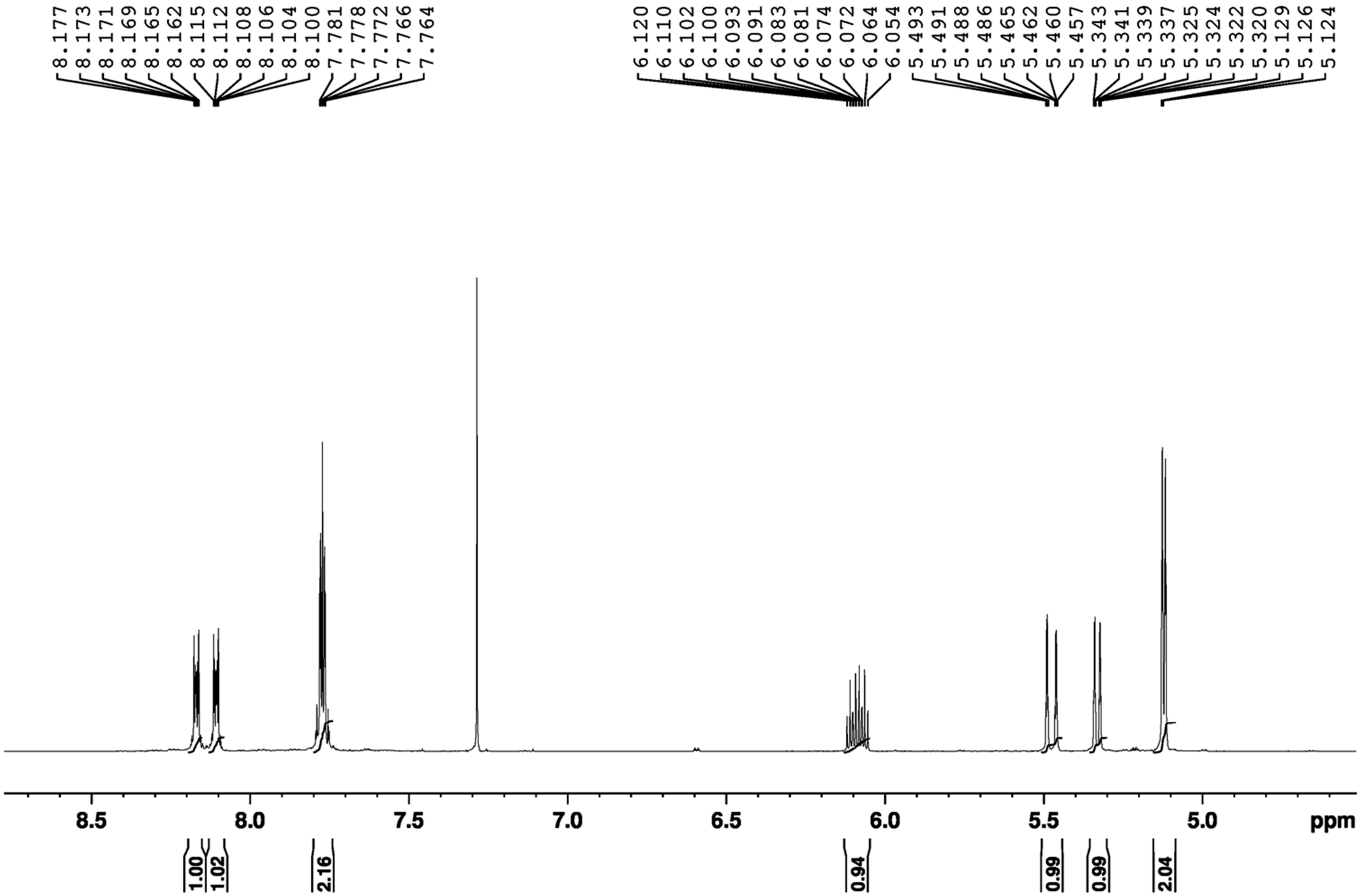

Figure S1 (a): ^{1}H-NMR spectrum of 2-chloro-3-(2-propenoxy)-1,4-naphthoquinon (**5**).

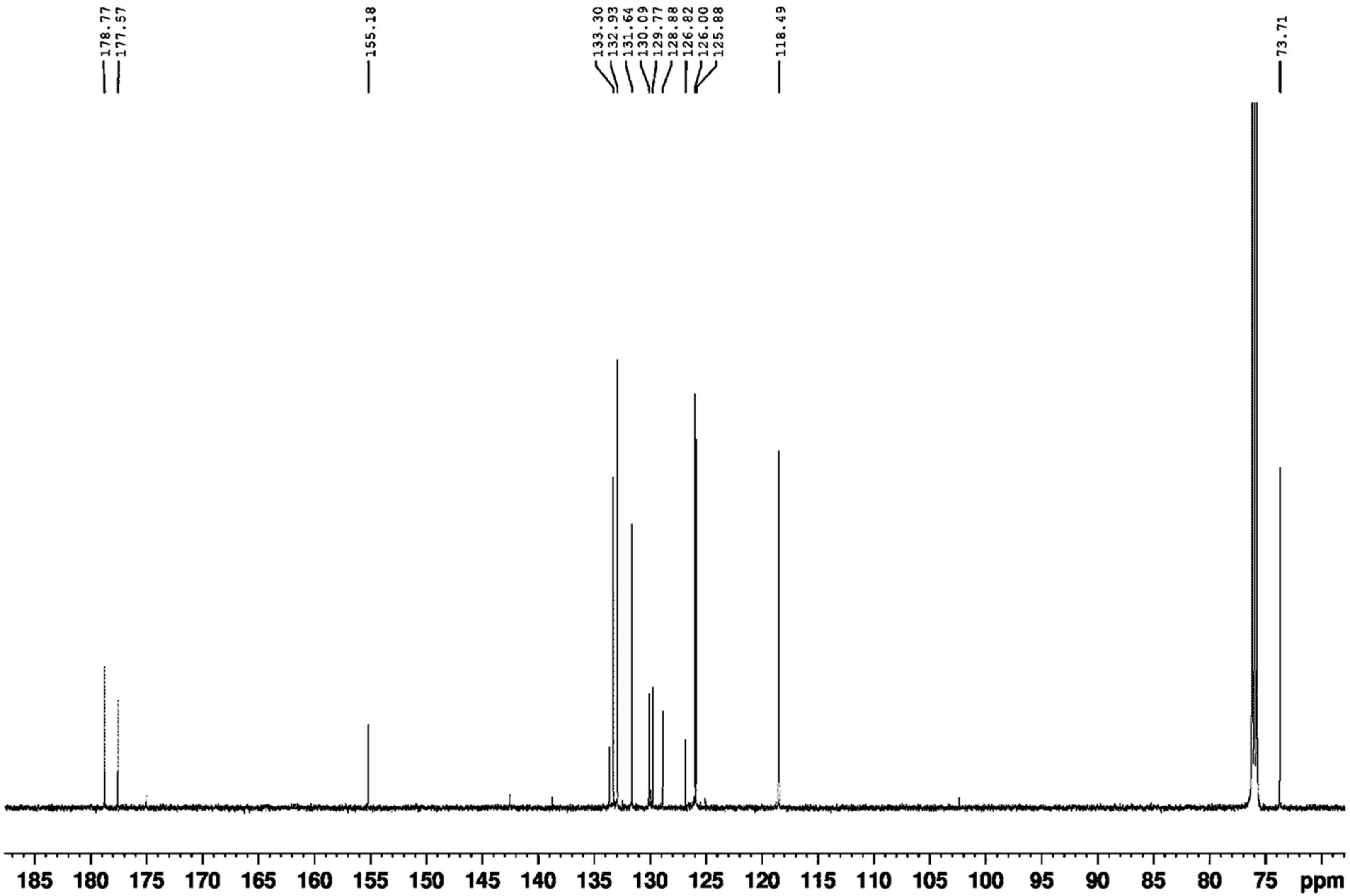

Figure S1 (b): ^{13}C-NMR spectrum of 2-chloro-3-(2-propenoxy)-1,4-naphthoquinon (**5**).

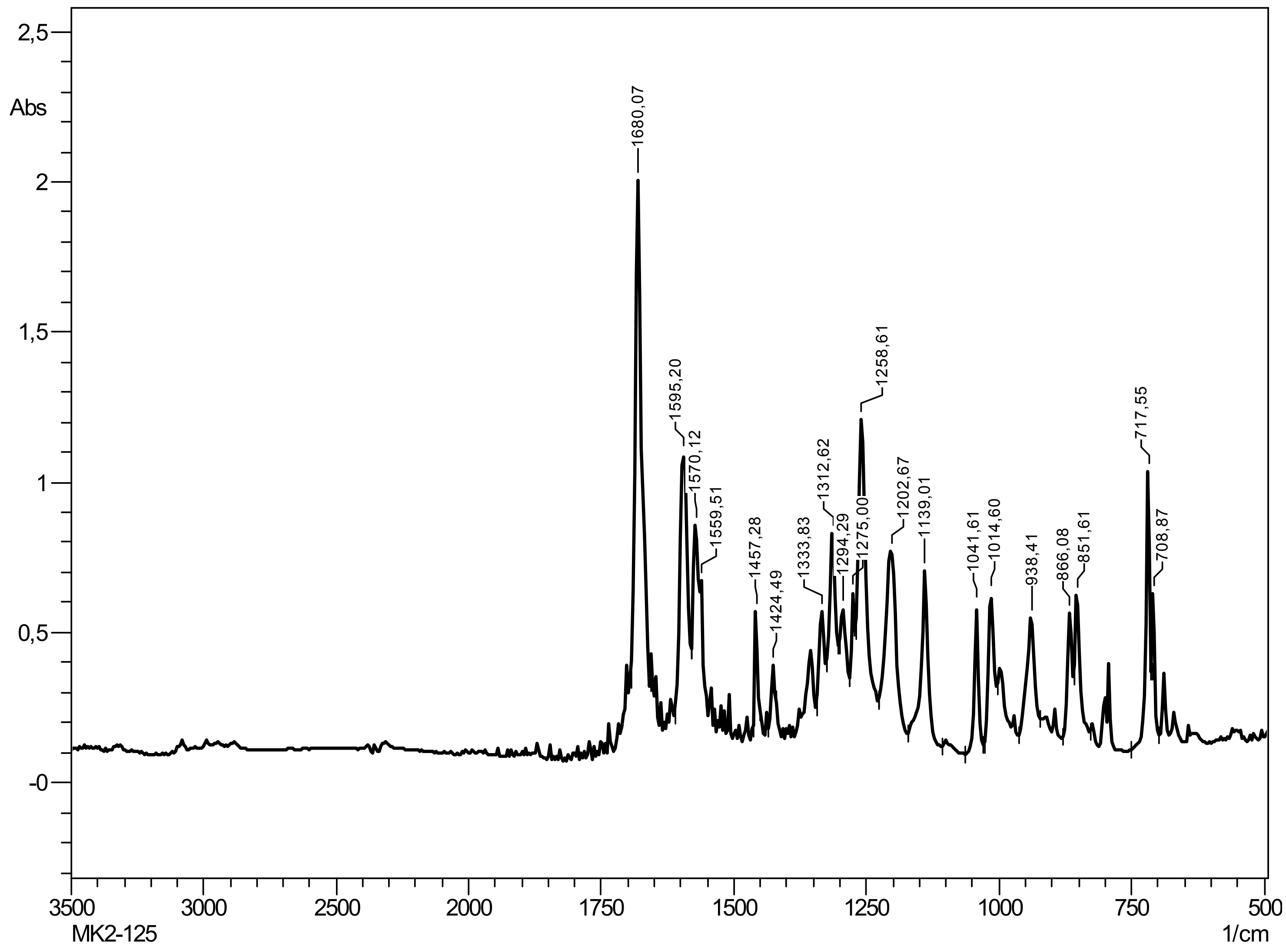

Figure S1 (c): IR spectrum of 2-chloro-3-(2-propenoxy)-1,4-naphthoquinon (**5**).

Compound Spectrum List Report

Analysis Info

Analysis Name	D:\Data\mk5.d	Acquisition Date	2/17/2017 11:21:14 AM
Method	low_mass.m	Operator	KM
Sample Name	TM Low concentration	Instrument	impact II 1825265.10082
Comment			

Acquisition Parameter

Source Type	ESI	Ion Polarity	Positive	Set Nebulizer	0.3 Bar
Focus	Active	Set Capillary	4000 V	Set Dry Heater	240 °C
Scan Begin	100 m/z	Set End Plate Offset	-500 V	Set Dry Gas	4.0 l/min
Scan End	1000 m/z	Set Charging Voltage	2000 V	Set Divert Valve	Source
		Set Corona	0 nA	Set APCI Heater	0 °C

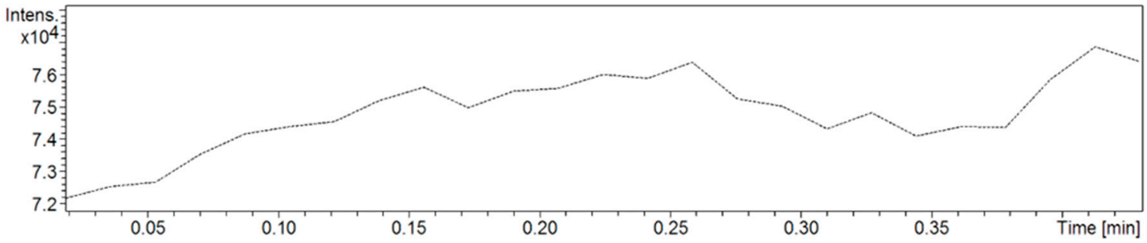

+MS, 0.0-0.4min #1-25

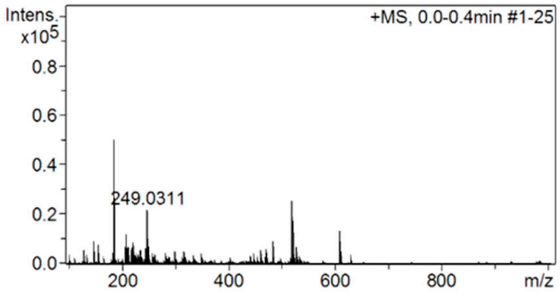

#	m/z	Res.	S/N	I	I %	FWHM
1	249.0311	20814	950.2	22188	100.0	0.0120

(d)

Figure S1 (d): HR-MS spectrum of 2-chloro-3-(2-propenoxy)-1,4-naphthoquinon (**5**).

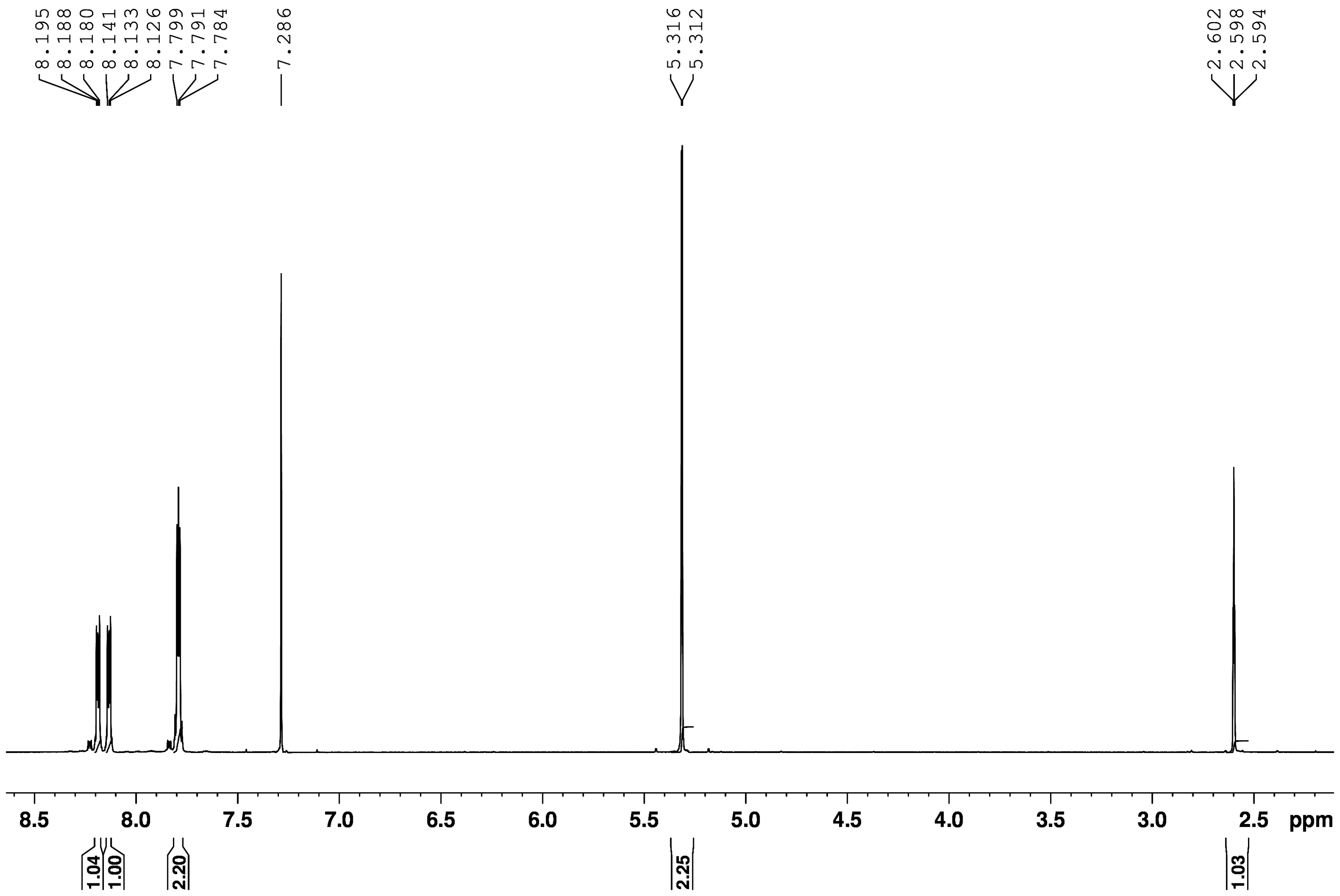

Figure S2 (a): ^{1}H-NMR spectrum of 2-chloro-3-(2-propynoxy)-1,4-naphtoquinon (**6**).

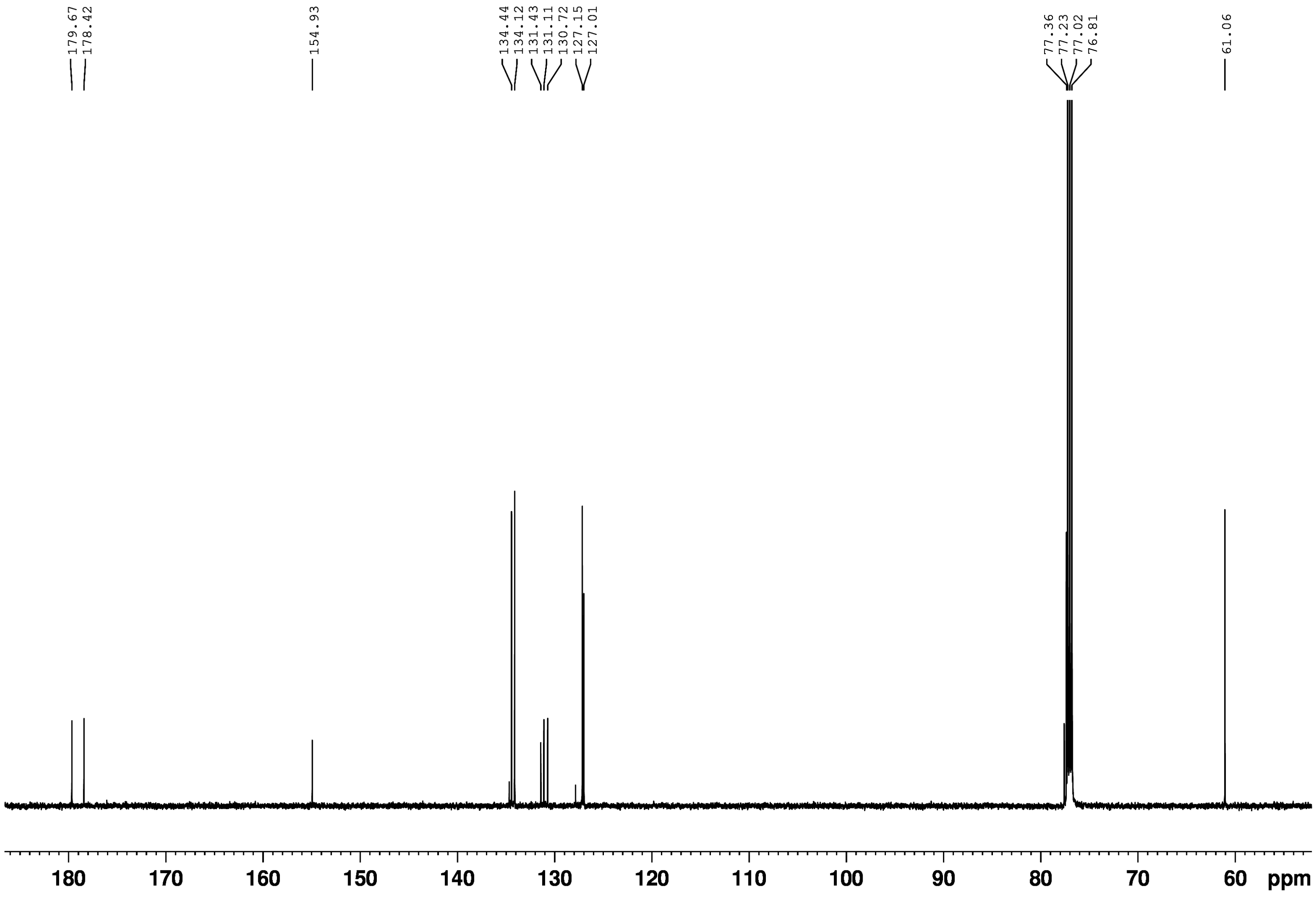

Figure S2 (b): ^{13}C-NMR spectrum of 2-chloro-3-(2-propynoxy)-1,4-naphtoquinon (**6**).

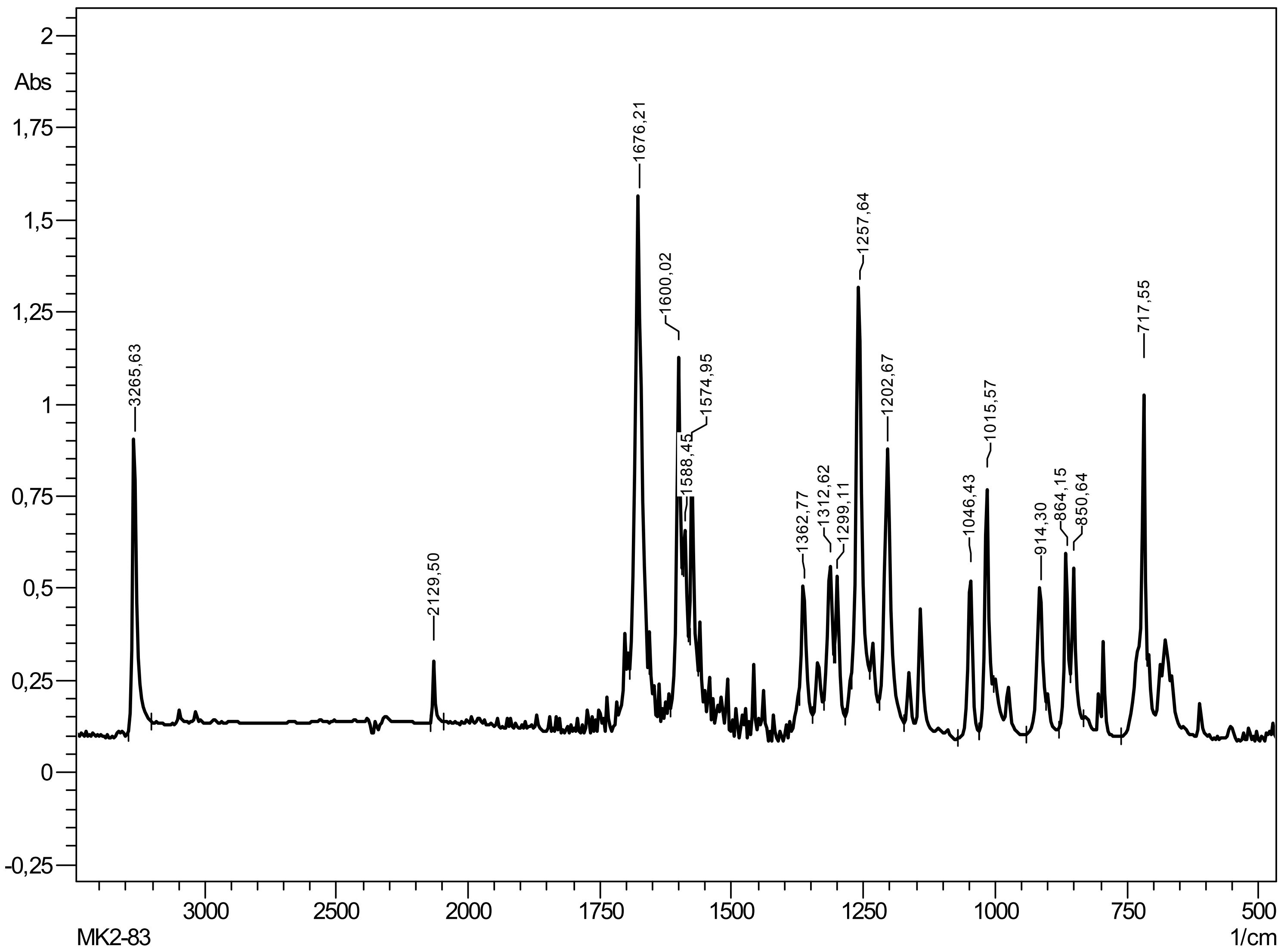

Figure S2 (c): HR-MS spectrum of 2-chloro-3-(2-propynoxy)-1,4-naphtoquinon (**6**).

Compound Spectrum List Report

Analysis Info

Analysis Name	D:\Data\mk6.d	Acquisition Date	2/17/2017 11:25:36 AM	
Method	low_mass.m	Operator	KM	
Sample Name	TM Low concentration	Instrument	impact II	1825265.10082
Comment				

Acquisition Parameter

Source Type	ESI	Ion Polarity	Positive	Set Nebulizer	0.3 Bar
Focus	Active	Set Capillary	4000 V	Set Dry Heater	240 °C
Scan Begin	100 m/z	Set End Plate Offset	-500 V	Set Dry Gas	4.0 l/min
Scan End	1000 m/z	Set Charging Voltage	2000 V	Set Divert Valve	Source
		Set Corona	0 nA	Set APCI Heater	0 °C

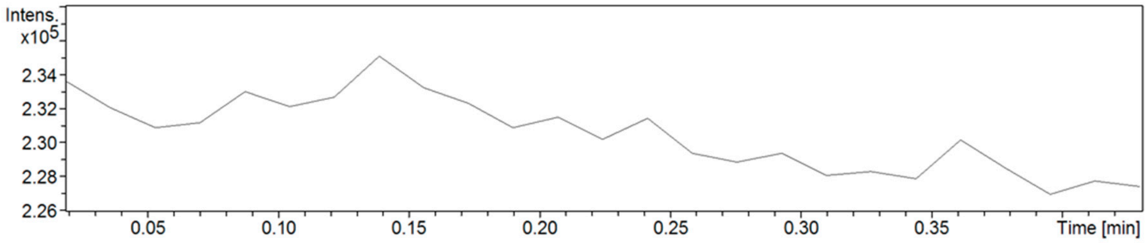

+MS, 0.0-0.4min #1-25

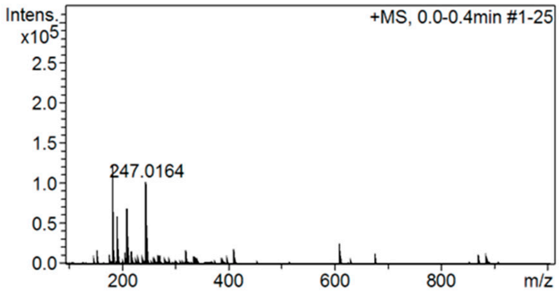

#	m/z	Res.	S/N	I	I %	FWHM
1	247.0164	24713	3330.4	101661	100.0	0.0100

Figure S2 (d): HR-MS spectrum of 2-chloro-3-(2-propynoxy)-1,4-naphtoquinon (**6**).

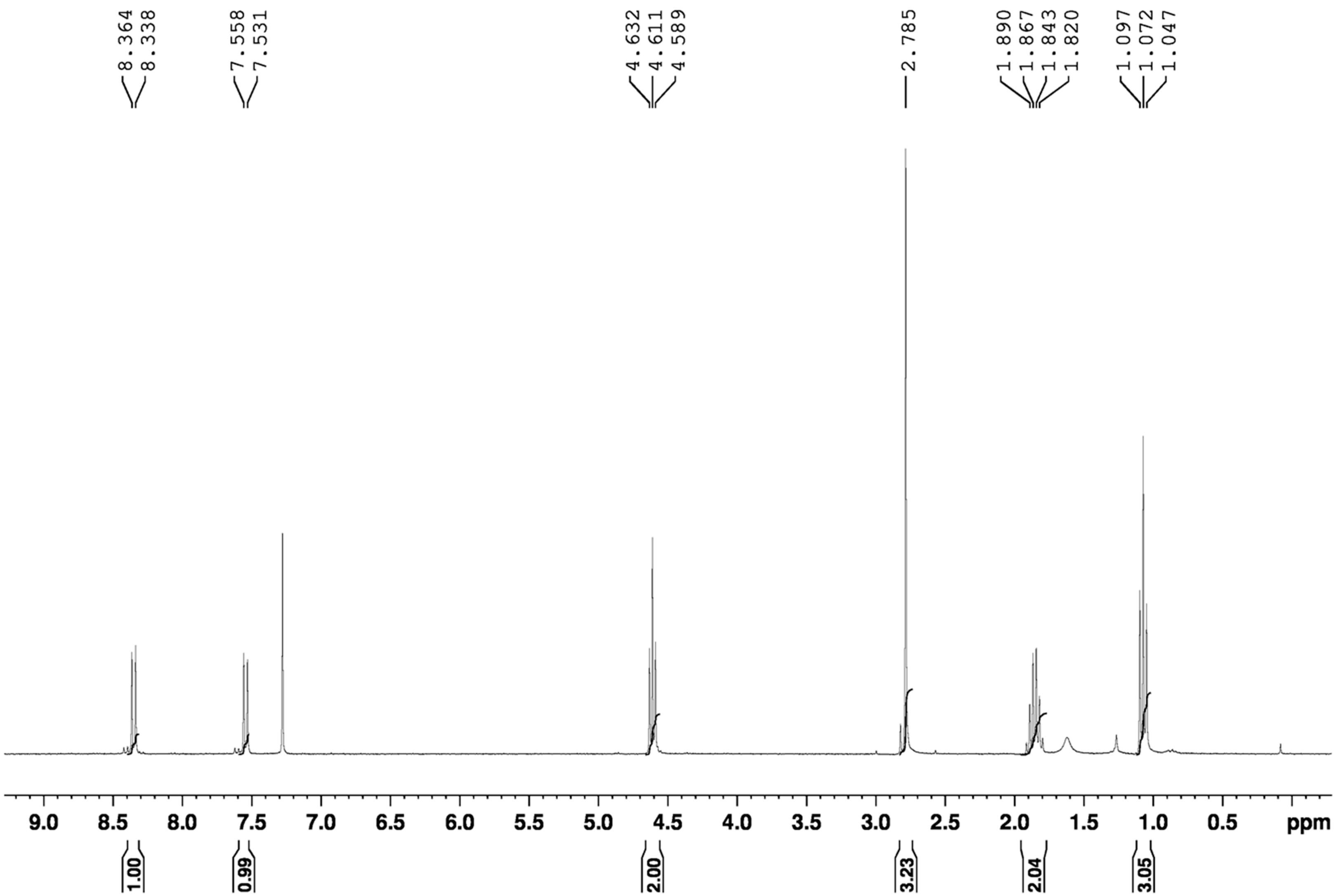

Figure S3 (a): ^{1}H-NMR spectrum of 6-chloro-2-methyl-7-propoxy-5,8-quinolinedione (**10**).

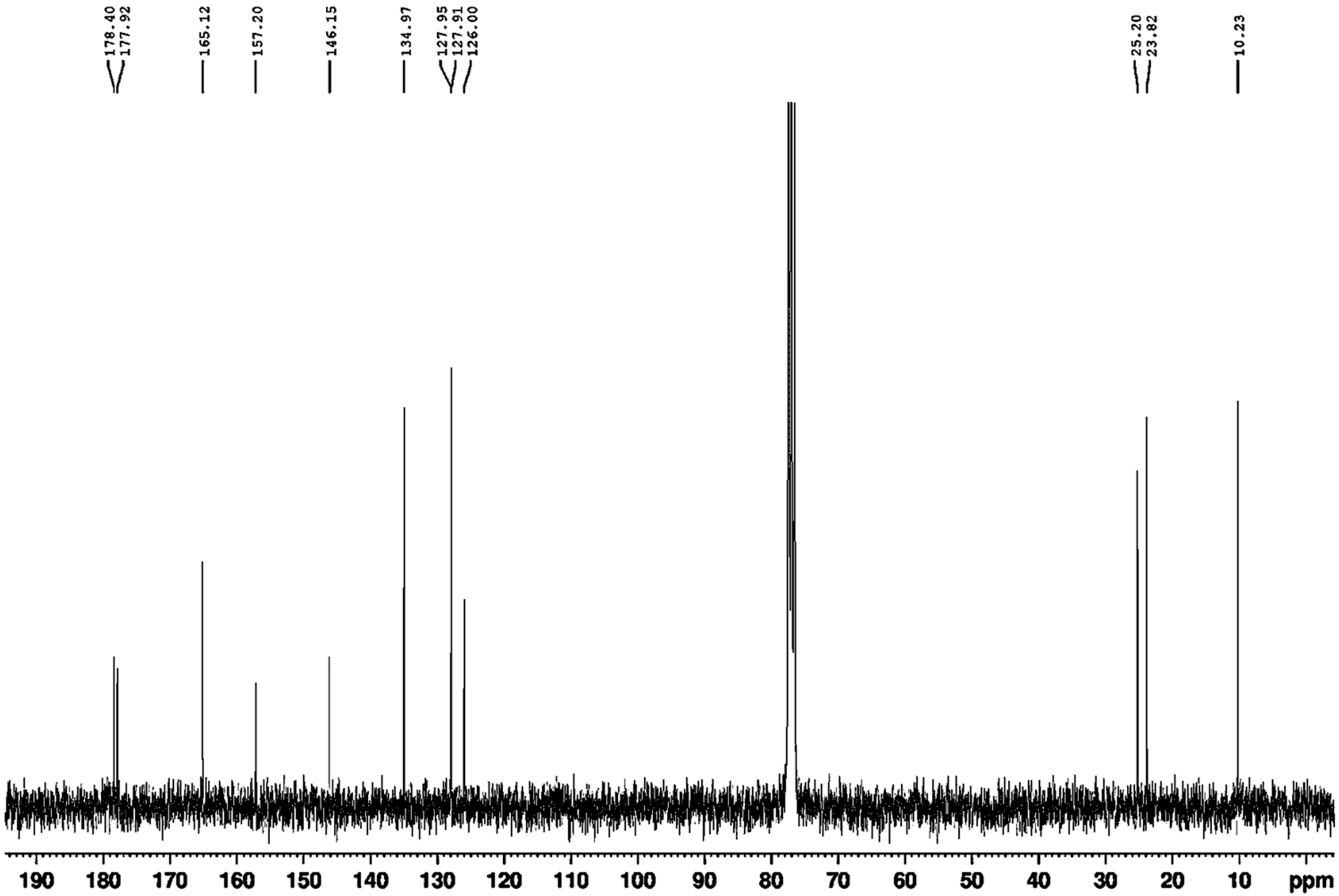

Figure S3 (b): ^{13}C-NMR spectrum of 6-chloro-2-methyl-7-propoxy-5,8-quinolinedione (**10**).

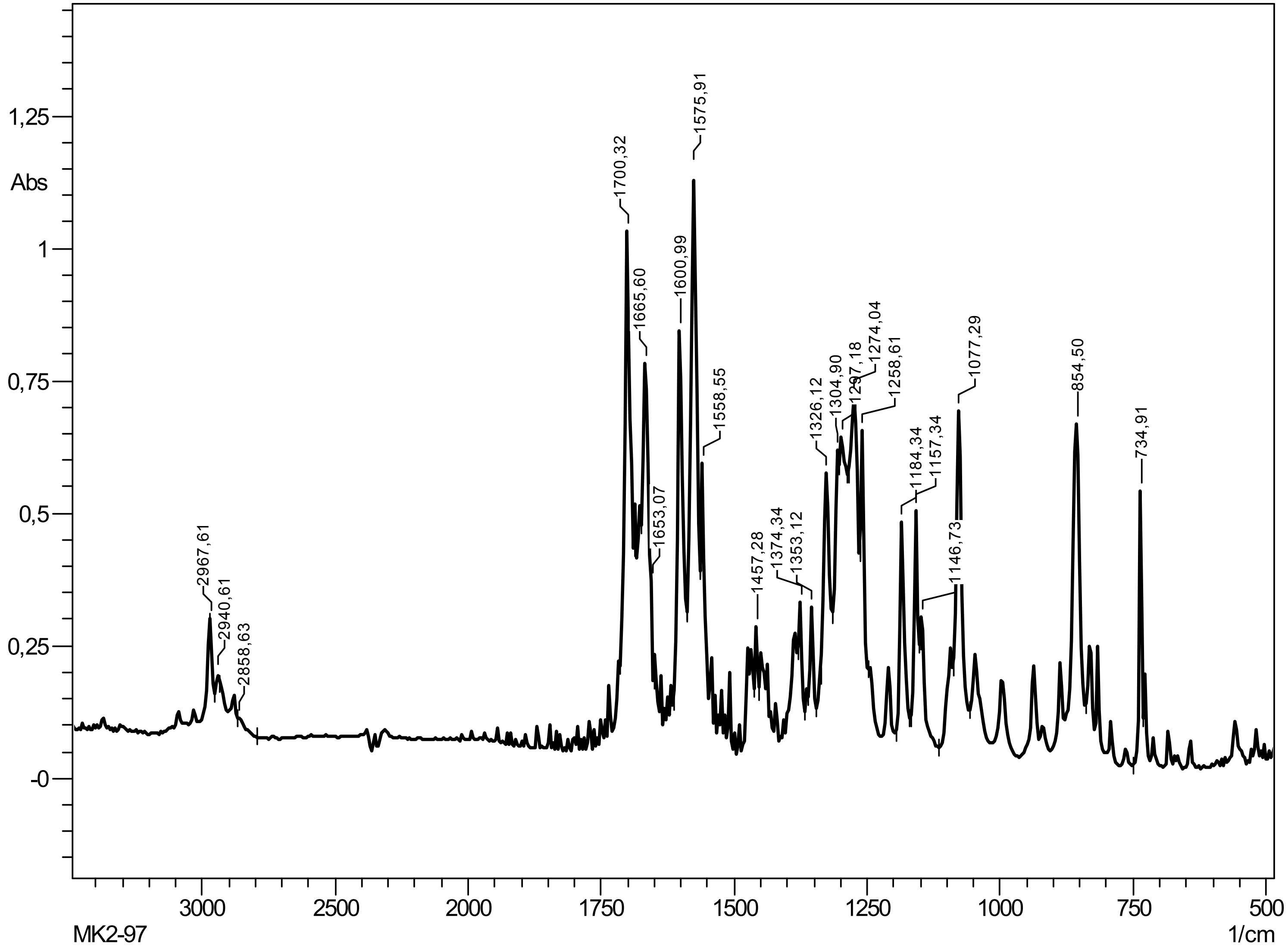

Figure S3 (c): IR spectrum of 6-chloro-2-methyl-7-propoxy-5,8-quinolinedione (**10**).

Compound Spectrum List Report

Analysis Info

Analysis Name	D:\Data\mk10.d	Acquisition Date	2/17/2017 11:28:53 AM	
Method	low_mass.m	Operator	KM	
Sample Name	TM Low concentration	Instrument	impact II	1825265.10082
Comment				

Acquisition Parameter

Source Type	ESI	Ion Polarity	Positive	Set Nebulizer	0.3 Bar
Focus	Active	Set Capillary	4000 V	Set Dry Heater	240 °C
Scan Begin	100 m/z	Set End Plate Offset	-500 V	Set Dry Gas	4.0 l/min
Scan End	1000 m/z	Set Charging Voltage	2000 V	Set Divert Valve	Source
		Set Corona	0 nA	Set APCI Heater	0 °C

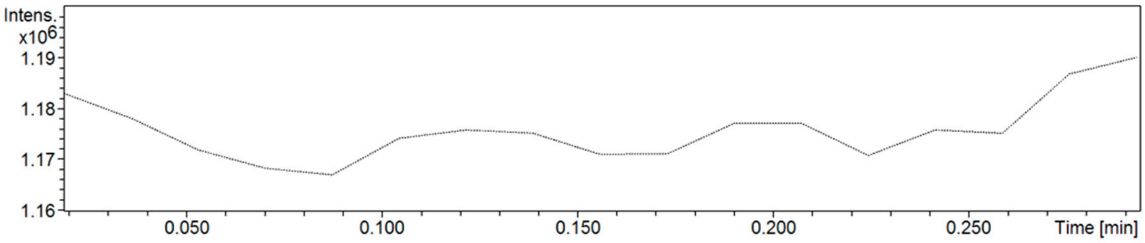

+MS, 0.0-0.3min #1-17

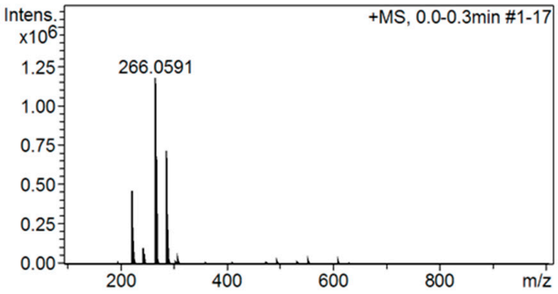

#	m/z	Res.	S/N	I	I %	FWHM
1	266.0591	31312	30530.3	1175831	100.0	0.0085

(d)

Figure S3 (d): HR-MS spectrum of 6-chloro-2-methyl-7-propoxy-5,8-quinolinedione (**10**).

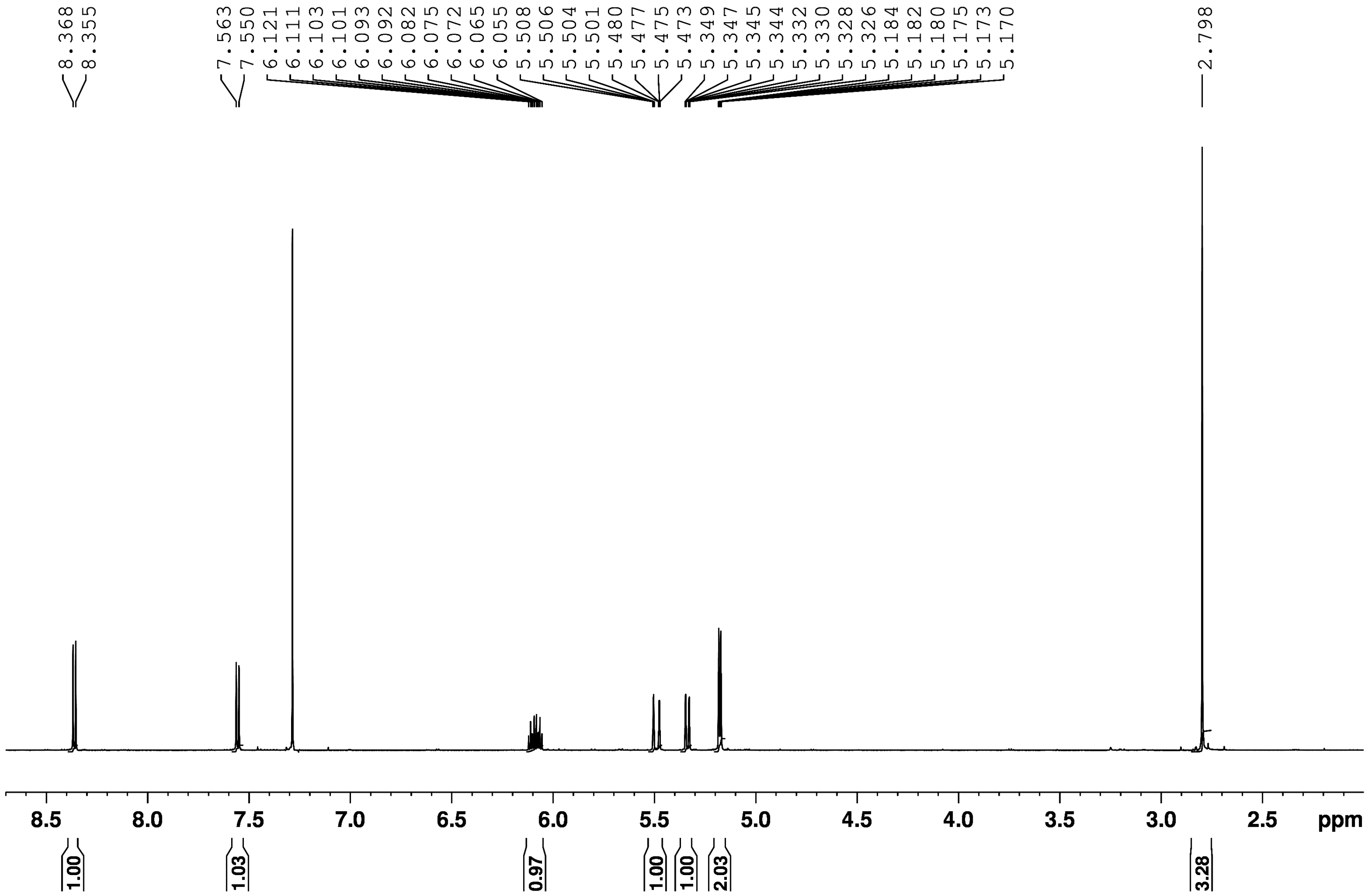

Figure S4 (a): ^{1}H-NMR spectrum of 6-chloro-2-methyl-7-(2-propenoxy)-5,8-quinolinedione (**11**).

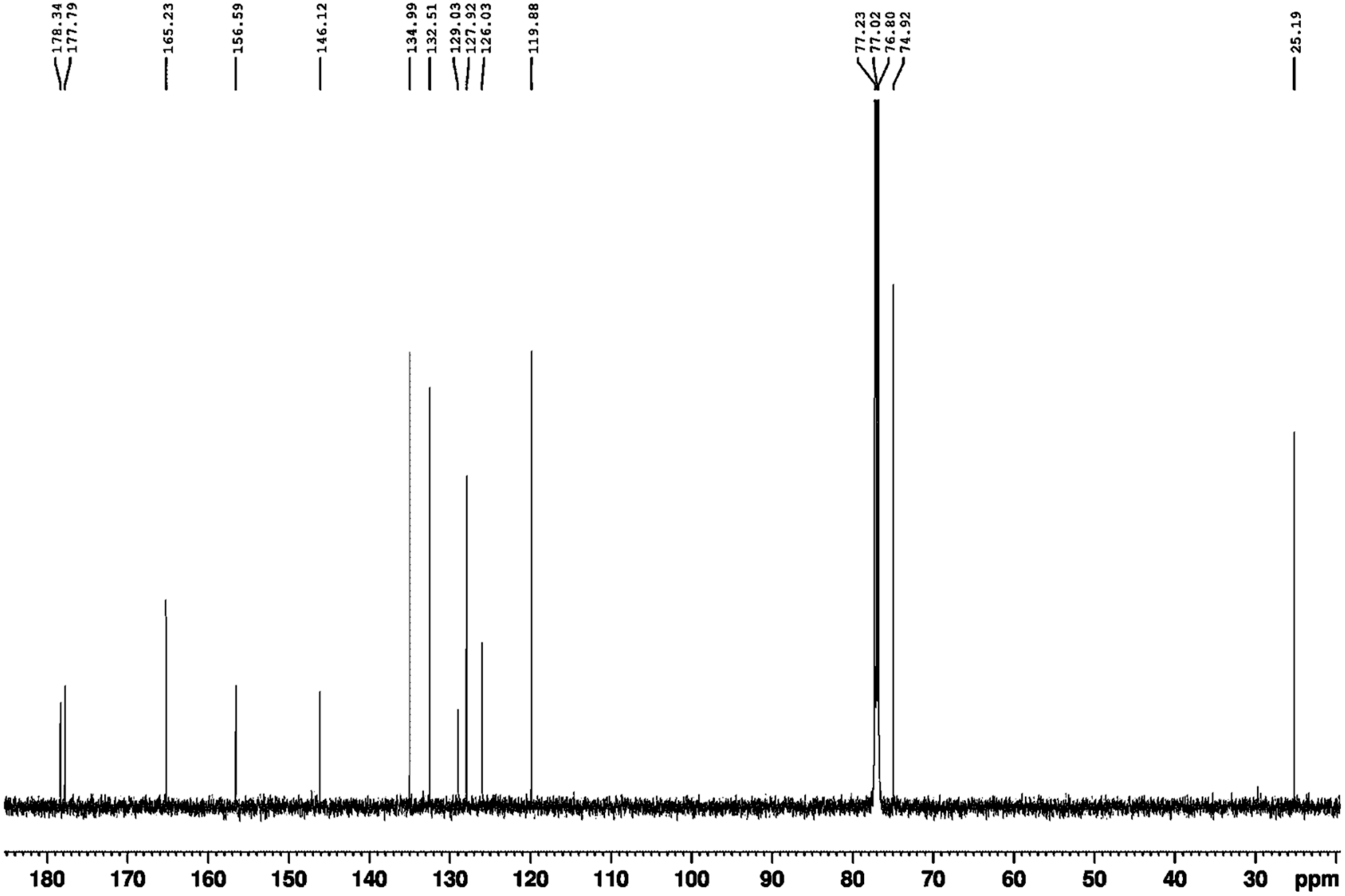

Figure S4 (b): ^{13}C-NMR spectrum of 6-chloro-2-methyl-7-(2-propenoxy)-5,8-quinolinedione (**11**).

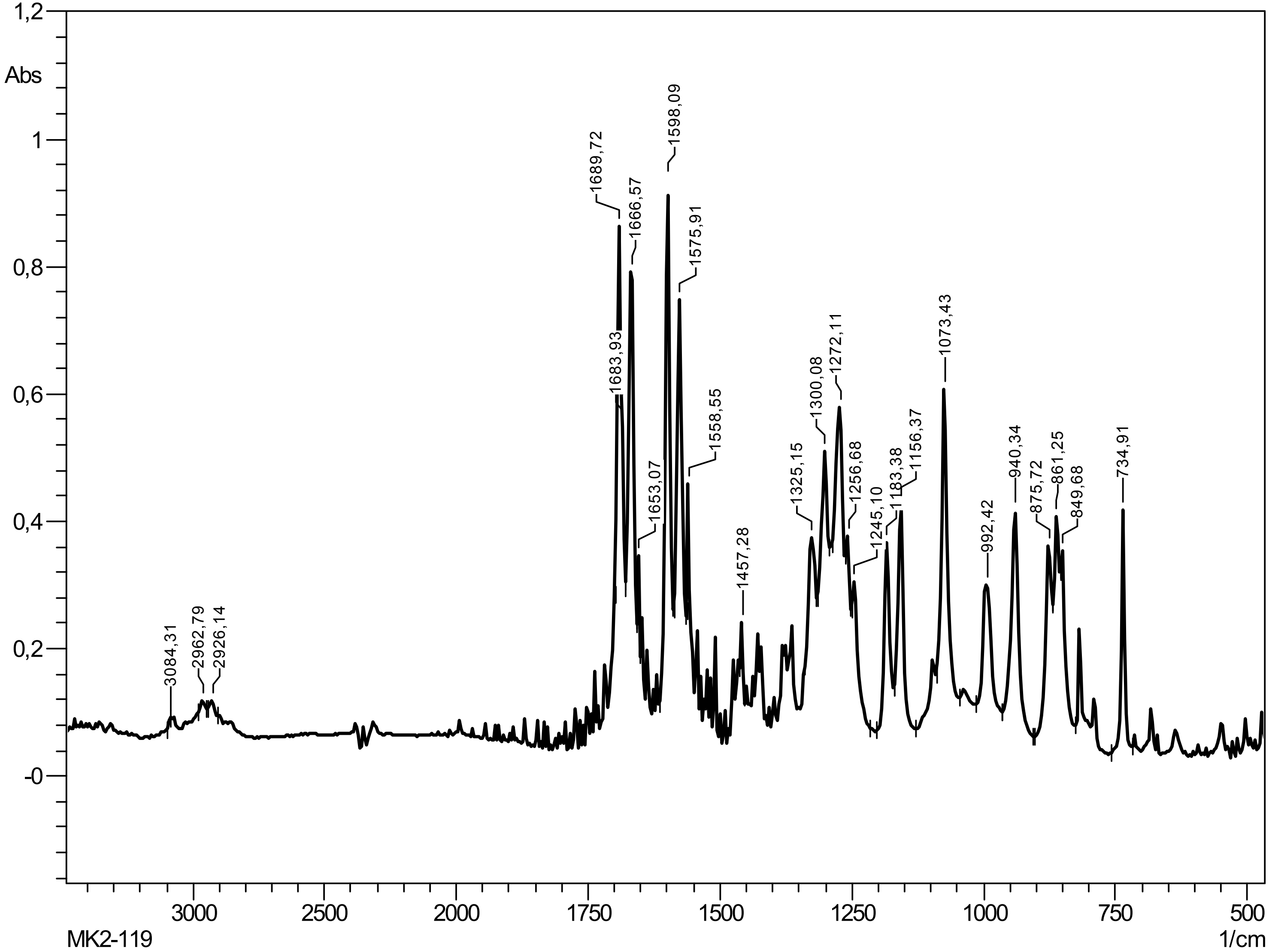

Figure S4 (c): IR spectrum of 6-chloro-2-methyl-7-(2-propenoxy)-5,8-quinolinedione (**11**).

Compound Spectrum List Report

Analysis Info

Analysis Name	D:\Data\mk11.d	Acquisition Date	2/17/2017 11:31:20 AM
Method	low_mass.m	Operator	KM
Sample Name	TM Low concentration	Instrument	impact II 1825265.10082
Comment			

Acquisition Parameter

Source Type	ESI	Ion Polarity	Positive	Set Nebulizer	0.3 Bar
Focus	Active	Set Capillary	4000 V	Set Dry Heater	240 °C
Scan Begin	100 m/z	Set End Plate Offset	-500 V	Set Dry Gas	4.0 l/min
Scan End	1000 m/z	Set Charging Voltage	2000 V	Set Divert Valve	Source
		Set Corona	0 nA	Set APCI Heater	0 °C

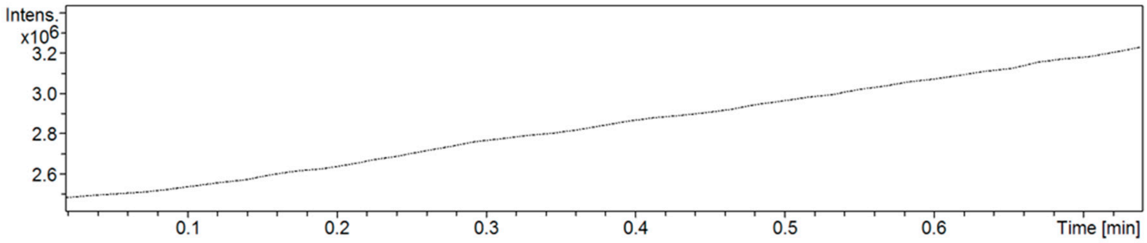

+MS, 0.0-0.7min #1-43

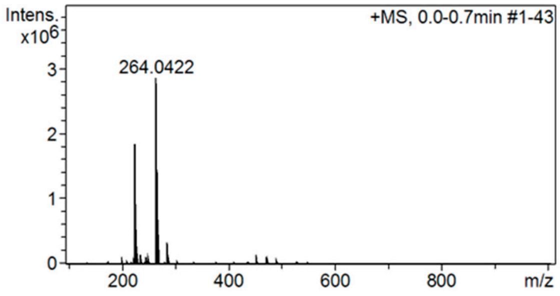

#	m/z	Res.	S/N	I	I %	FWHM
1	225.0186	32453	42983.7	1844651	64.6	0.0069
2	264.0422	35688	49878.3	2857194	100.0	0.0074

Figure S4 (d): HR-MS spectrum of 6-chloro-2-methyl-7-(2-propenoxy)-5,8-quinolinedione (**11**).

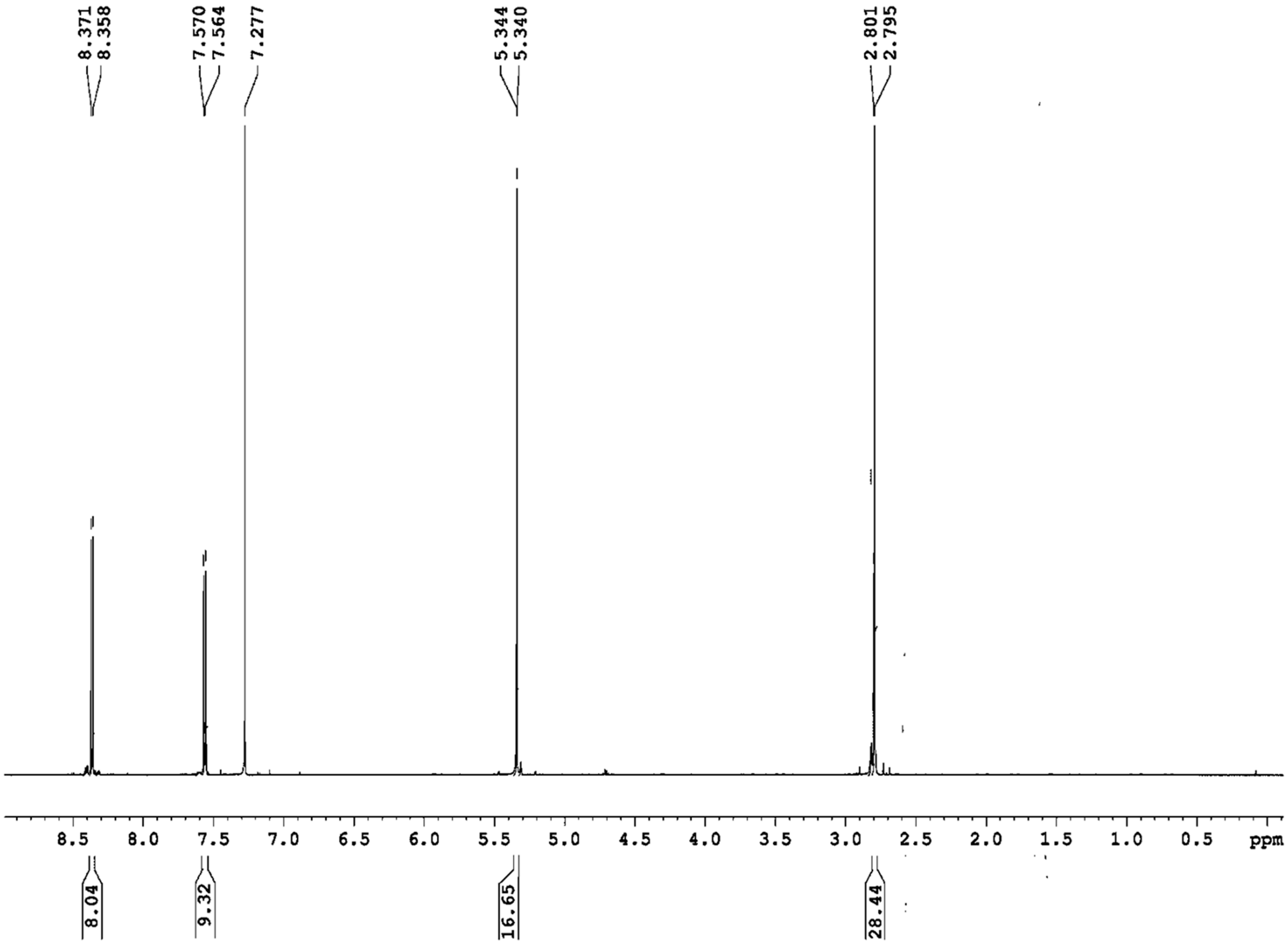

Figure S5 (a): ^{1}H-NMR spectrum of 6-chloro-2-methyl-7-(2-propynoxy)-5,8-quinolinedione (**12**).

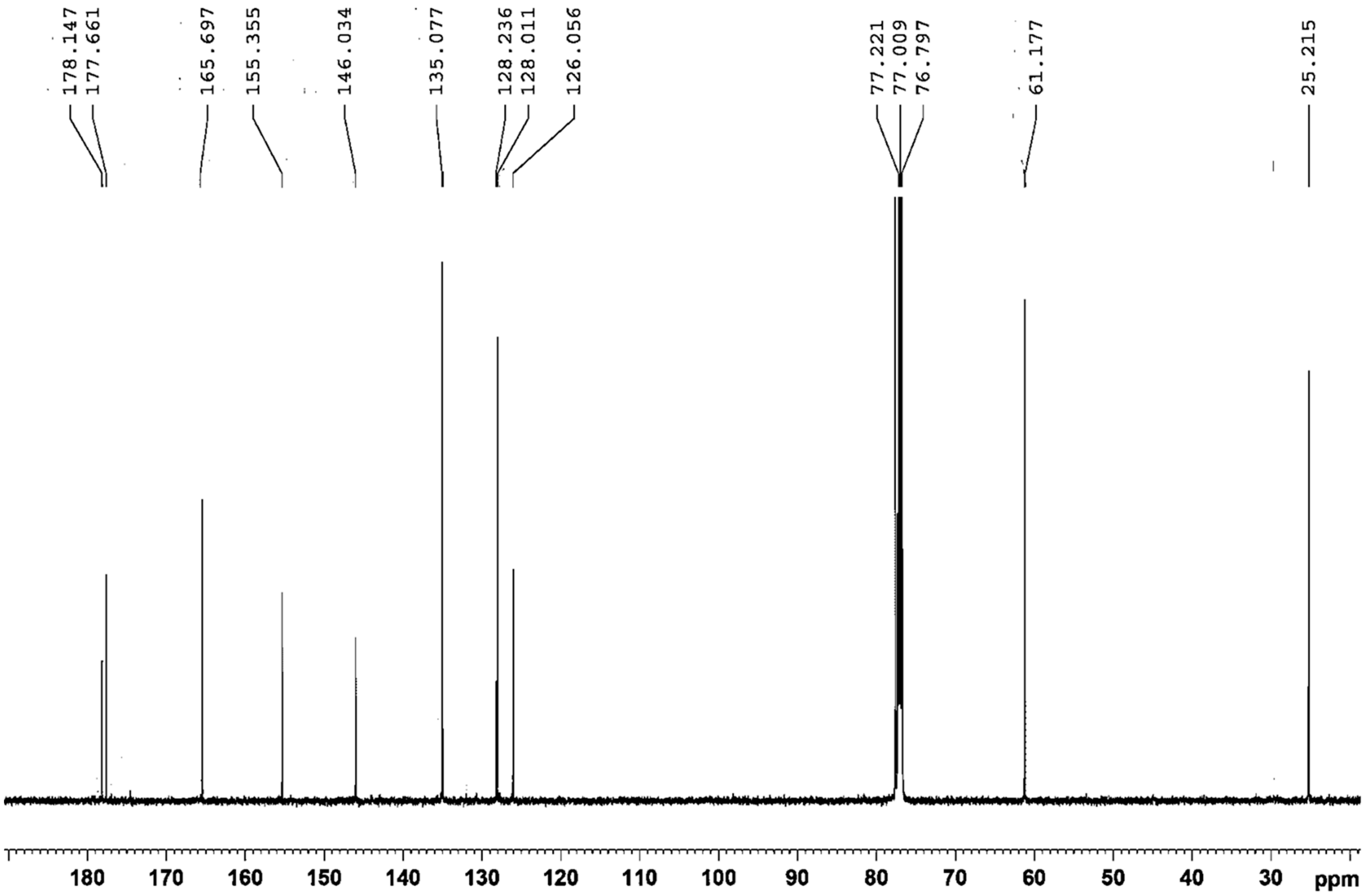

Figure S5 (b): ^{13}C-NMR spectrum of 6-chloro-2-methyl-7-(2-propynoxy)-5,8-quinolinedione (**12**).

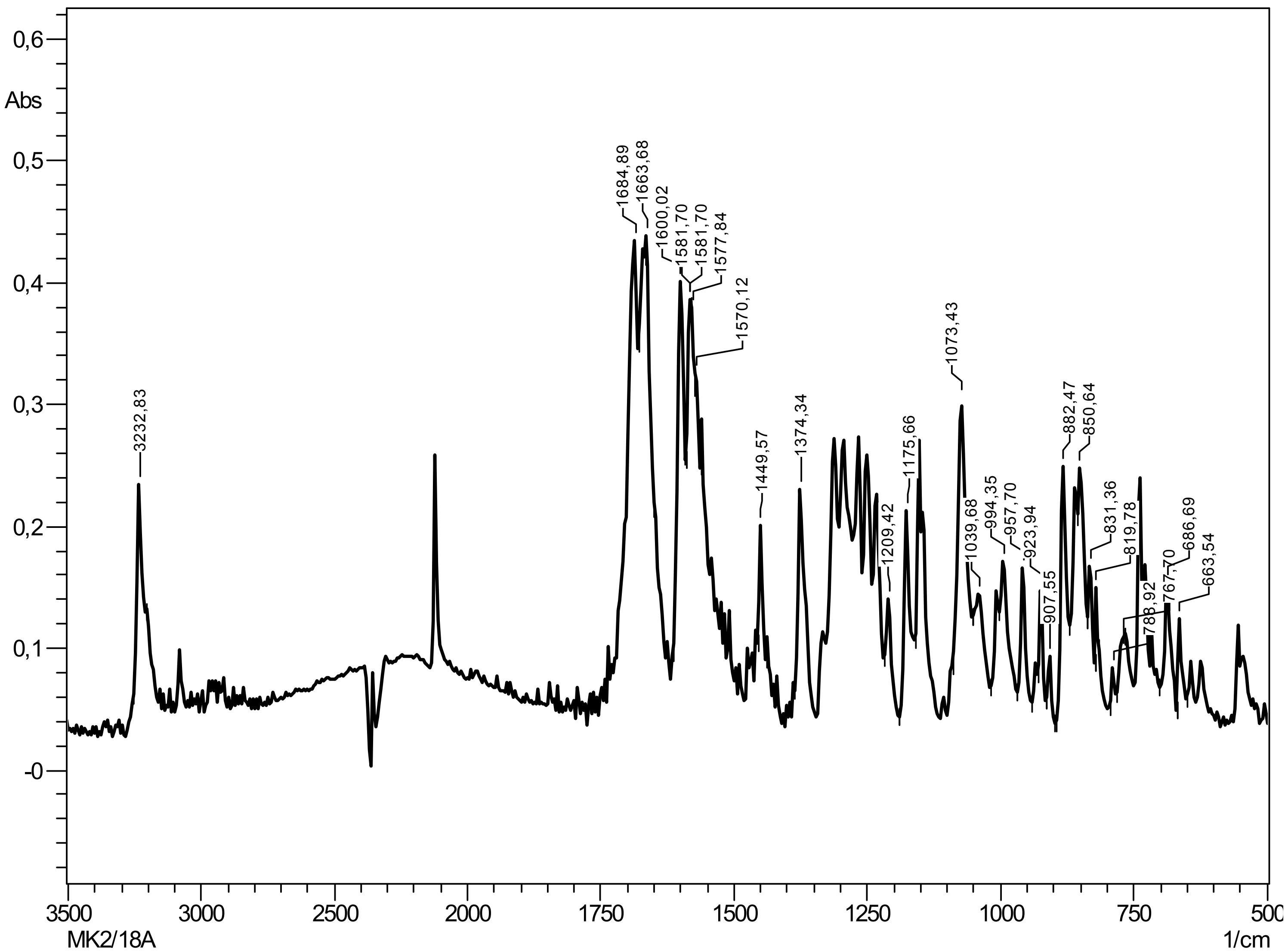

Figure S5 (c): IR spectrum of 6-chloro-2-methyl-7-(2-propynoxy)-5,8-quinolinedione (**12**).

Compound Spectrum List Report

Analysis Info

Analysis Name	D:\Data\mk12.d	Acquisition Date	2/17/2017 11:33:58 AM
Method	low_mass.m	Operator	KM
Sample Name	TM Low concentration	Instrument	impact II 1825265.10082
Comment			

Acquisition Parameter

Source Type	ESI	Ion Polarity	Positive	Set Nebulizer	0.3 Bar
Focus	Active	Set Capillary	4000 V	Set Dry Heater	240 °C
Scan Begin	100 m/z	Set End Plate Offset	-500 V	Set Dry Gas	4.0 l/min
Scan End	1000 m/z	Set Charging Voltage	2000 V	Set Divert Valve	Source
		Set Corona	0 nA	Set APCI Heater	0 °C

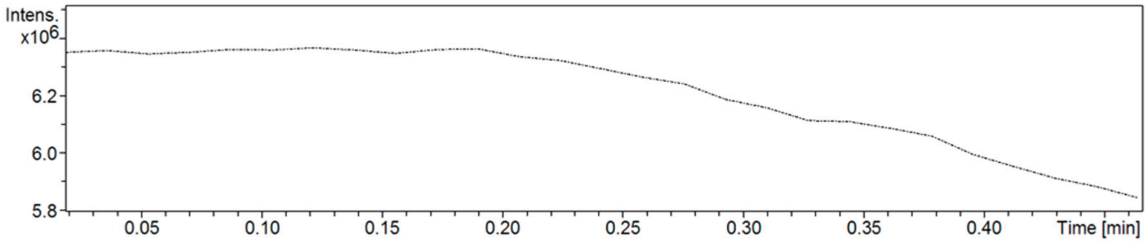

+MS, 0.0-0.5min #1-27

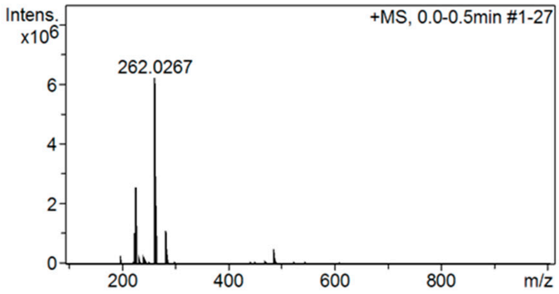

#	m/z	Res.	S/N	I	I %	FWHM
1	227.0576	33332	41660.4	2579390	41.5	0.0068
2	262.0267	37503	86017.8	6213385	100.0	0.0070

Figure S5 (d): HR-MS spectrum of 6-chloro-2-methyl-7-(2-propynoxy)-5,8-quinolinedione (**12**).

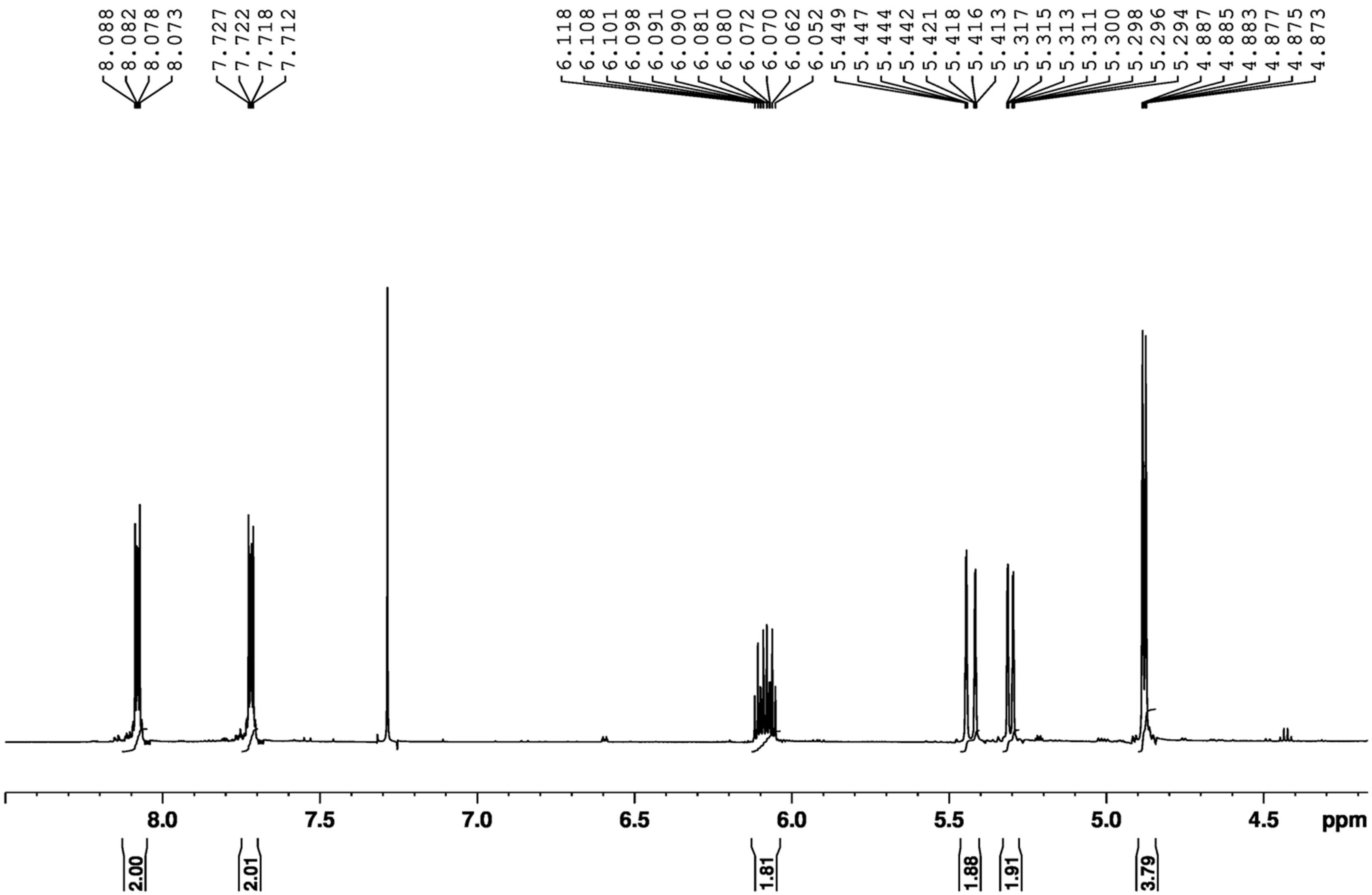

Figure S6 (a): ^{13}C-NMR spectrum of 2,3-di(2-propenoxy)-1,4-naphtoquinon (**14**).

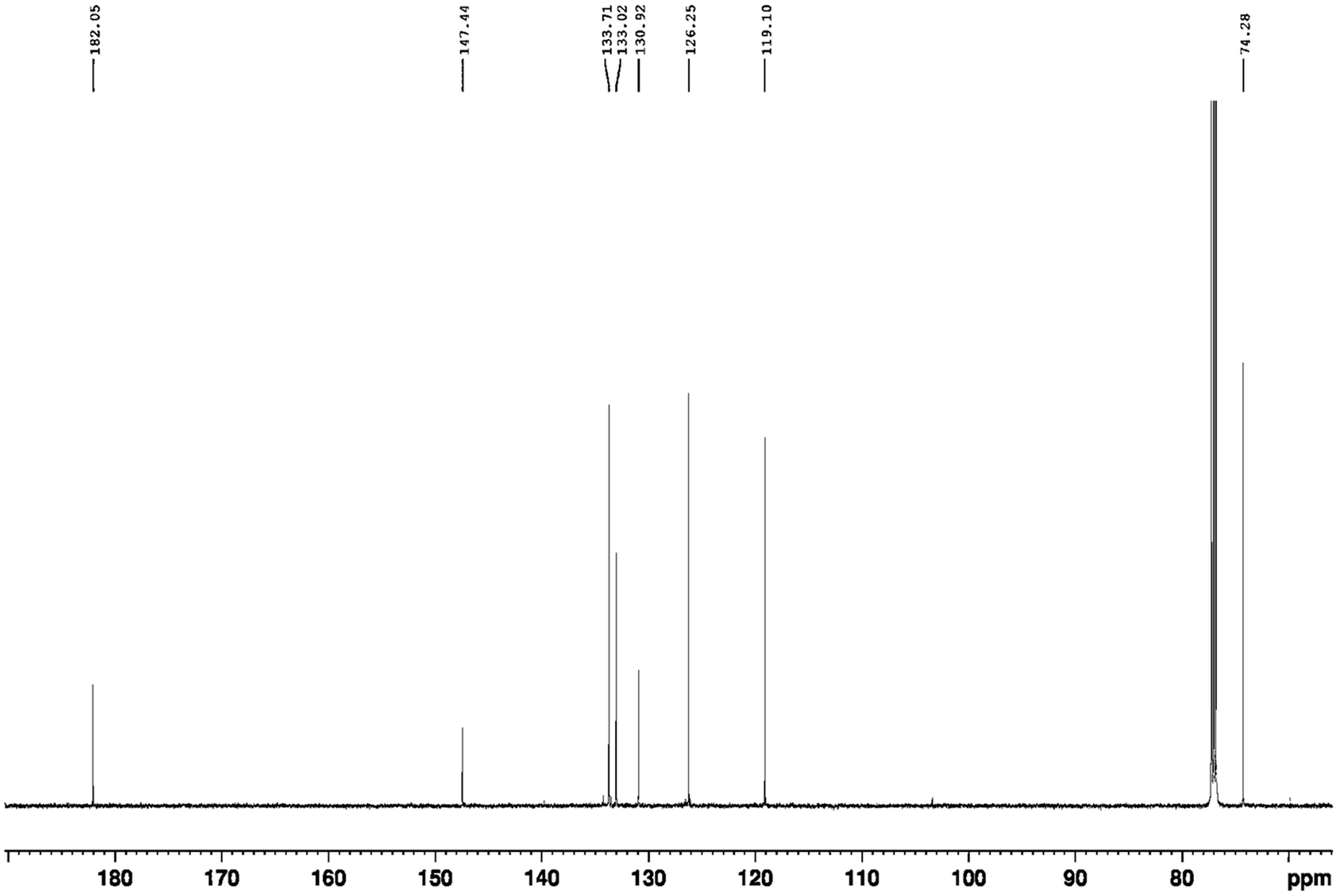

Figure S6 (b): ^{1}H-NMR spectrum of 2,3-di(2-propenoxy)-1,4-naphtoquinon (**14**).

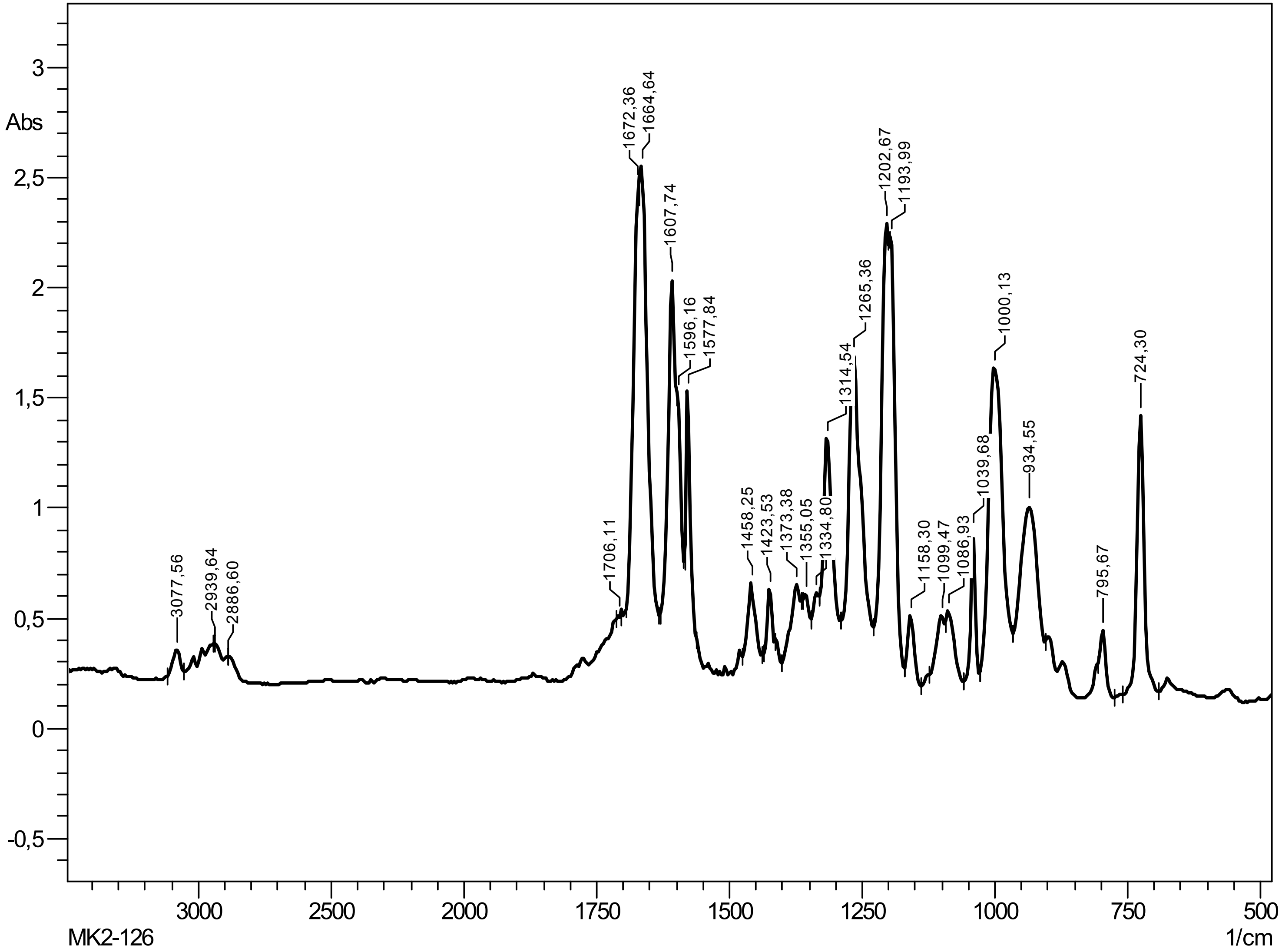

Figure S6 (c): IR spectrum of 2,3-di(2-propenoxy)-1,4-naphtoquinon (**14**).

Compound Spectrum List Report

Analysis Info

Analysis Name	D:\Data\mk14b.d	Acquisition Date	2/17/2017 12:11:12 PM	
Method	low_mass.m	Operator	KM	
Sample Name	TM Low concentration	Instrument	impact II	1825265.10082
Comment				

Acquisition Parameter

Source Type	ESI	Ion Polarity	Positive	Set Nebulizer	0.3 Bar
Focus	Active	Set Capillary	4000 V	Set Dry Heater	240 °C
Scan Begin	100 m/z	Set End Plate Offset	-500 V	Set Dry Gas	4.0 l/min
Scan End	1000 m/z	Set Charging Voltage	2000 V	Set Divert Valve	Source
		Set Corona	0 nA	Set APCI Heater	0 °C

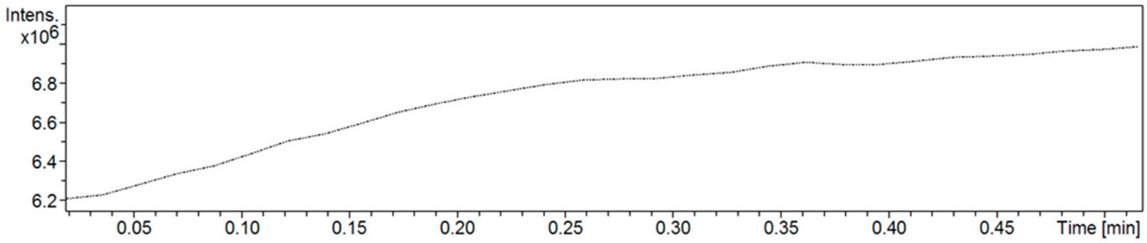

+MS, 0.0-0.5min #1-30

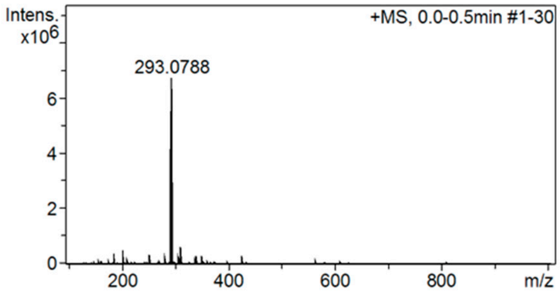

#	m/z	Res.	S/N	I	I %	FWHM
1	293.0788	39470	44707.4	6722752	100.0	0.0074

Figure S6 (d): HR-MS spectrum of 2,3-di(2-propenoxy)-1,4-naphtoquinon (**14**).

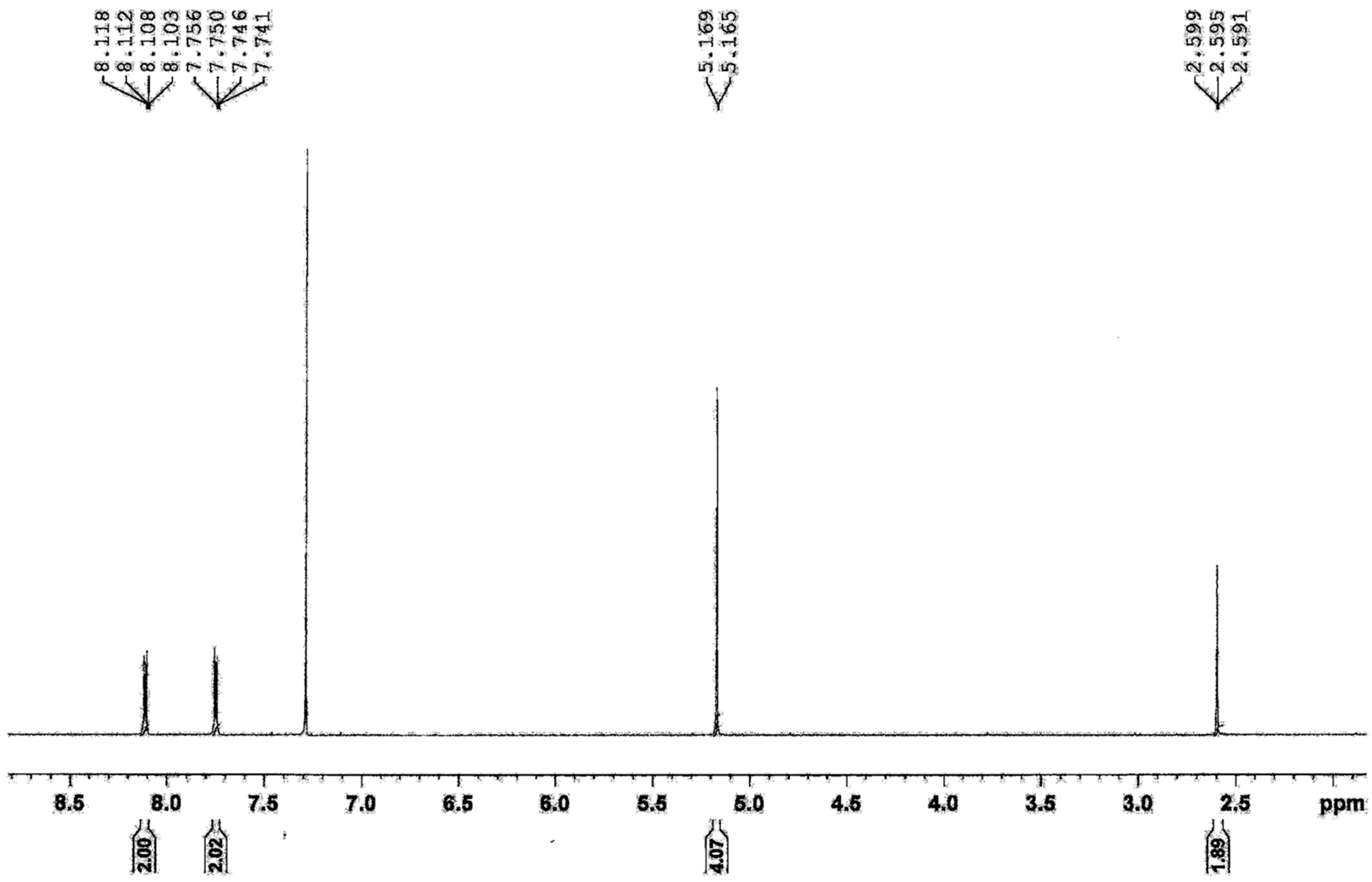

Figure S7 (a): ^{1}H-NMR spectrum of 2,3-di(2-propynoxy)-1,4-naphthoquinon (**15**).

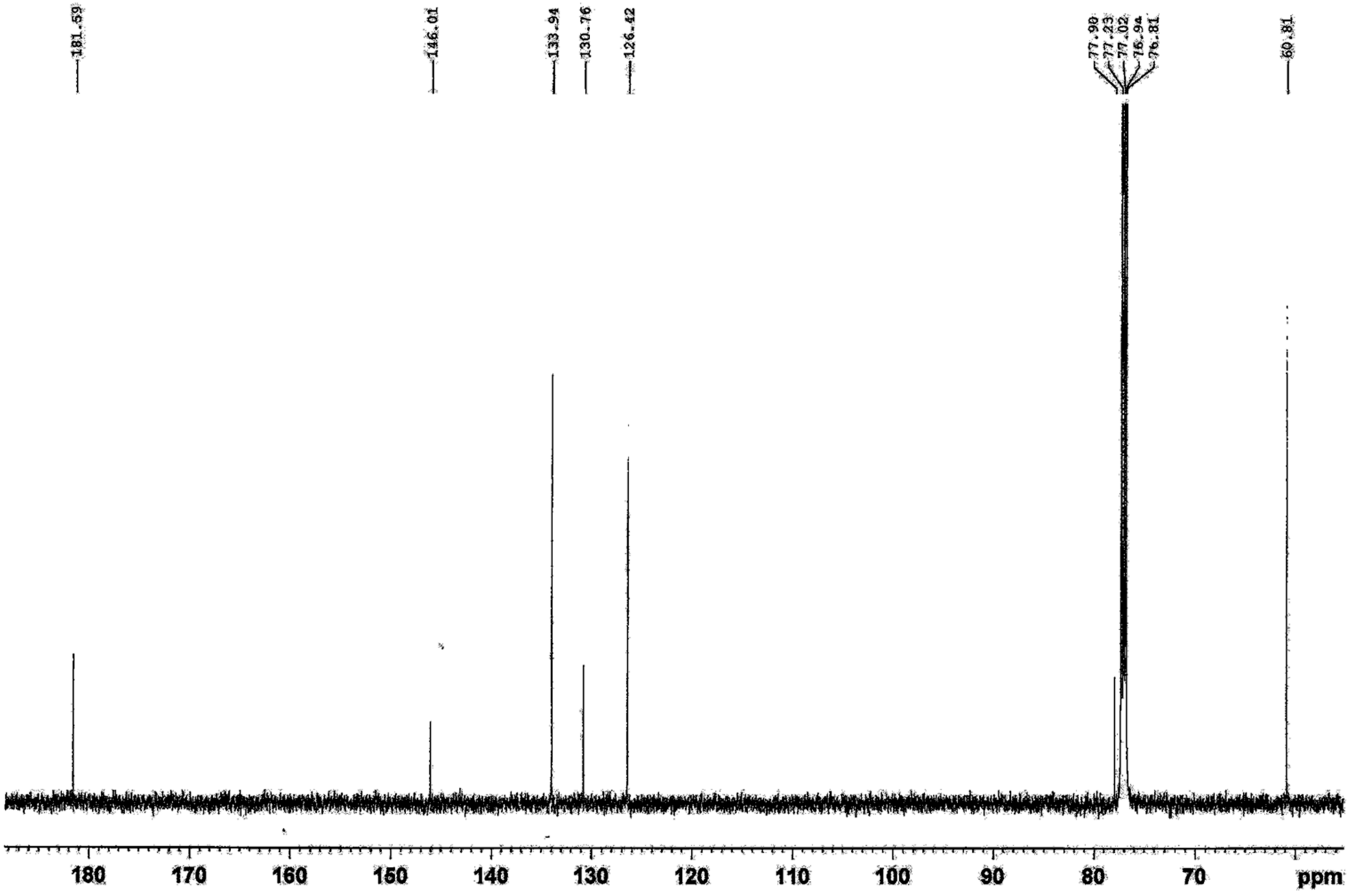

Figure S7 (b): ^{13}C-NMR spectrum of 2,3-di(2-propynoxy)-1,4-naphthoquinon (**15**).

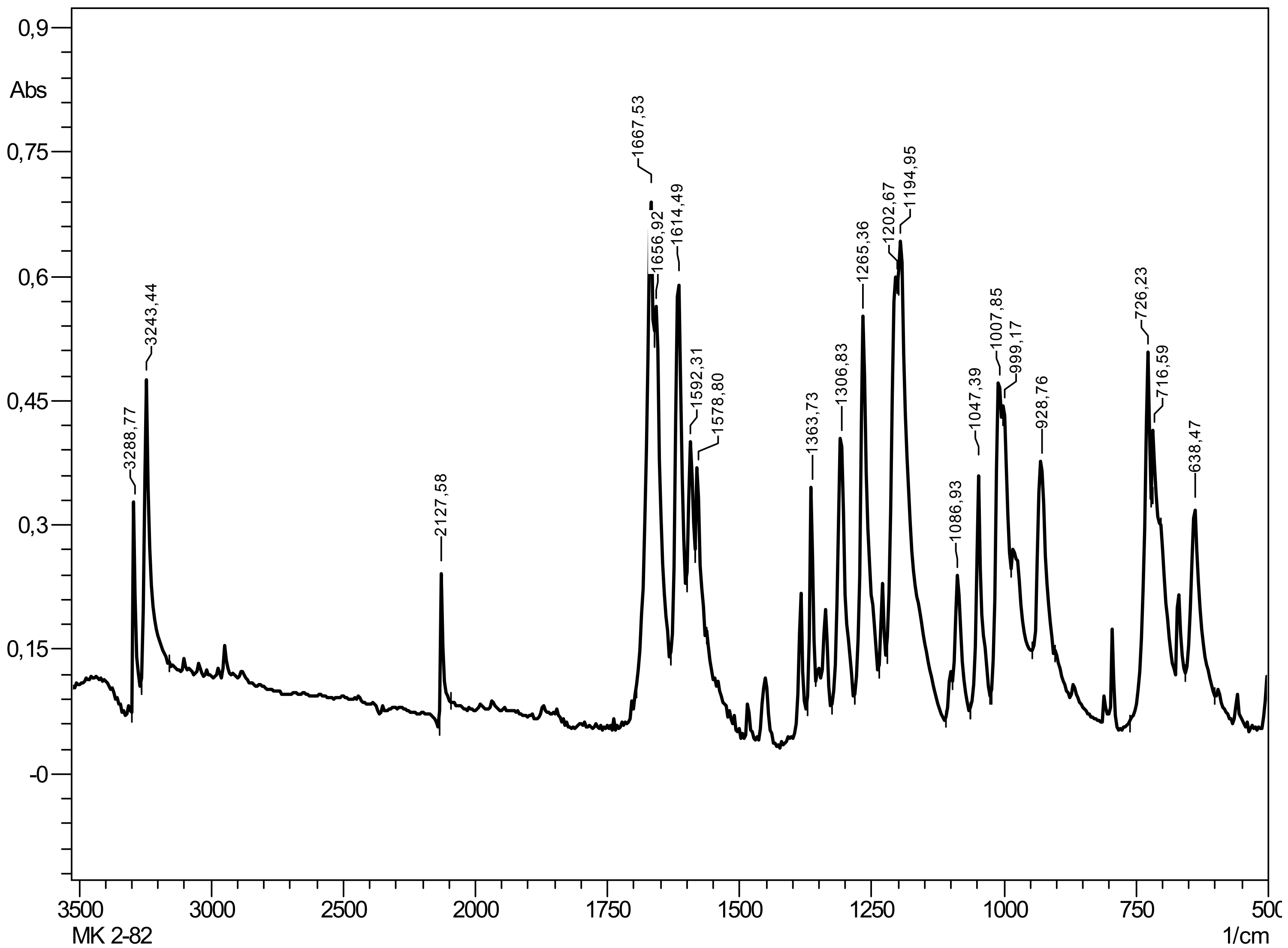

Figure S7 (c): IR spectrum of 2,3-di(2-propynoxy)-1,4-naphthoquinon (**15**).

Compound Spectrum List Report

Analysis Info

Analysis Name	D:\Data\mk15b.d	Acquisition Date	2/17/2017 12:18:02 PM
Method	low_mass.m	Operator	KM
Sample Name	TM Low concentration	Instrument	impact II 1825265.10082
Comment			

Acquisition Parameter

Source Type	ESI	Ion Polarity	Positive	Set Nebulizer	0.3 Bar
Focus	Active	Set Capillary	4000 V	Set Dry Heater	240 °C
Scan Begin	100 m/z	Set End Plate Offset	-500 V	Set Dry Gas	4.0 l/min
Scan End	1000 m/z	Set Charging Voltage	2000 V	Set Divert Valve	Source
		Set Corona	0 nA	Set APCI Heater	0 °C

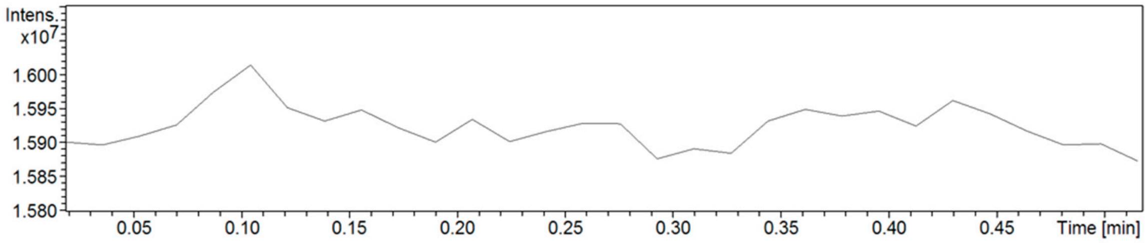

+MS, 0.0-0.5min #1-30

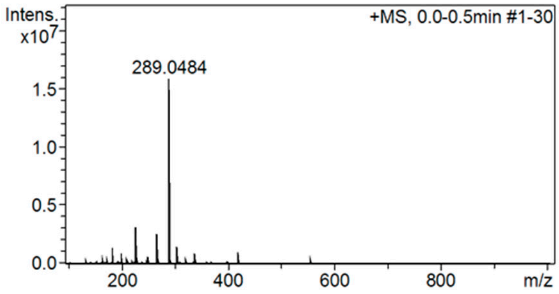

#	m/z	Res.	S/N	I	I %	FWHM
1	289.0484	38736	59031.0	15912883	100.0	0.0075

Figure S7 (d): HR-MS spectrum of 2,3-di(2-propynoxy)-1,4-naphthoquinon (**15**).

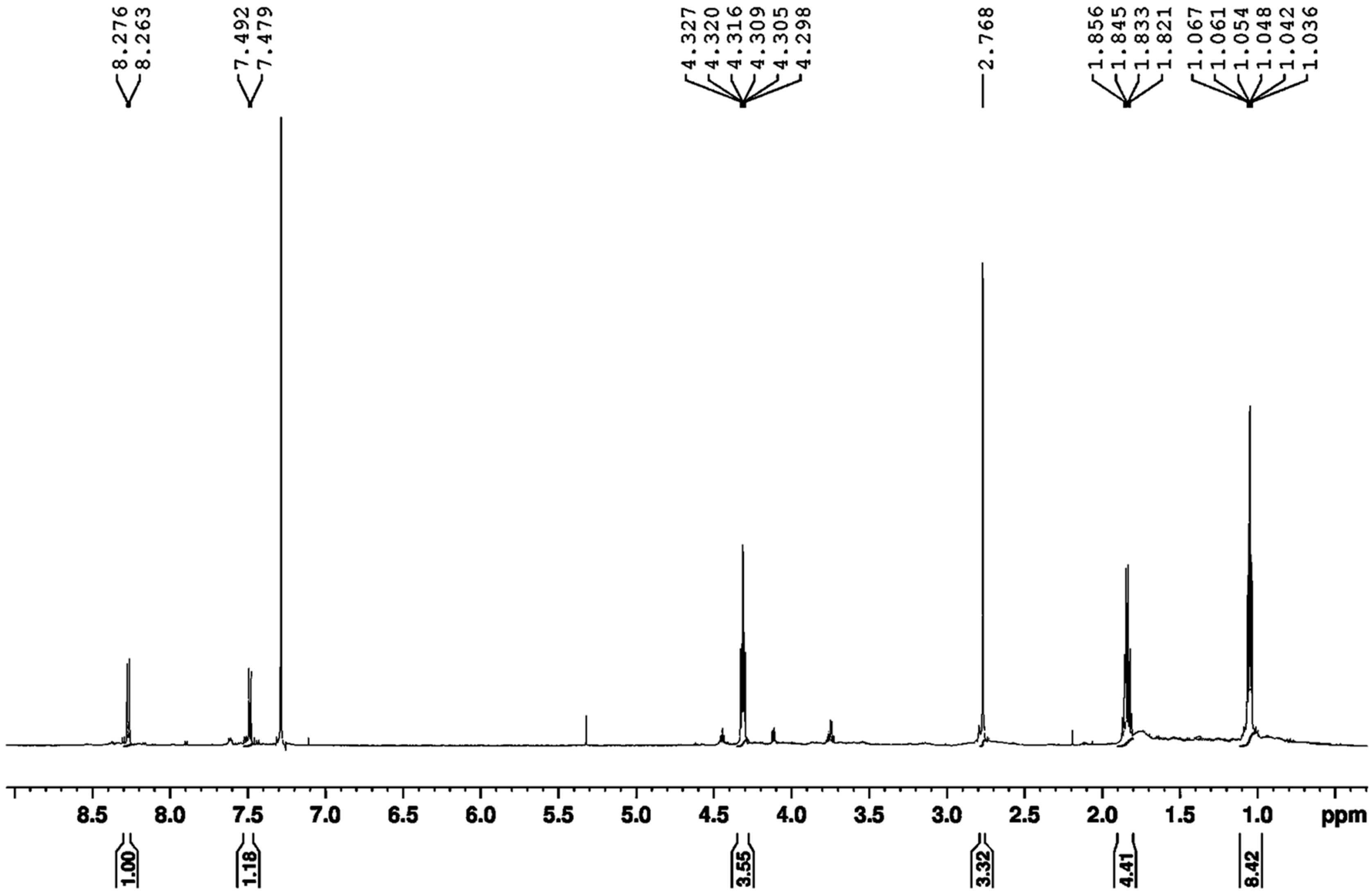

Figure S8 (a): ^{1}H-NMR spectrum of 2-methyl-6,7-dipropoxy-5,8-quinolinedione (**19**).

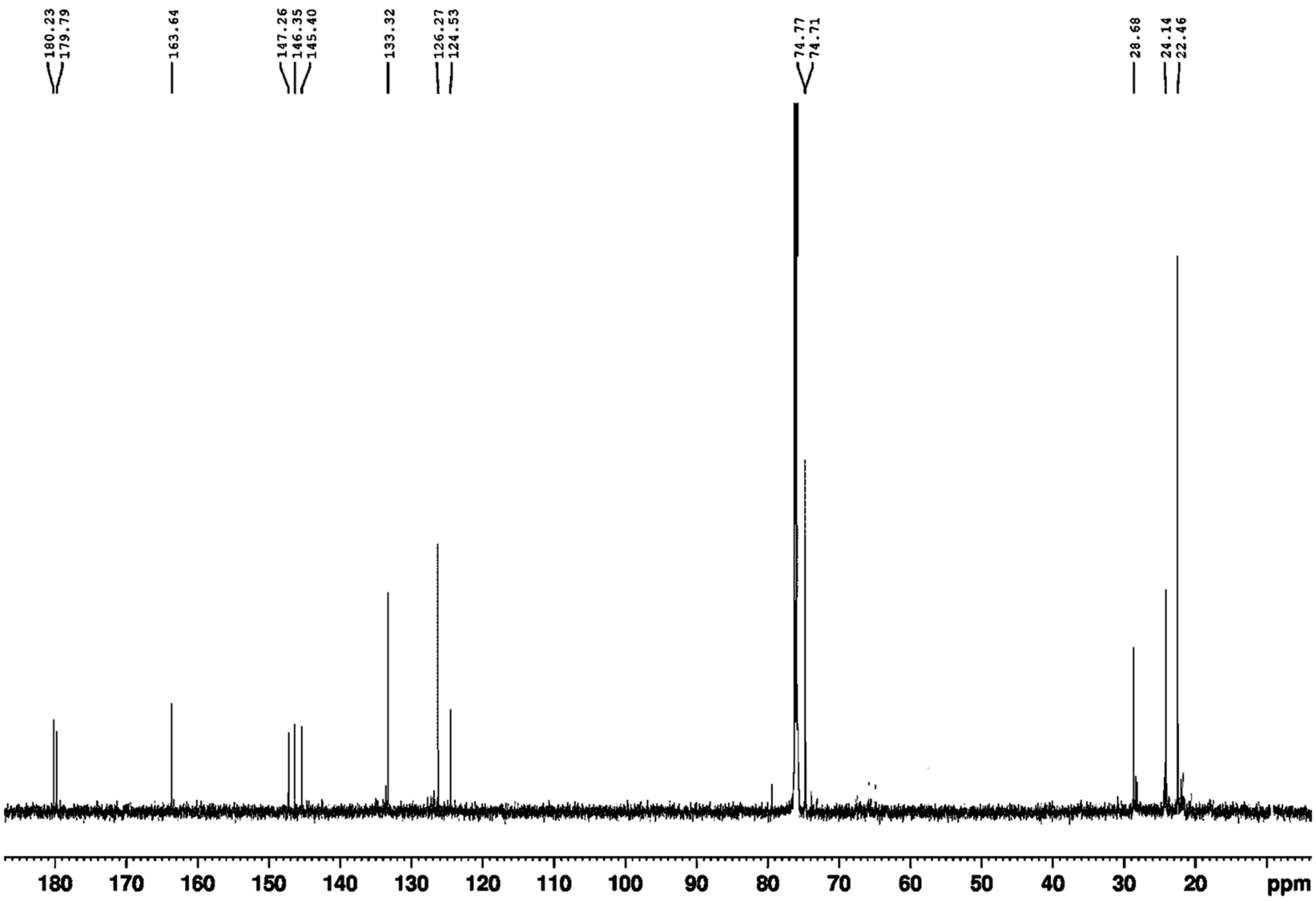

Figure S8 (b): ^{13}C-NMR spectrum of 2-methyl-6,7-dipropoxy-5,8-quinolinedione (**19**).

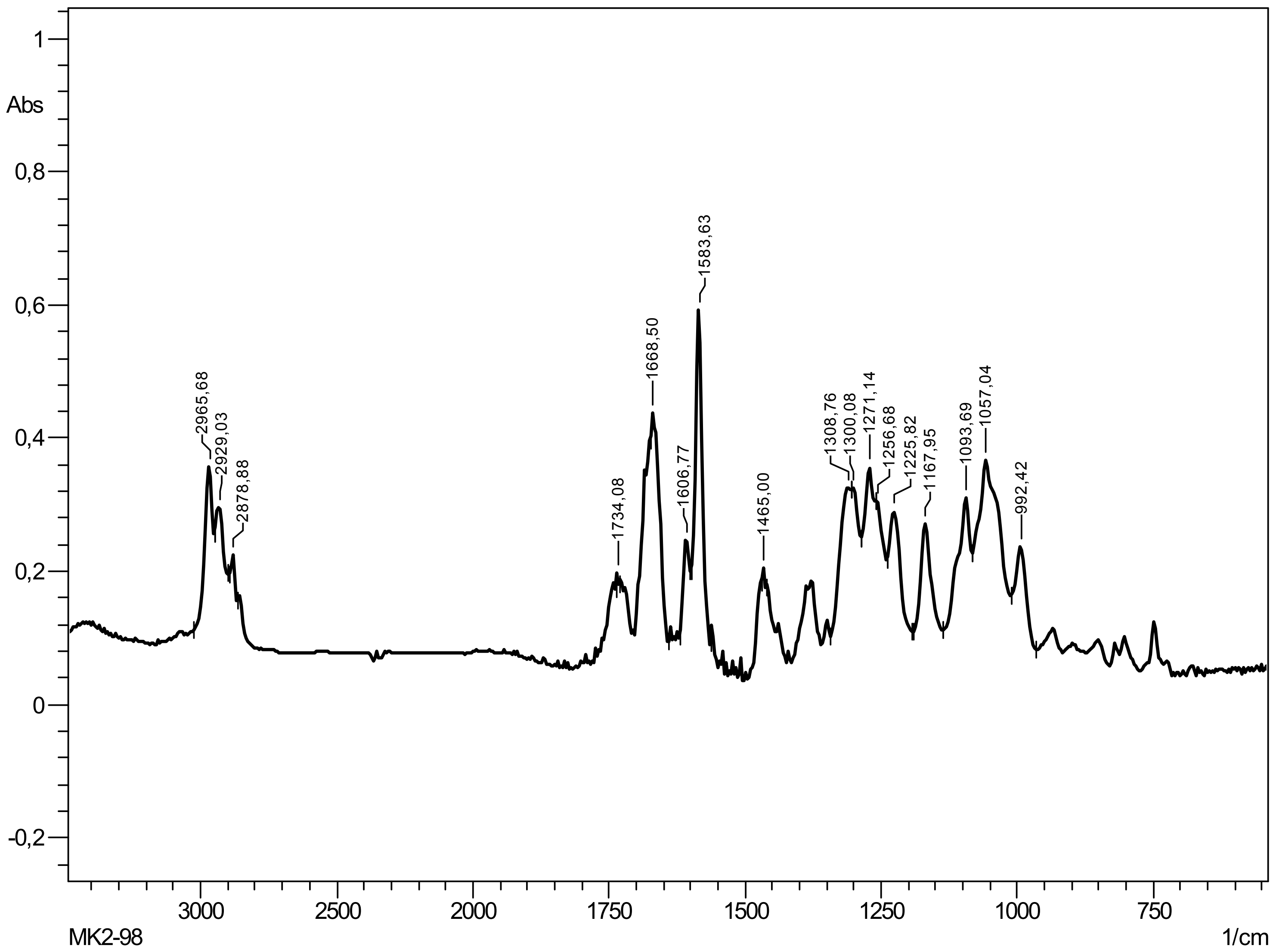

Figure S8 (c): IR spectrum of 2-methyl-6,7-dipropoxy-5,8-quinolinedione (**19**).

Compound Spectrum List Report

Analysis Info

Analysis Name	D:\Data\mk19.d	Acquisition Date	2/17/2017 11:46:11 AM
Method	low_mass.m	Operator	KM
Sample Name	TM Low concentration	Instrument	impact II 1825265.10082
Comment			

Acquisition Parameter

Source Type	ESI	Ion Polarity	Positive	Set Nebulizer	0.3 Bar
Focus	Active	Set Capillary	4000 V	Set Dry Heater	240 °C
Scan Begin	100 m/z	Set End Plate Offset	-500 V	Set Dry Gas	4.0 l/min
Scan End	1000 m/z	Set Charging Voltage	2000 V	Set Divert Valve	Source
		Set Corona	0 nA	Set APCI Heater	0 °C

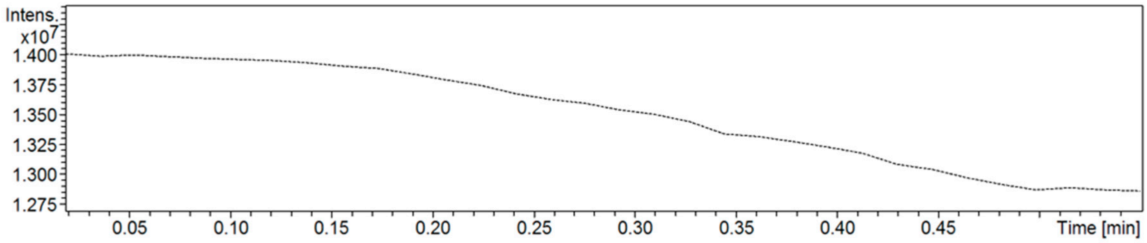

+MS, 0.0-0.5min #1-32

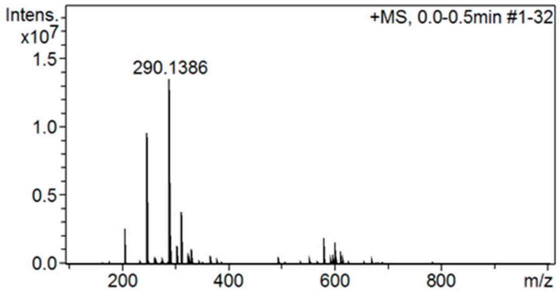

#	m/z	Res.	S/N	I	I %	FWHM
1	206.0444	33147	27518.0	2659770	19.7	0.0062
2	248.0915	36233	75963.1	9535350	70.5	0.0068
3	290.1386	38892	73922.7	13516469	100.0	0.0075

Figure S8 (d): HR-MS spectrum of 2-methyl-6,7-dipropoxy-5,8-quinolinedione (**19**).

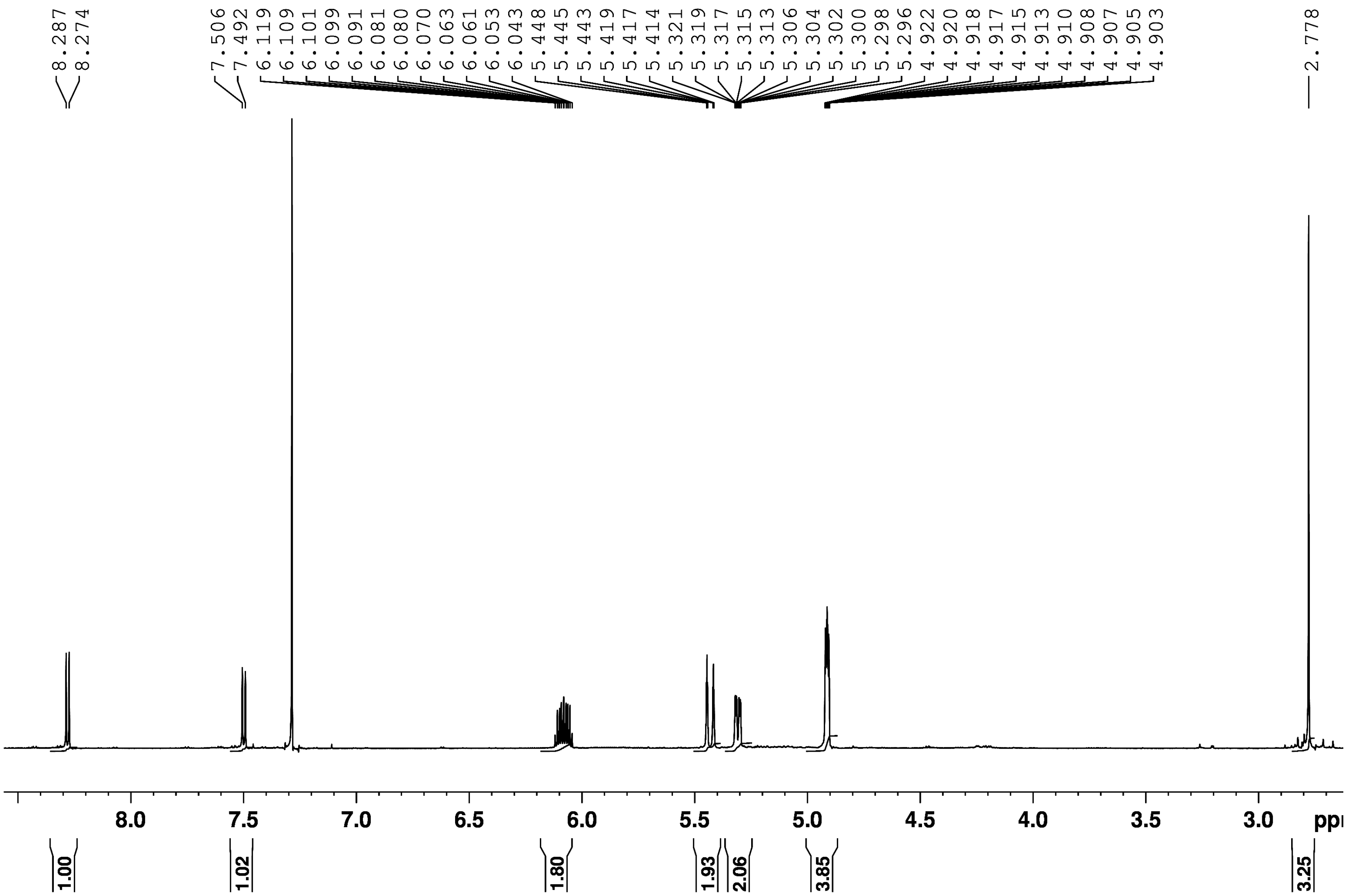

Figure S9 (a): ^{1}H-NMR spectrum of 2-methyl-6,7-di(2-propenoxy)-5,8-quinolinedione (**20**).

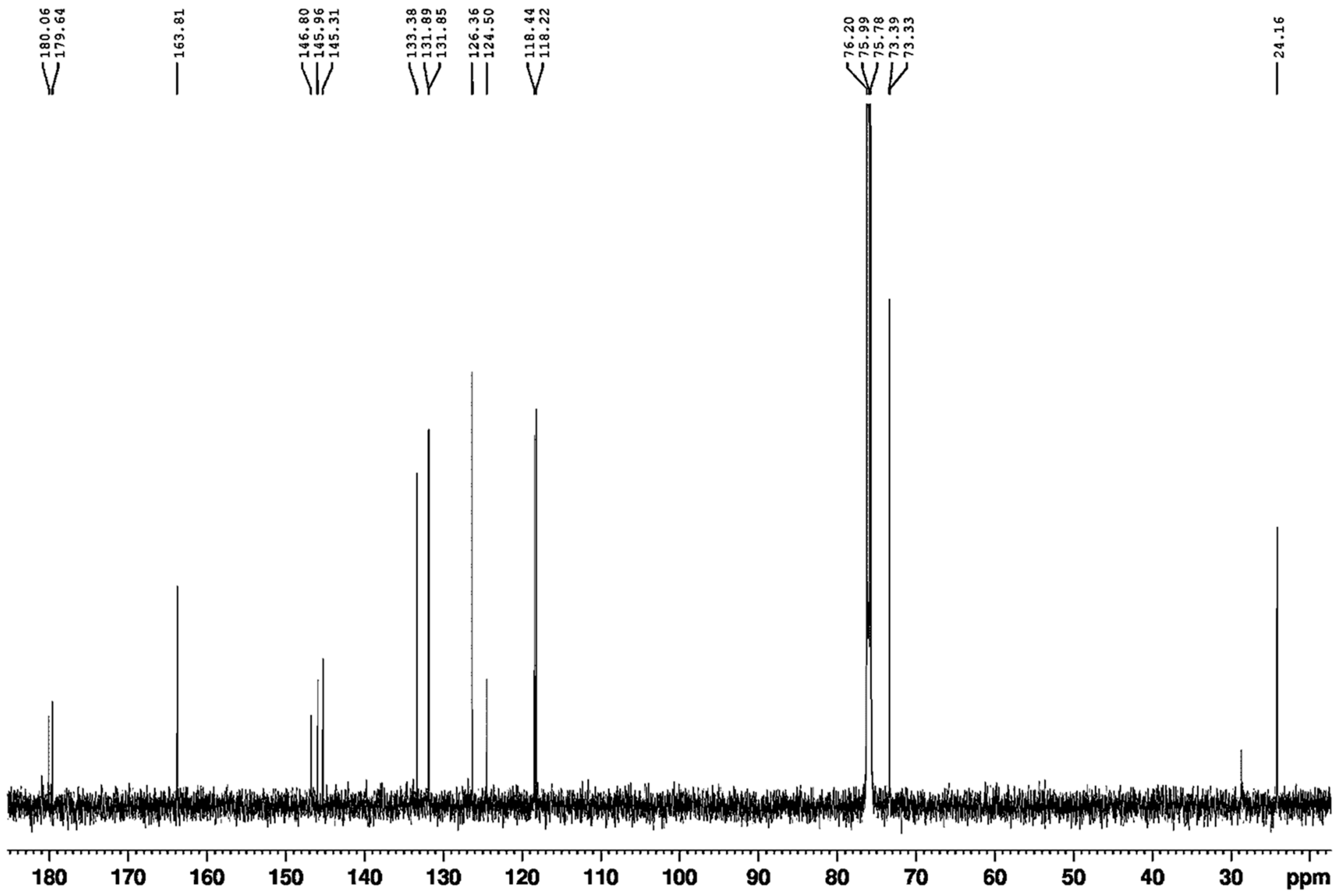

Figure S9 (b): ^{13}C-NMR spectrum of 2-methyl-6,7-di(2-propenoxy)-5,8-quinolinedione (**20**).

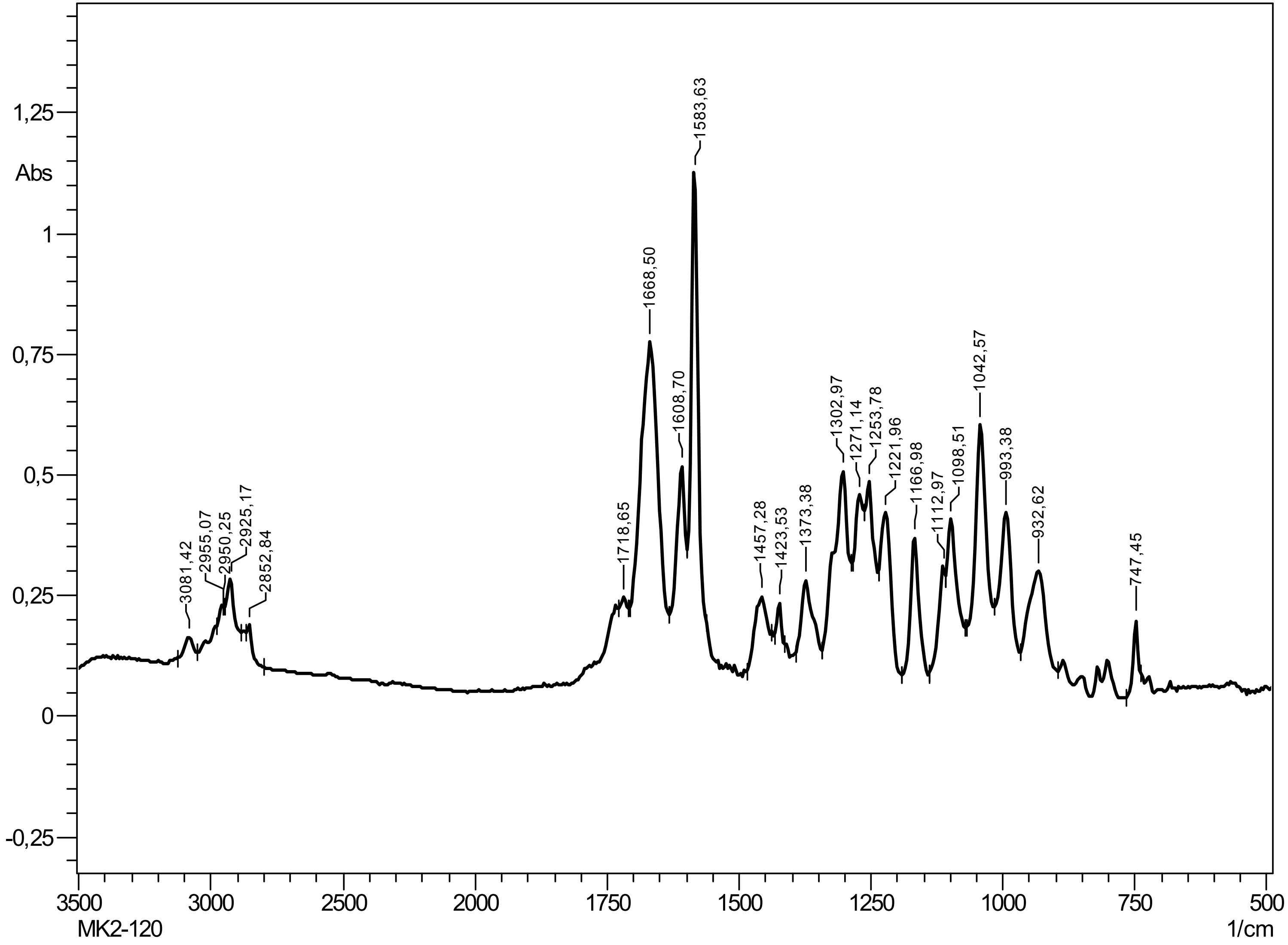

Figure S9 (c): IR spectrum of 2-methyl-6,7-di(2-propenoxy)-5,8-quinolinedione (**20**).

Compound Spectrum List Report

Analysis Info

Analysis Name	D:\Data\mk20.d	Acquisition Date	2/17/2017 11:49:27 AM
Method	low_mass.m	Operator	KM
Sample Name	TM Low concentration	Instrument	impact II 1825265.10082
Comment			

Acquisition Parameter

Source Type	ESI	Ion Polarity	Positive	Set Nebulizer	0.3 Bar
Focus	Active	Set Capillary	4000 V	Set Dry Heater	240 °C
Scan Begin	100 m/z	Set End Plate Offset	-500 V	Set Dry Gas	4.0 l/min
Scan End	1000 m/z	Set Charging Voltage	2000 V	Set Divert Valve	Source
		Set Corona	0 nA	Set APCI Heater	0 °C

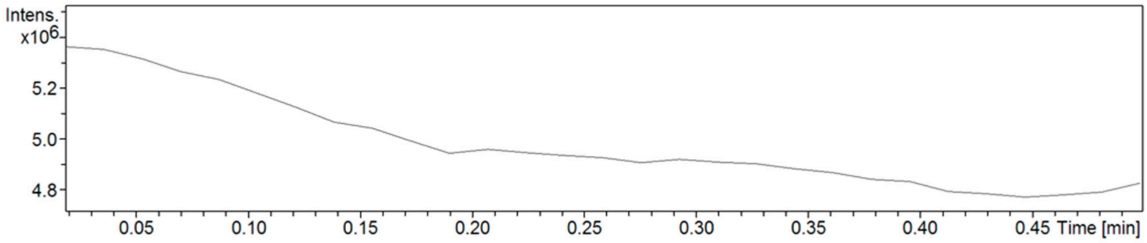

+MS, 0.0-0.5min #1-29

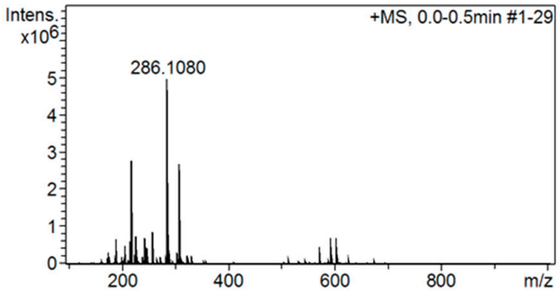

#	m/z	Res.	S/N	I	I %	FWHM
1	218.0814	33570	24482.1	2784530	55.8	0.0065
2	286.1080	38718	35629.9	4988728	100.0	0.0074

Figure S9 (d): HR-MS spectrum of 2-methyl-6,7-di(2-propenoxy)-5,8-quinolinedione (**20**).

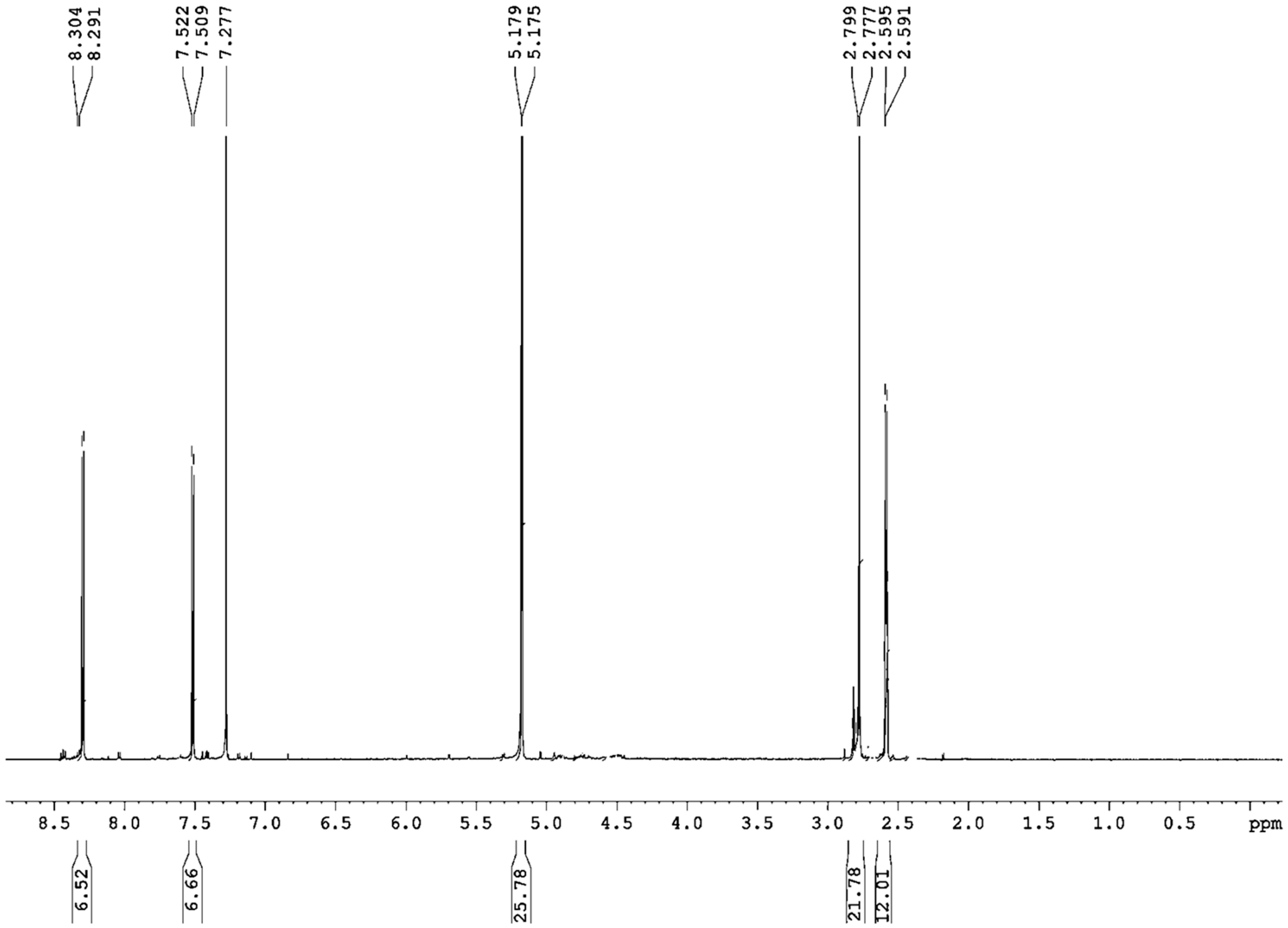

Figure S10 (a): ^{1}H-NMR spectrum of 2-methyl-6,7-di(2-propynoxy)-5,8-qunolinedione (**21**).

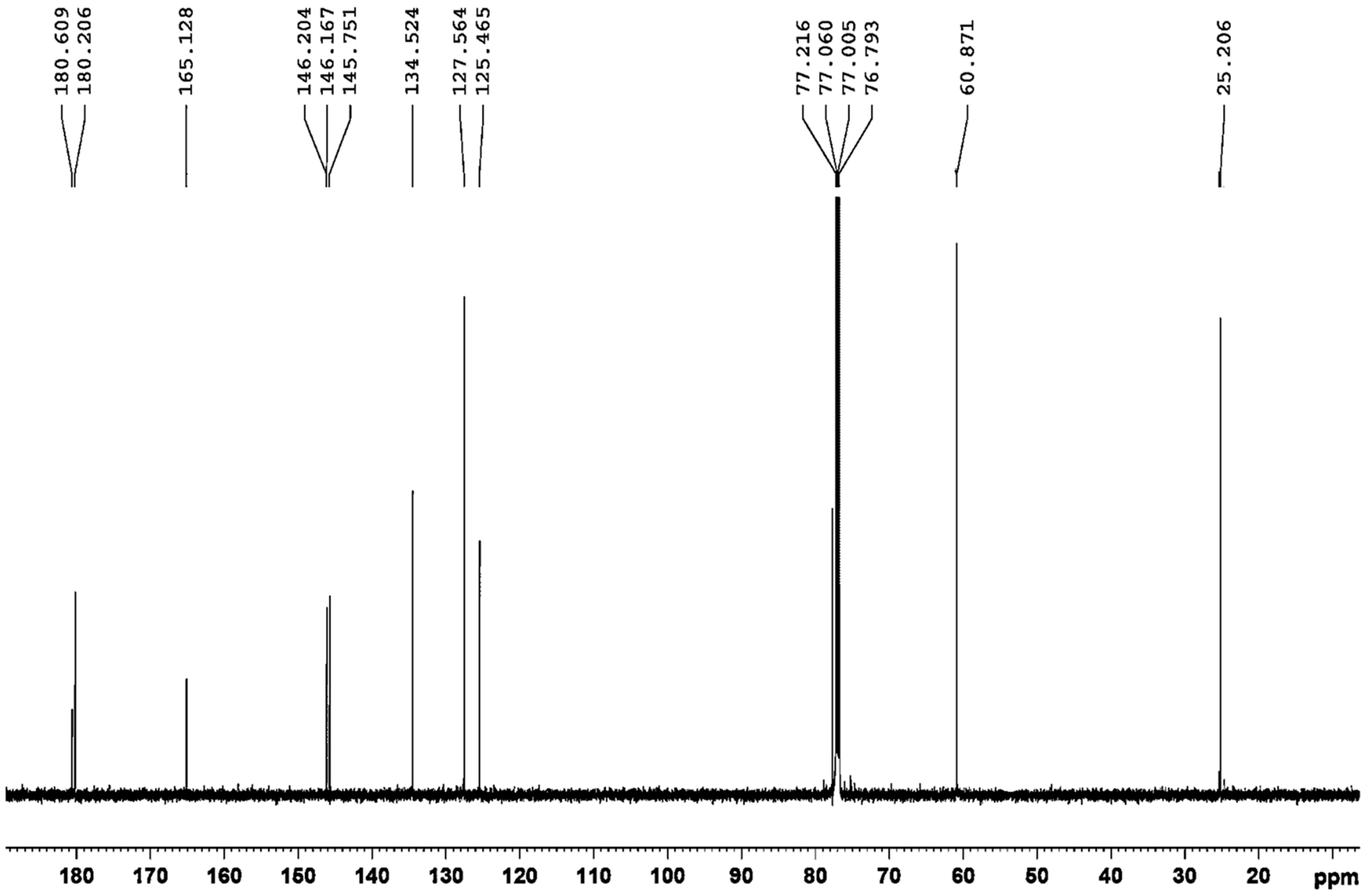

Figure S10 (b): ^{13}C-NMR spectrum of 2-methyl-6,7-di(2-propynoxy)-5,8-qunolinedione (**21**).

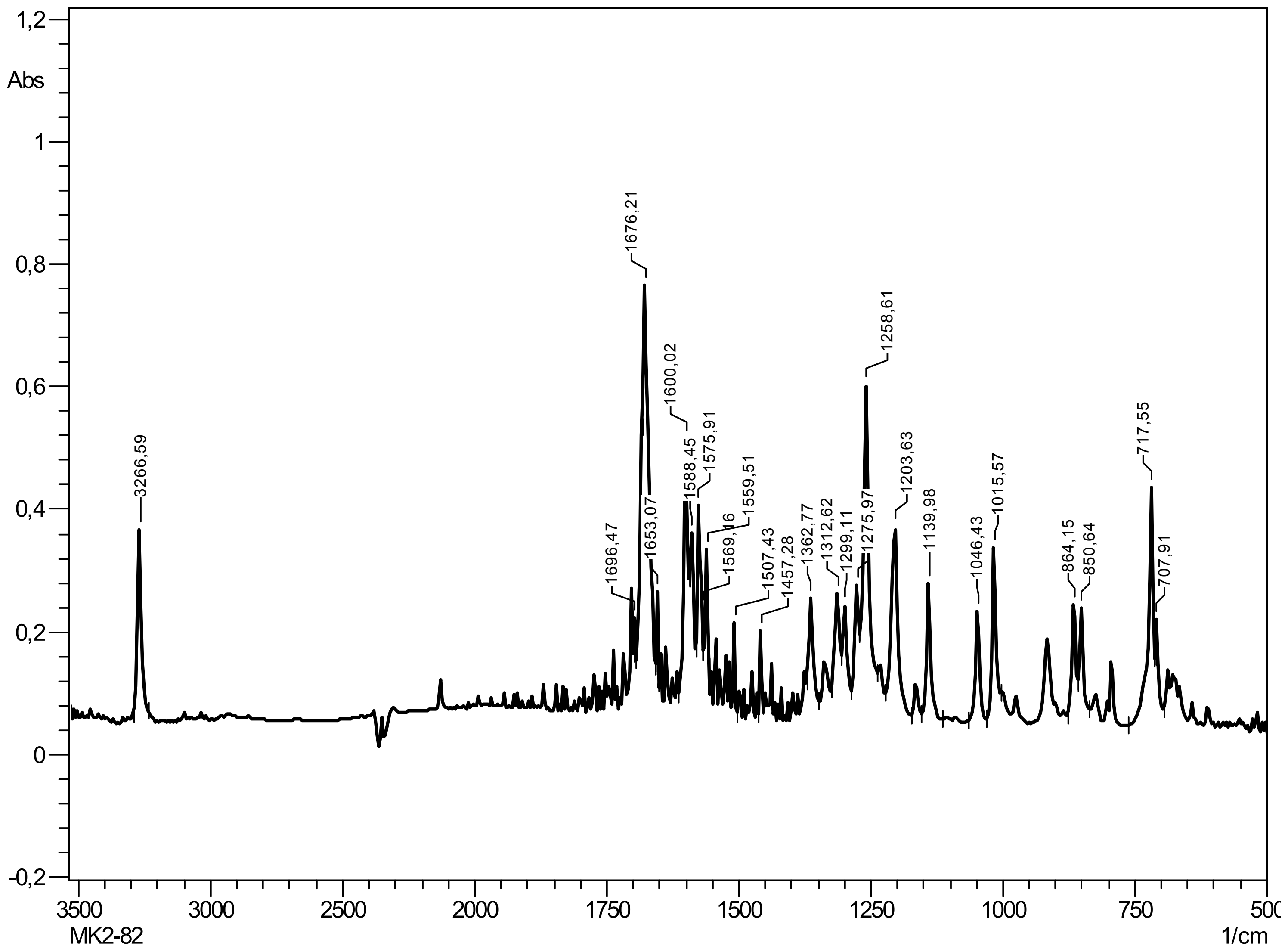

Figure S10 (c): IR spectrum of 2-methyl-6,7-di(2-propynoxy)-5,8-qunolinedione (**21**).

Compound Spectrum List Report

Analysis Info

Analysis Name	D:\Data\mk21.d	Acquisition Date	2/17/2017 11:51:42 AM
Method	low_mass.m	Operator	KM
Sample Name	TM Low concentration	Instrument	impact II 1825265.10082
Comment			

Acquisition Parameter

Source Type	ESI	Ion Polarity	Positive	Set Nebulizer	0.3 Bar
Focus	Active	Set Capillary	4000 V	Set Dry Heater	240 °C
Scan Begin	100 m/z	Set End Plate Offset	-500 V	Set Dry Gas	4.0 l/min
Scan End	1000 m/z	Set Charging Voltage	2000 V	Set Divert Valve	Source
		Set Corona	0 nA	Set APCI Heater	0 °C

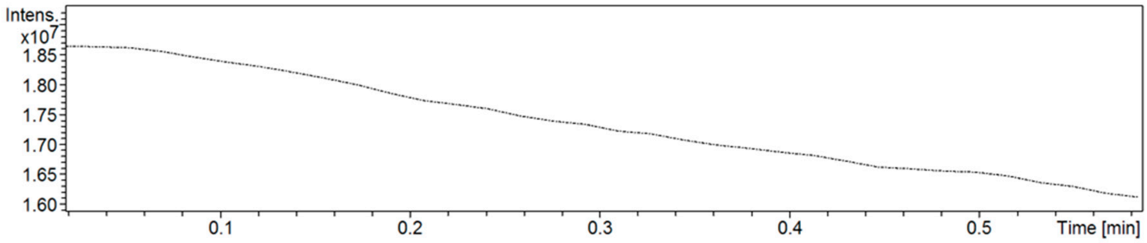

+MS, 0.0-0.6min #1-34

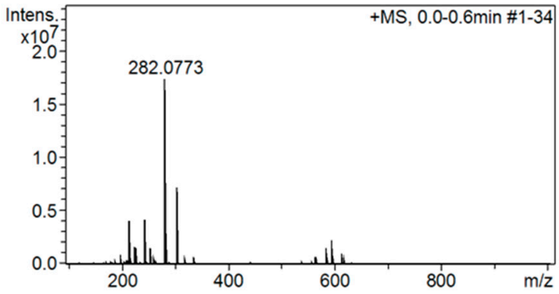

#	m/z	Res.	S/N	I	I %	FWHM
1	215.0583	34252	28665.4	4067549	23.4	0.0063
2	243.0535	36356	24559.0	4189061	24.1	0.0067
3	282.0773	36894	81856.9	17400966	100.0	0.0076

Figure S10 (d): HR-MS spectrum of 2-methyl-6,7-di(2-propynoxy)-5,8-qunolinedione (**21**).

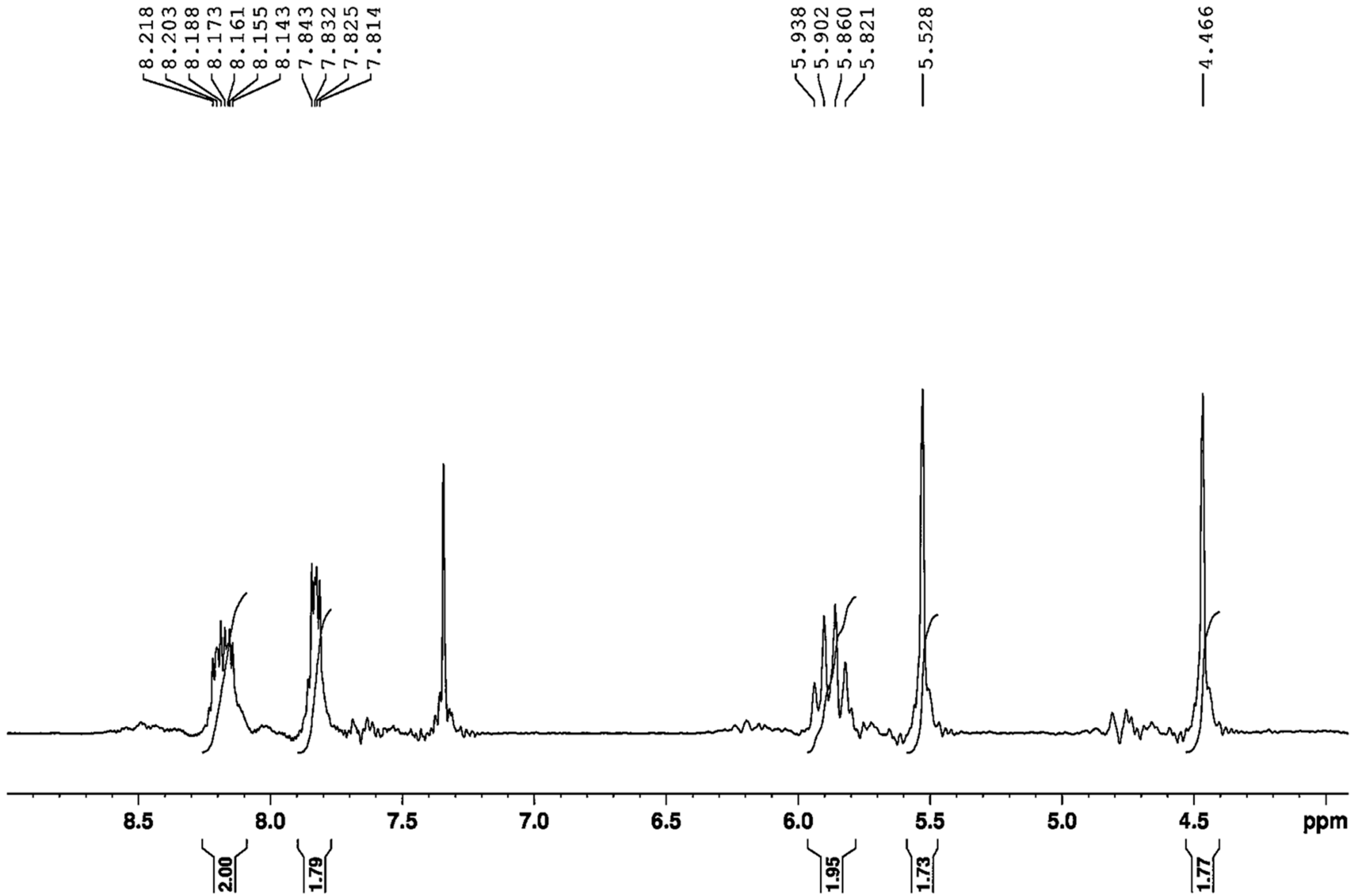

Figure S11 (a): ^{13}C-NMR spectrum of 2-chloro-3-(8-hydroxy-4-octen-2,6-diynyloxy)-1,4-naphtoquinon (**23**).

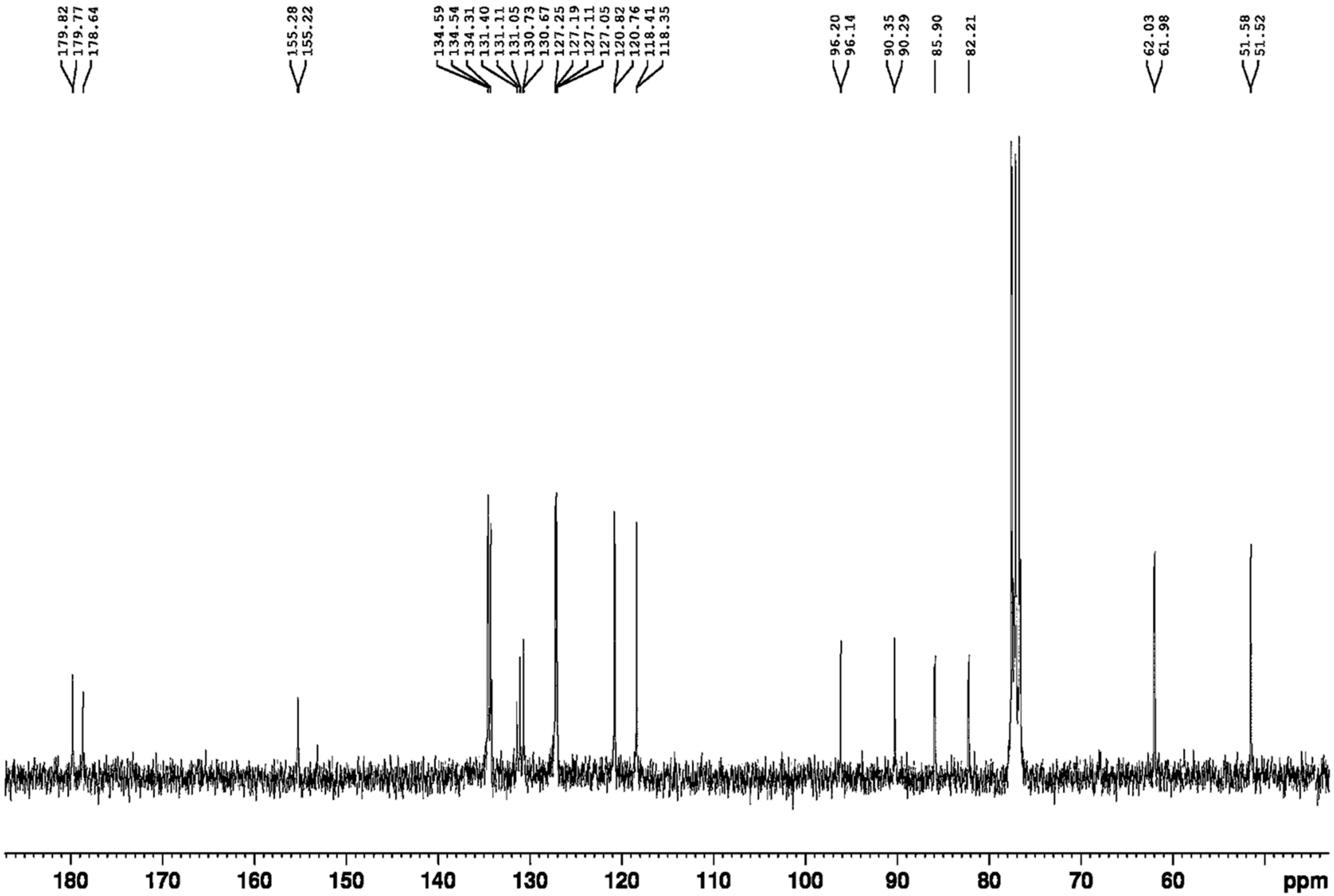

Figure S11 (b): ^{13}C-NMR spectrum of 2-chloro-3-(8-hydroxy-4-octen-2,6-diynyloxy)-1,4-naphtoquinon (**23**).

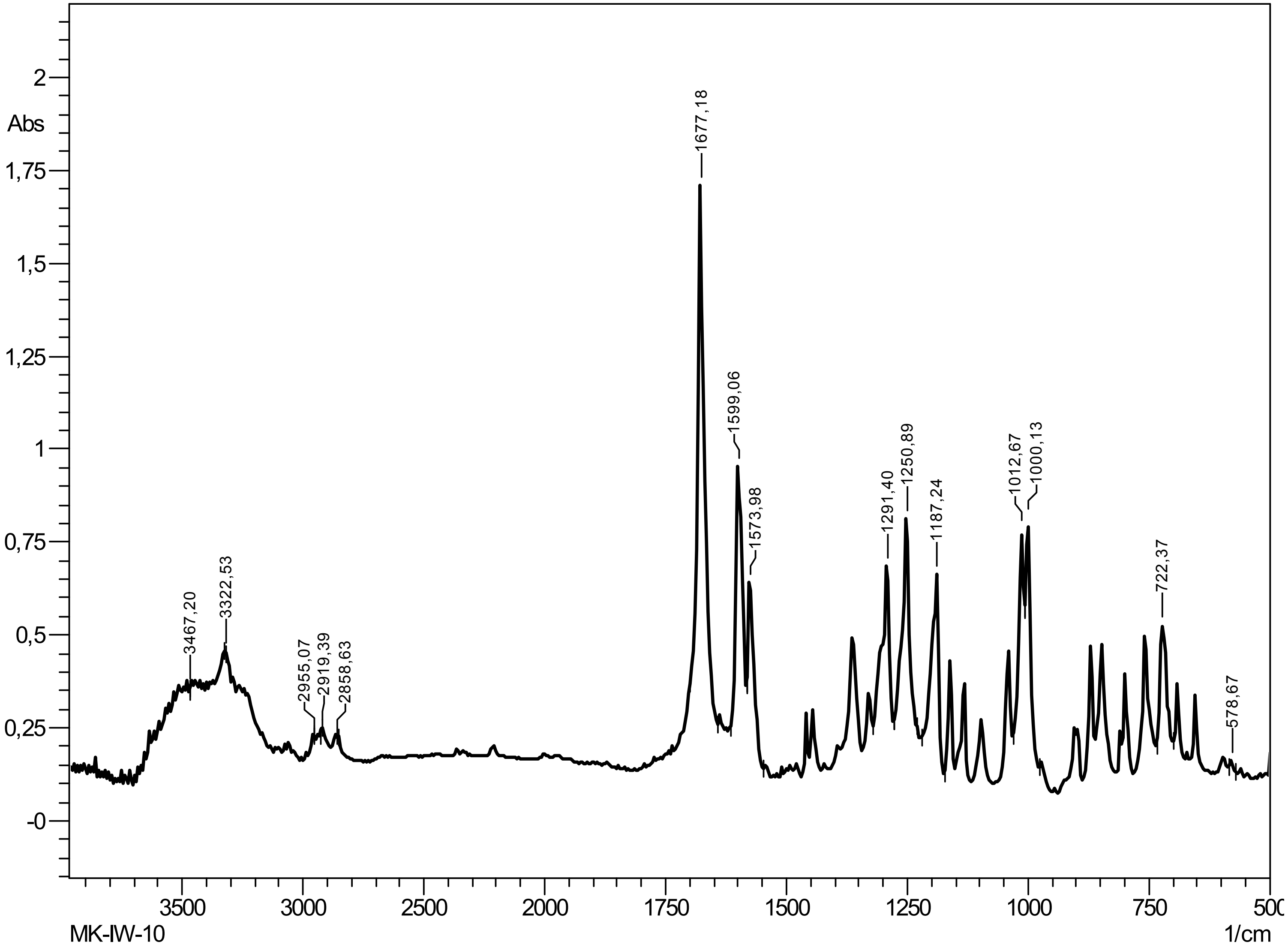

Figure S11 (c): IR spectrum of 2-chloro-3-(8-hydroxy-4-octen-2,6-diynyloxy)-1,4-naphtoquinon (**23**).

Compound Spectrum List Report

Analysis Info

Analysis Name	D:\Data\mk23.d	Acquisition Date	2/17/2017 11:54:33 AM
Method	low_mass.m	Operator	KM
Sample Name	TM Low concentration	Instrument	impact II 1825265.10082
Comment			

Acquisition Parameter

Source Type	ESI	Ion Polarity	Positive	Set Nebulizer	0.3 Bar
Focus	Active	Set Capillary	4000 V	Set Dry Heater	240 °C
Scan Begin	100 m/z	Set End Plate Offset	-500 V	Set Dry Gas	4.0 l/min
Scan End	1000 m/z	Set Charging Voltage	2000 V	Set Divert Valve	Source
		Set Corona	0 nA	Set APCI Heater	0 °C

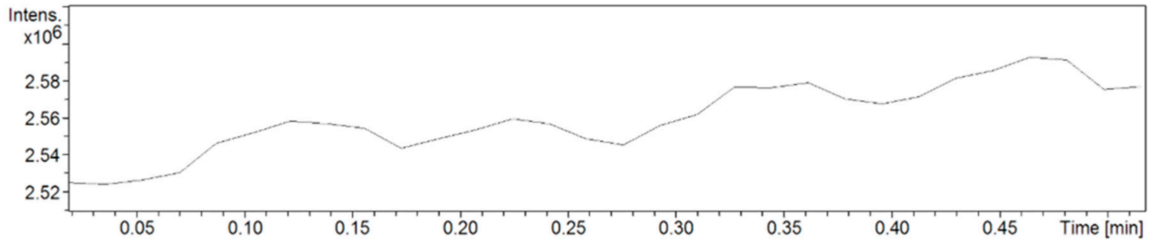

+MS, 0.0-0.5min #1-30

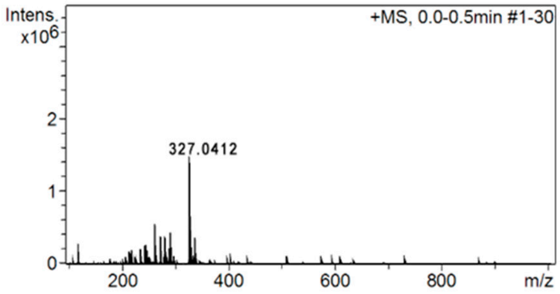

#	m/z	Res.	S/N	I	I %	FWHM
1	327.0412	36833	9911.9	1462938	100.0	0.0084

Figure S11 (d): HR-MS spectrum of 2-chloro-3-(8-hydroxy-4-octen-2,6-diynyloxy)-1,4-naphtoquinon (**23**).

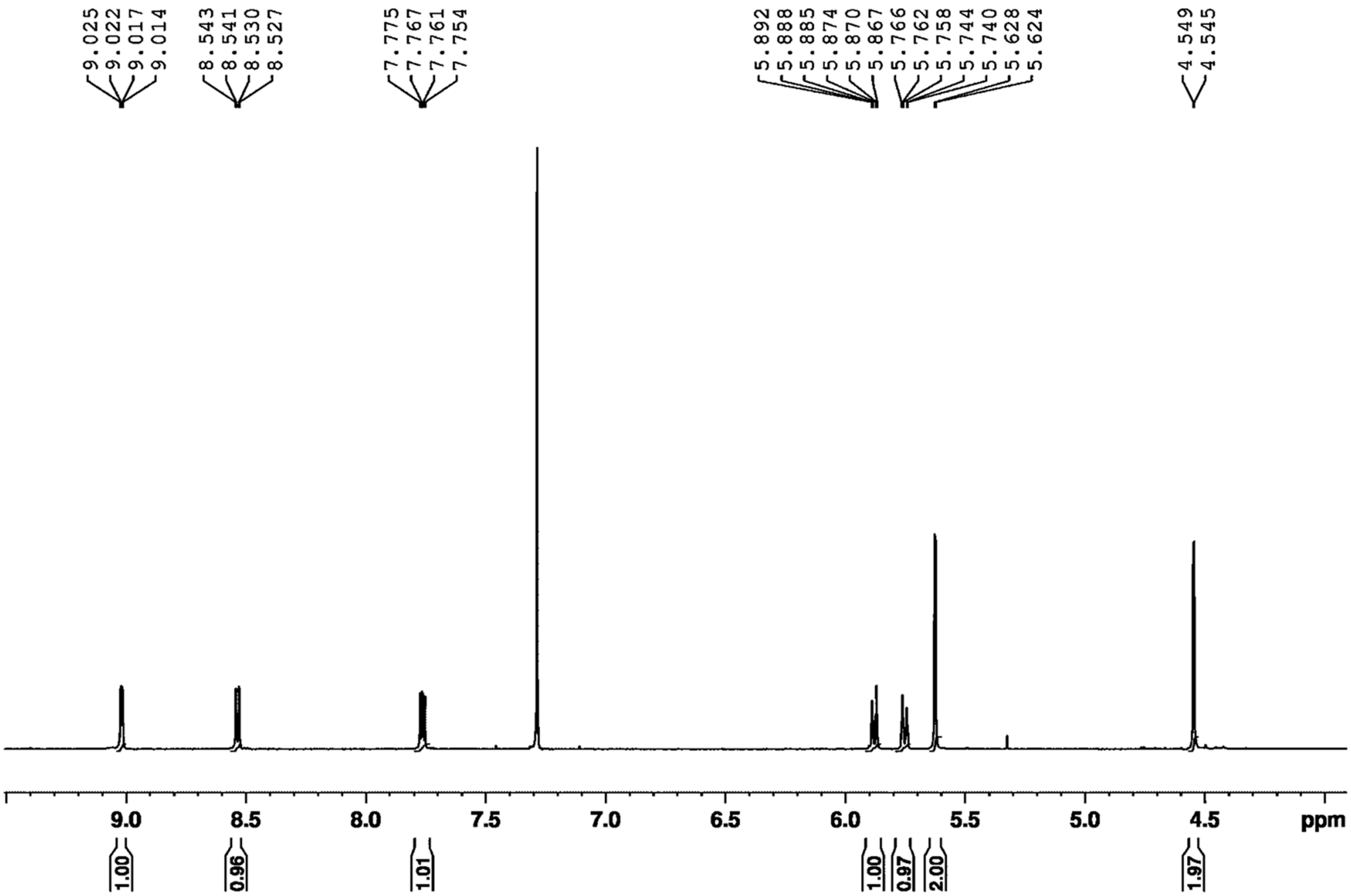

Figure S12 (a): ^{1}H-NMR spectrum of 6-chloro-7-(8-hydroxy-4-octen-2,6-diynyloxy)-5,8-quinolinedione (**24**).

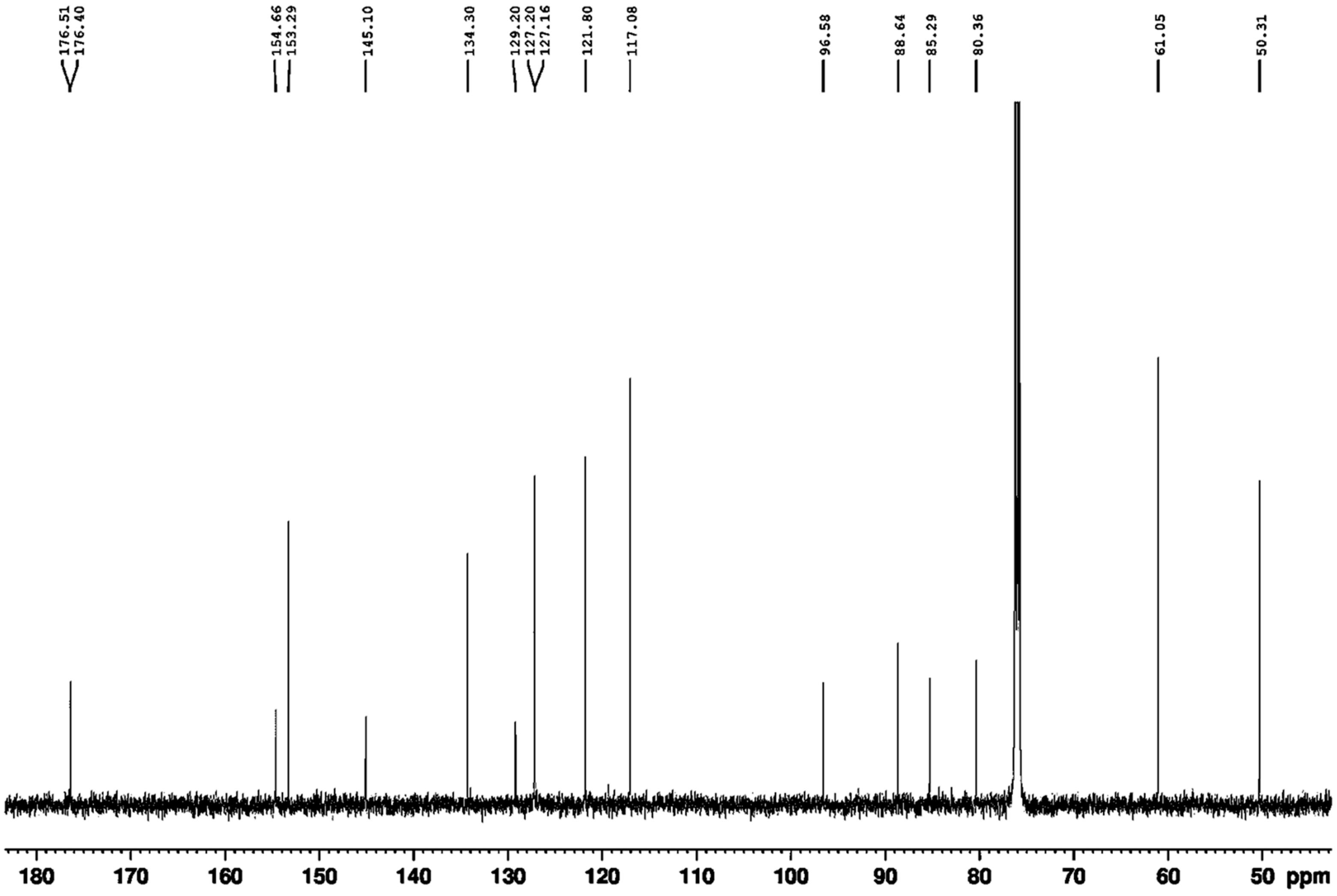

Figure S12 (b): ^{1}H-NMR spectrum of 6-chloro-7-(8-hydroxy-4-octen-2,6-diynyloxy)-5,8-quinolinedione (**24**).

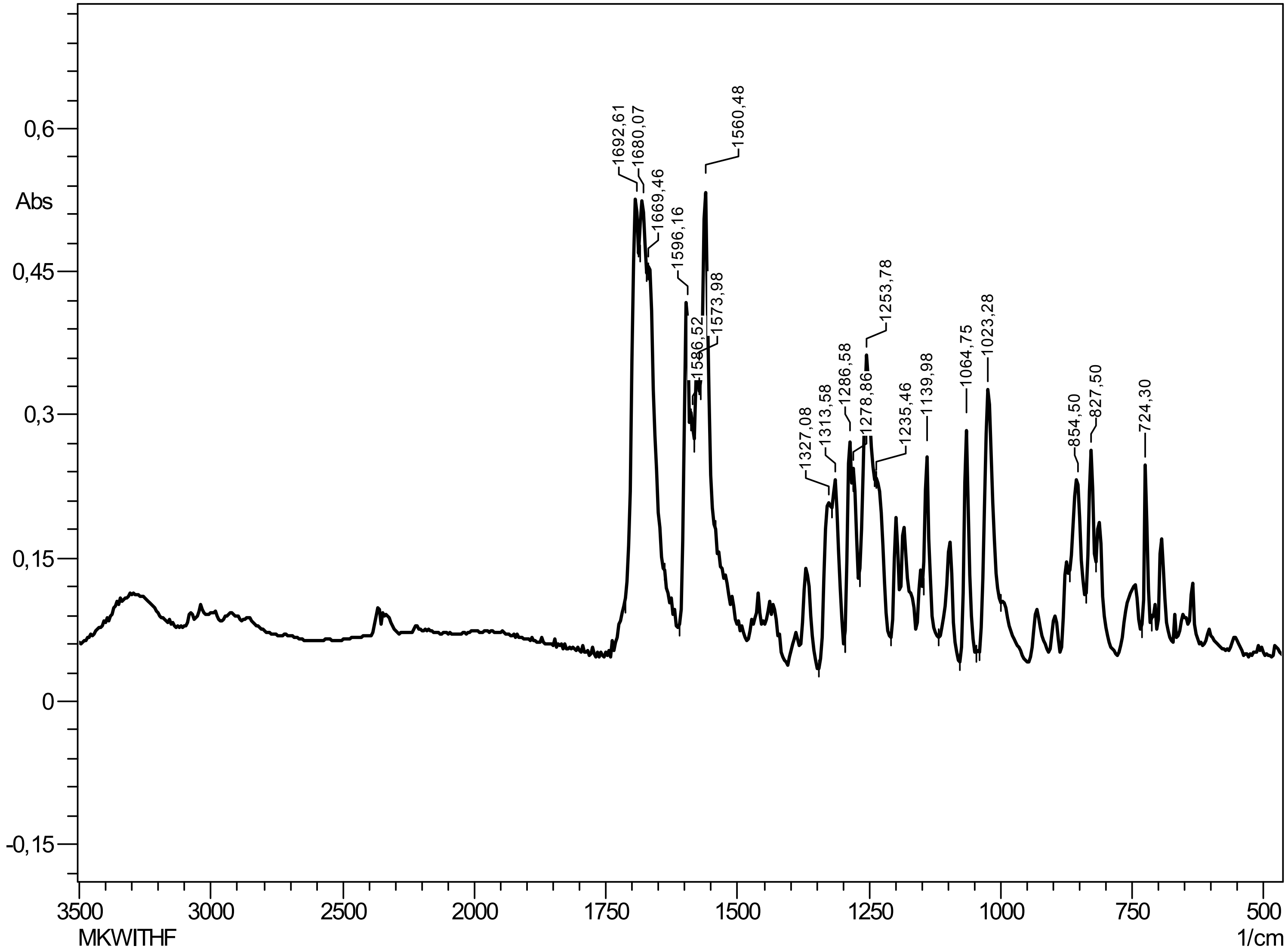

Figure S12 (c): IR spectrum of 6-chloro-7-(8-hydroxy-4-octen-2,6-diynyloxy)-5,8-quinolinedione (**24**).

Compound Spectrum List Report

Analysis Info

Analysis Name	D:\Data\mk24.d	Acquisition Date	2/17/2017 11:56:47 AM	
Method	low_mass.m	Operator	KM	
Sample Name	TM Low concentration	Instrument	impact II	1825265.10082
Comment				

Acquisition Parameter

Source Type	ESI	Ion Polarity	Positive	Set Nebulizer	0.3 Bar
Focus	Active	Set Capillary	4000 V	Set Dry Heater	240 °C
Scan Begin	100 m/z	Set End Plate Offset	-500 V	Set Dry Gas	4.0 l/min
Scan End	1000 m/z	Set Charging Voltage	2000 V	Set Divert Valve	Source
		Set Corona	0 nA	Set APCI Heater	0 °C

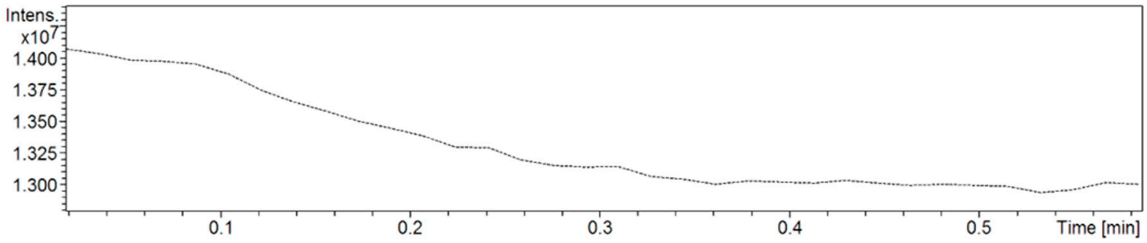

+MS, 0.0-0.6min #1-34

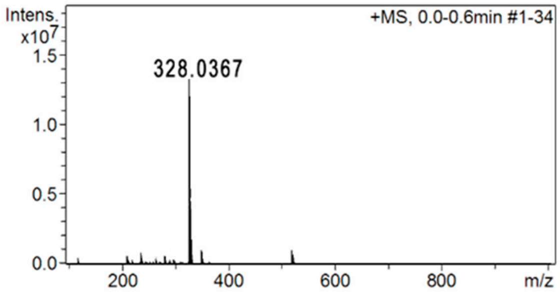

#	m/z	Res.	S/N	I	I %	FWHM
1	328.0367	39636	18184.0	3513484	100.0	0.0078

Figure S12 (d): HR-MS spectrum of 6-chloro-7-(8-hydroxy-4-octen-2,6-diynyloxy)-5,8-quinolinedione (**24**).

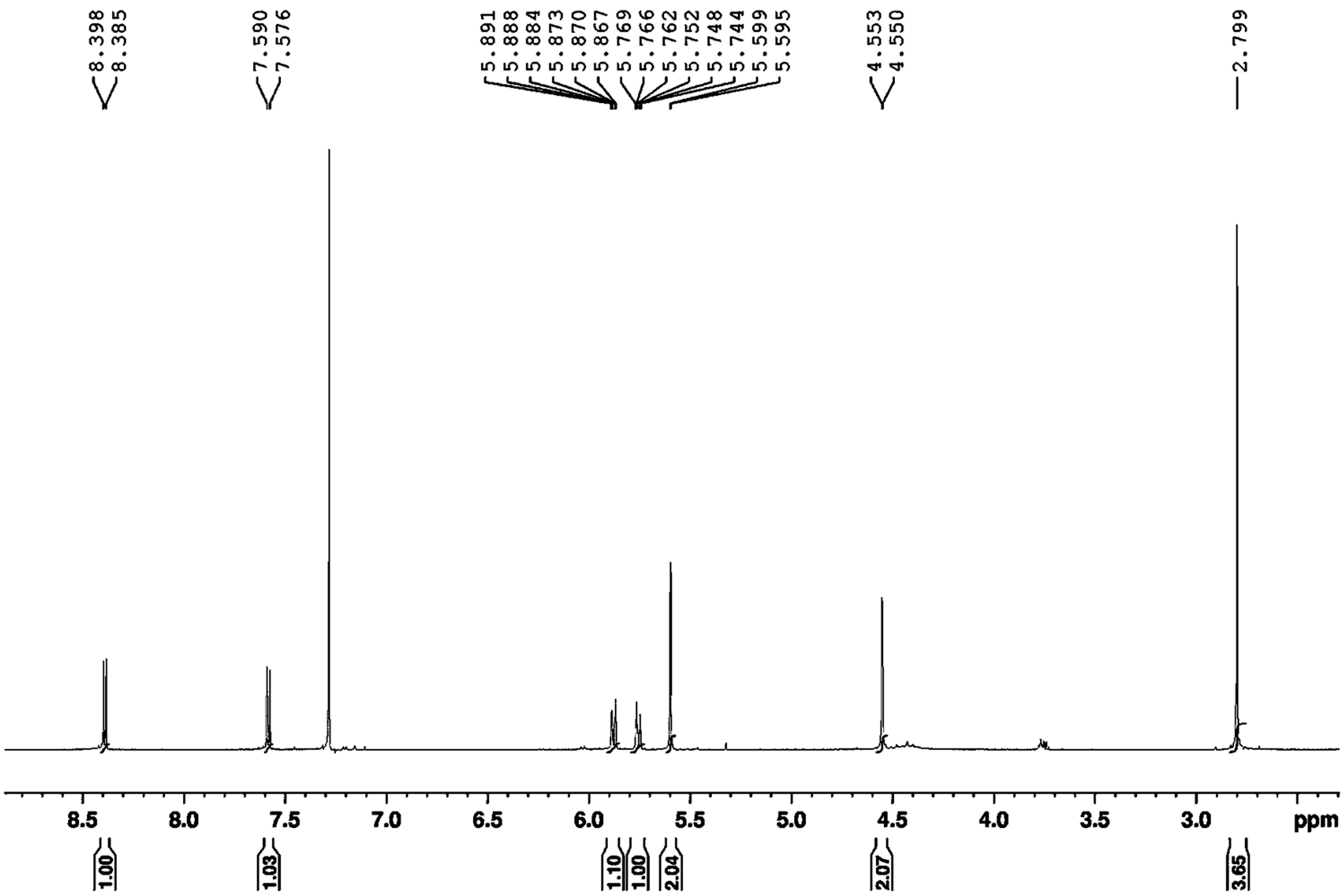

Figure S13 (a): ^{1}H-NMR spectrum of 6-chloro-7-(8-hydroxy-4-octen-2,6-diynyloxy)-2-methyl-5,8-quinolinedione (**25**).

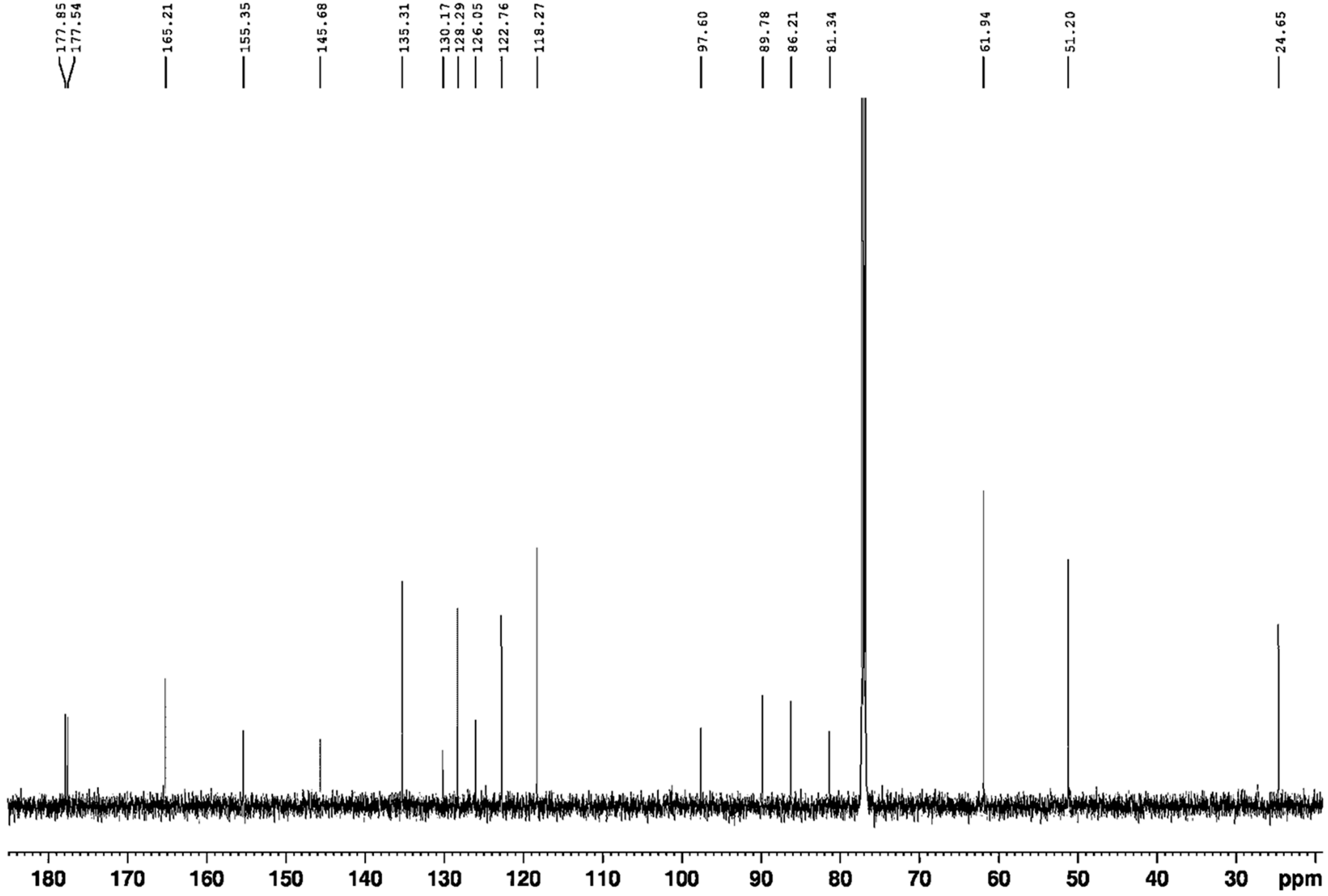

Figure S13 (b): ^{13}C-NMR spectrum of 6-chloro-7-(8-hydroxy-4-octen-2,6-diynyloxy)-2-methyl-5,8-quinolinedione (**25**).

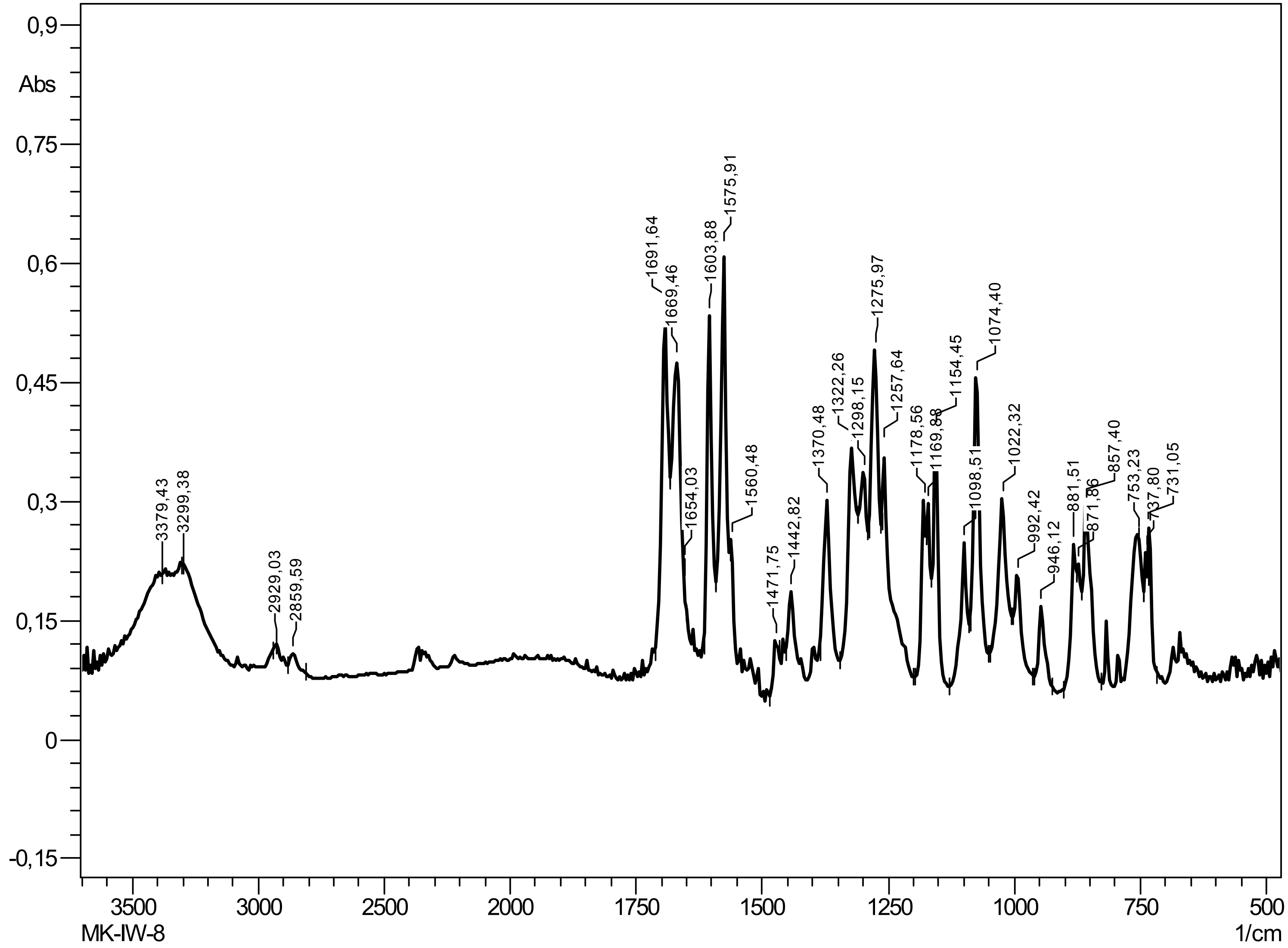

Figure S13 (c): IR spectrum of 6-chloro-7-(8-hydroxy-4-octen-2,6-diynyloxy)-2-methyl-5,8-quinolinedione (**25**).

Compound Spectrum List Report

Analysis Info

Analysis Name	D:\Data\mk25.d	Acquisition Date	2/17/2017 12:00:40 PM	
Method	low_mass.m	Operator	KM	
Sample Name	TM Low concentration	Instrument	impact II	1825265.10082
Comment				

Acquisition Parameter

Source Type	ESI	Ion Polarity	Positive	Set Nebulizer	0.3 Bar
Focus	Active	Set Capillary	4000 V	Set Dry Heater	240 °C
Scan Begin	100 m/z	Set End Plate Offset	-500 V	Set Dry Gas	4.0 l/min
Scan End	1000 m/z	Set Charging Voltage	2000 V	Set Divert Valve	Source
		Set Corona	0 nA	Set APCI Heater	0 °C

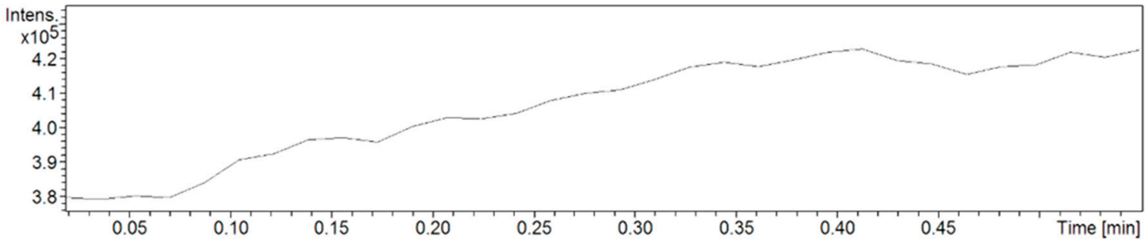

+MS, 0.0-0.5min #1-32

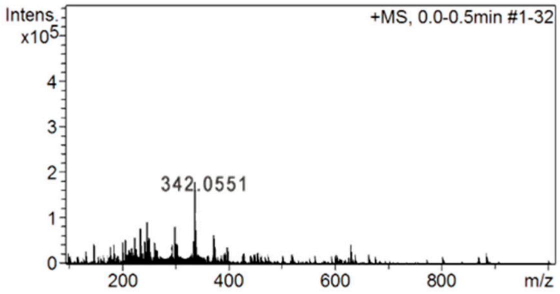

#	m/z	Res.	S/N	I	I %	FWHM
1	342.0551	32280	3301.4	406672	100.0	0.0097

Figure S13 (d): HR-MS spectrum of 6-chloro-7-(8-hydroxy-4-octen-2,6-diynyloxy)-2-methyl-5,8-quinolinedione (**25**).

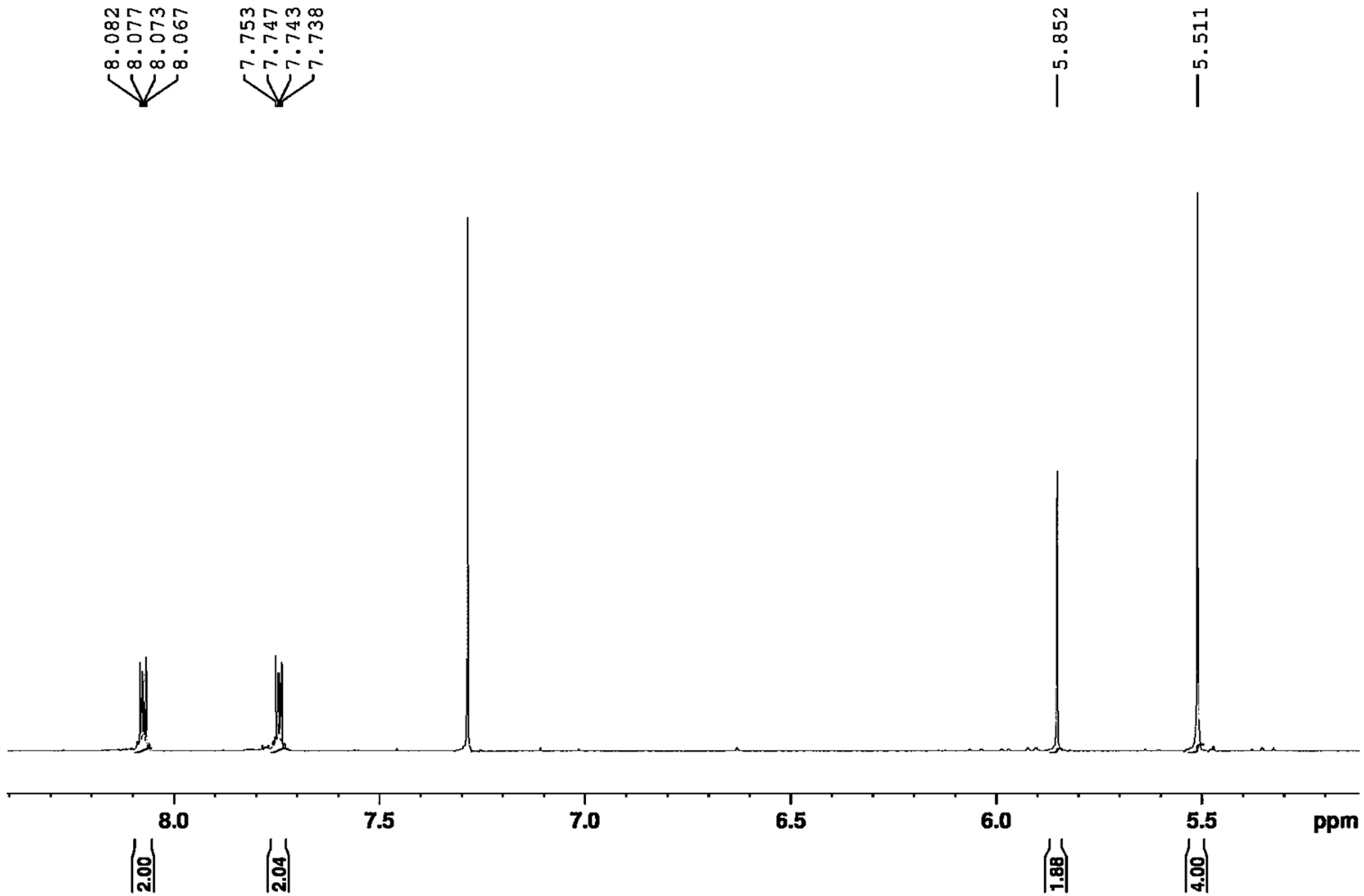

Figure S14 (a): ^{1}H-NMR spectrum of 8-octen-6,10-diynyl-1,4-dioxocyclododeca[2,3-g]-1,4-naphthoquinon (**26**).

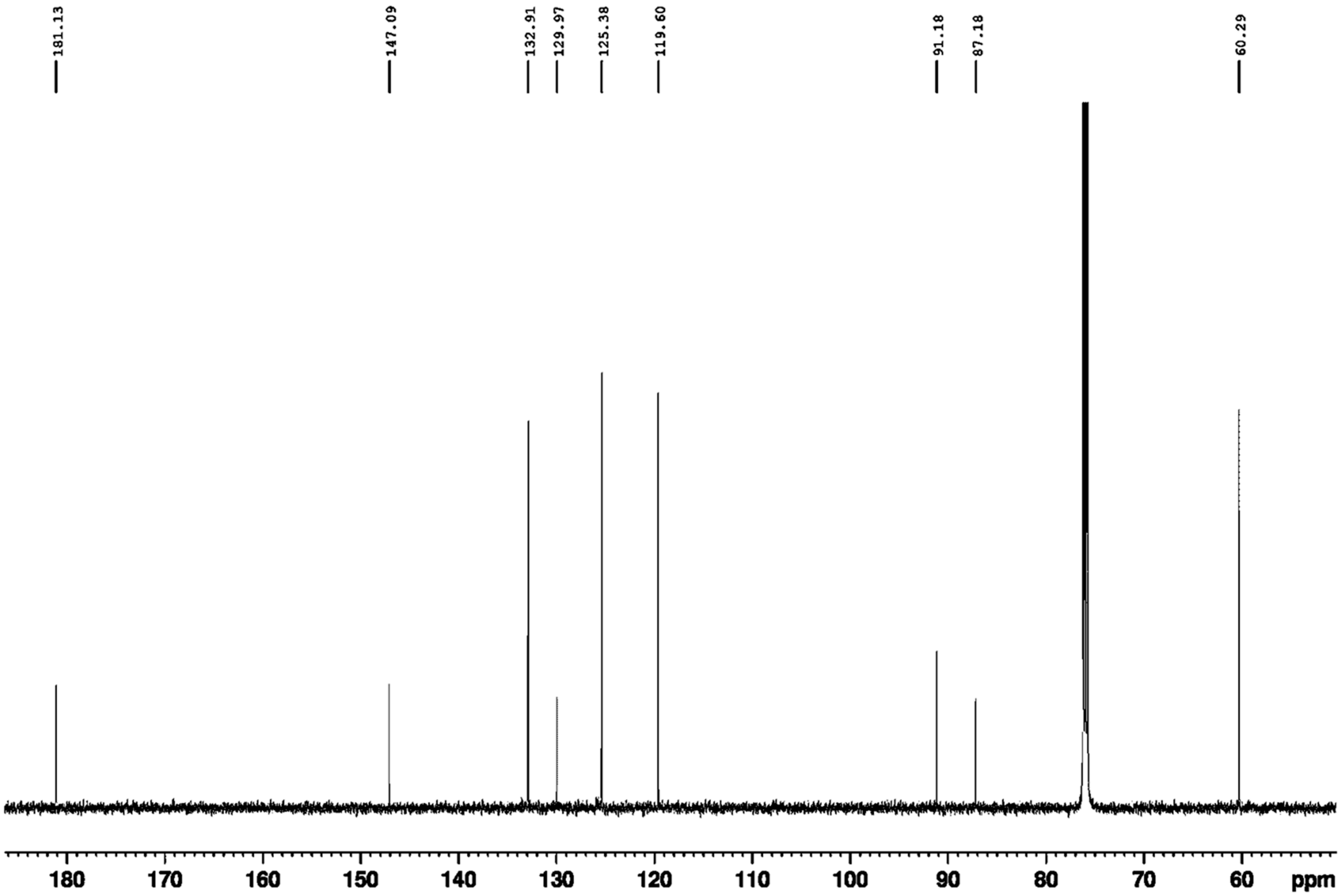

Figure S14 (b): ^{13}C-NMR spectrum of 8-octen-6,10-diynyl-1,4-dioxocyclododeca[2,3-g]-1,4-naphthoquinon (**26**).

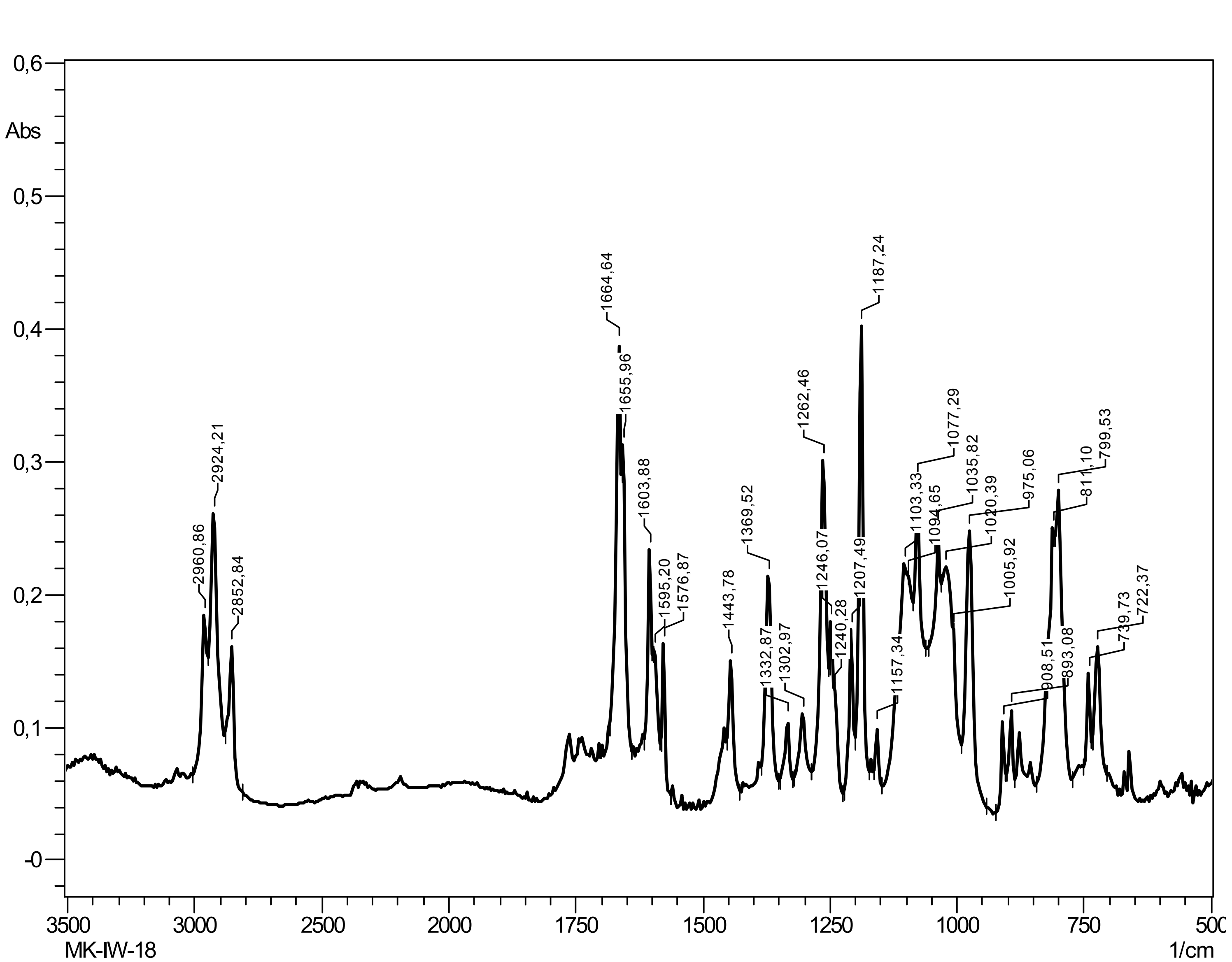

Figure S14 (c): IR spectrum of 8-octen-6,10-diynyl-1,4-dioxocyclododeca[2,3-g]-1,4-naphthoquinon (**26**).

Compound Spectrum List Report

Analysis Info

Analysis Name	D:\Data\mk26.d	Acquisition Date	2/17/2017 12:03:36 PM
Method	low_mass.m	Operator	KM
Sample Name	TM Low concentration	Instrument	impact II 1825265.10082
Comment			

Acquisition Parameter

Source Type	ESI	Ion Polarity	Positive	Set Nebulizer	0.3 Bar
Focus	Active	Set Capillary	4000 V	Set Dry Heater	240 °C
Scan Begin	100 m/z	Set End Plate Offset	-500 V	Set Dry Gas	4.0 l/min
Scan End	1000 m/z	Set Charging Voltage	2000 V	Set Divert Valve	Source
		Set Corona	0 nA	Set APCI Heater	0 °C

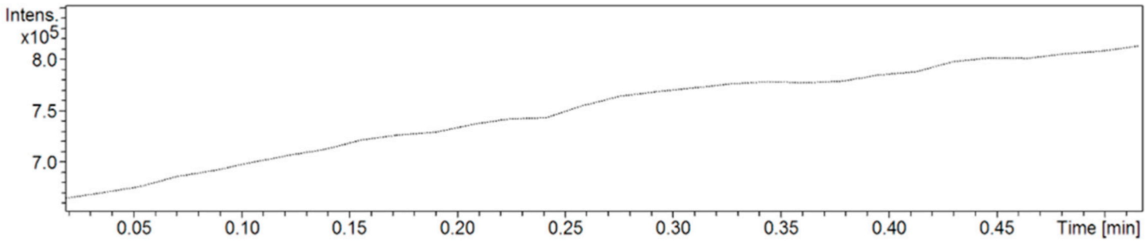

+MS, 0.0-0.5min #1-30

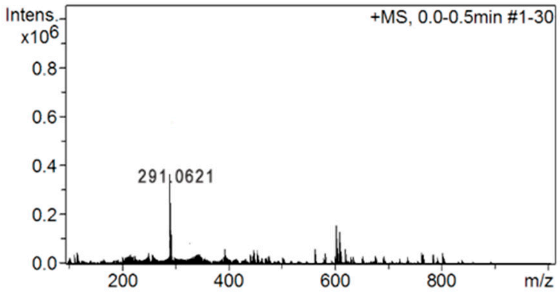

#	m/z	Res.	S/N	I	I %	FWHM
1	291.0621	32157	4727.8	554451	100.0	0.0085

Figure S14 (d): HR-MS spectrum of 8-octen-6,10-diynyl-1,4-dioxocyclododeca[2,3-g]-1,4-naphthoquinon (**26**).

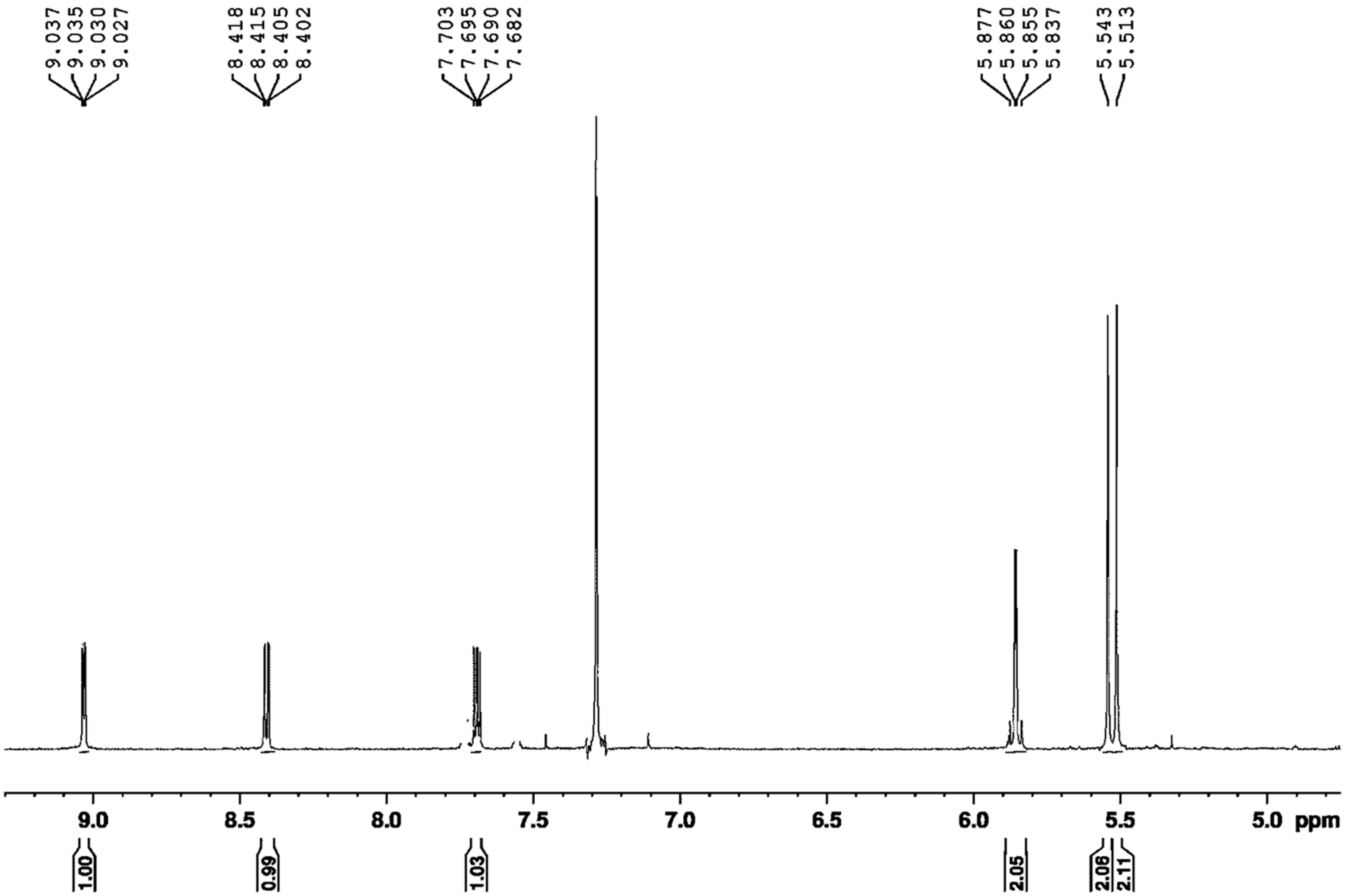

Figure S15 (a): ^{13}C-NMR spectrum of 8-octen-6,10-diynyl-1,4-dioxocyclododeca[2,3-g]-5,8-quinolinedione (**27**).

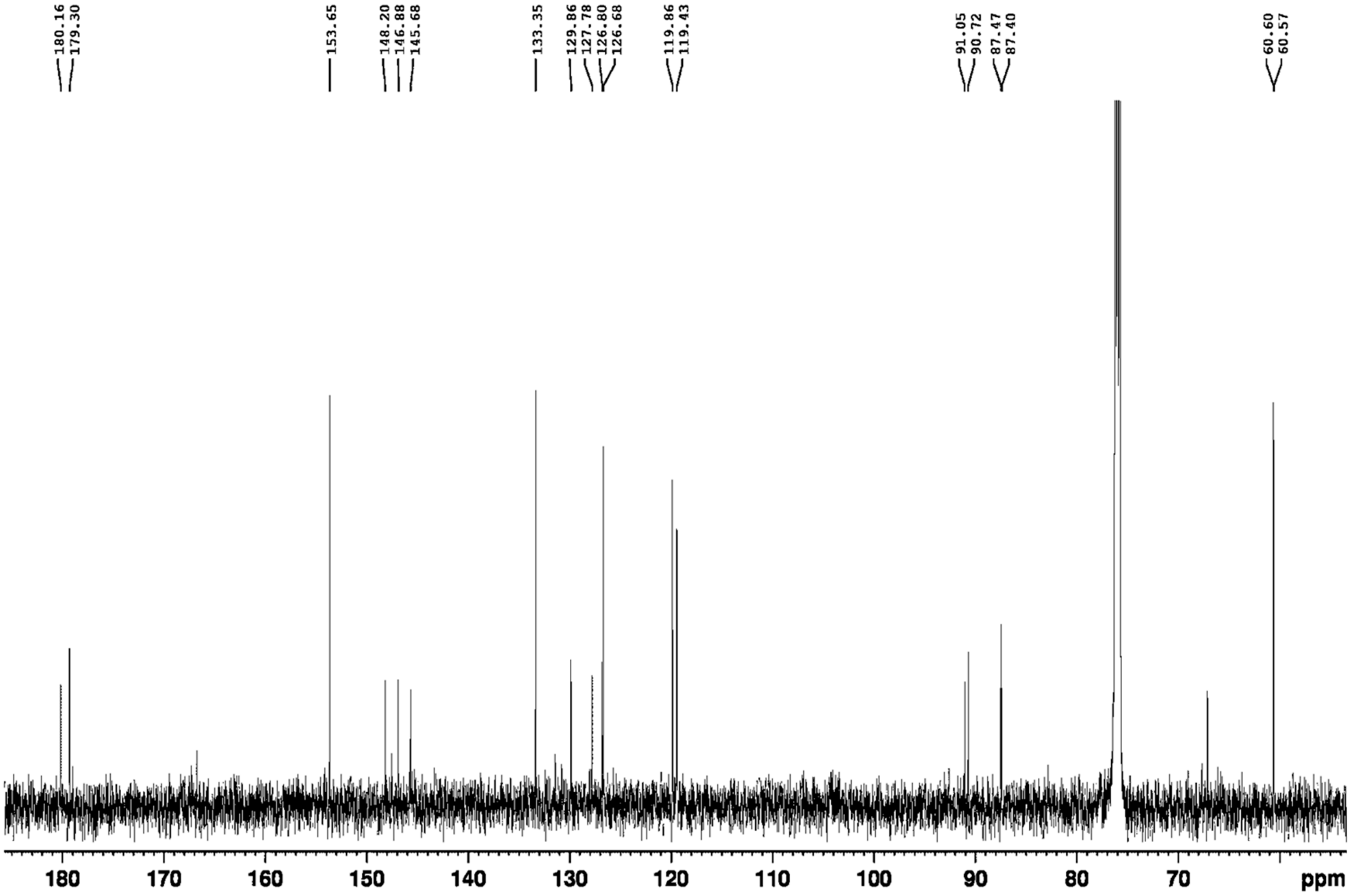

Figure S15 (b): ^{1}H-NMR spectrum of 8-octen-6,10-diynyl-1,4-dioxocyclododeca[2,3-g]-5,8-quinolinedione (**27**).

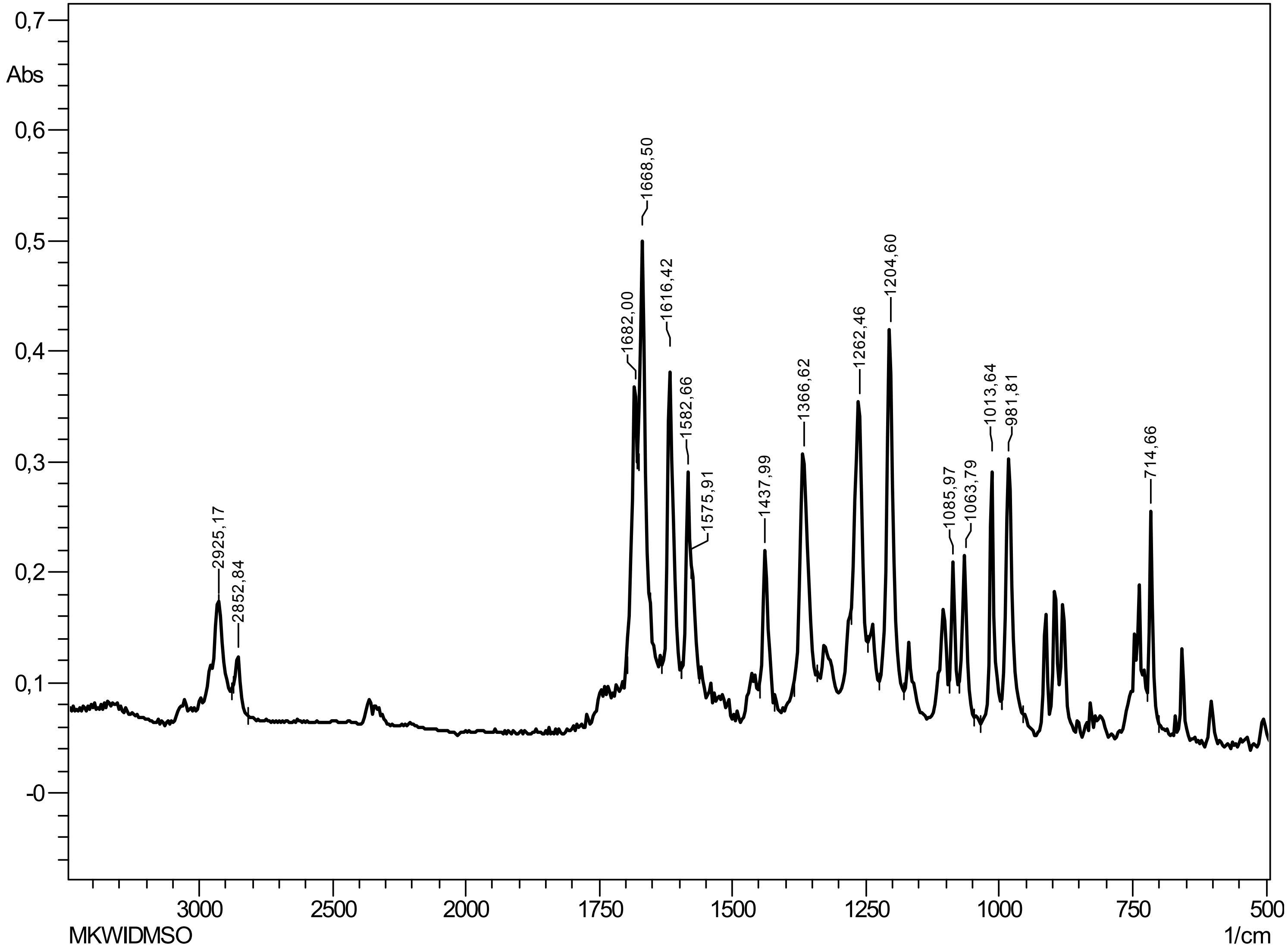

Figure S15 (c): IR spectrum of 8-octen-6,10-diynyl-1,4-dioxocyclododeca[2,3-g]-5,8-quinolinedione (**27**).

Compound Spectrum List Report

Analysis Info

Analysis Name	D:\Data\mk27.d	Acquisition Date	2/17/2017 12:06:09 PM	
Method	low_mass.m	Operator	KM	
Sample Name	TM Low concentration	Instrument	impact II	1825265.10082
Comment				

Acquisition Parameter

Source Type	ESI	Ion Polarity	Positive	Set Nebulizer	0.3 Bar
Focus	Active	Set Capillary	4000 V	Set Dry Heater	240 °C
Scan Begin	100 m/z	Set End Plate Offset	-500 V	Set Dry Gas	4.0 l/min
Scan End	1000 m/z	Set Charging Voltage	2000 V	Set Divert Valve	Source
		Set Corona	0 nA	Set APCI Heater	0 °C

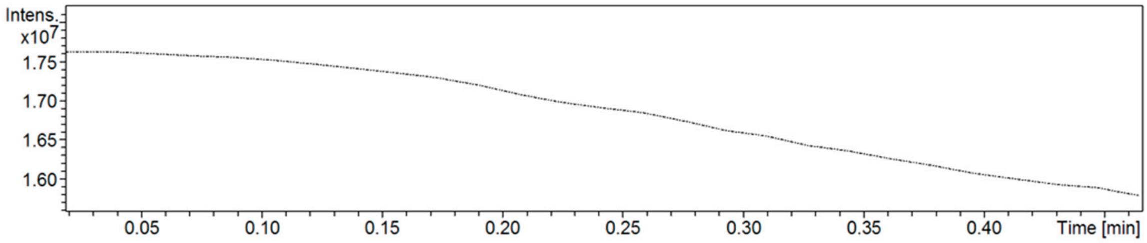

+MS, 0.0-0.5min #1-27

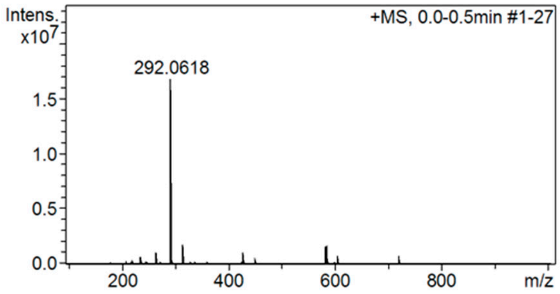

#	m/z	Res.	S/N	I	I %	FWHM
1	292.0618	38253	86879.0	16857124	100.0	0.0076

Figure S15 (d): HR-MS spectrum of 8-octen-6,10-diynyl-1,4-dioxocyclododeca[2,3-g]-5,8-quinolinedione (**27**).

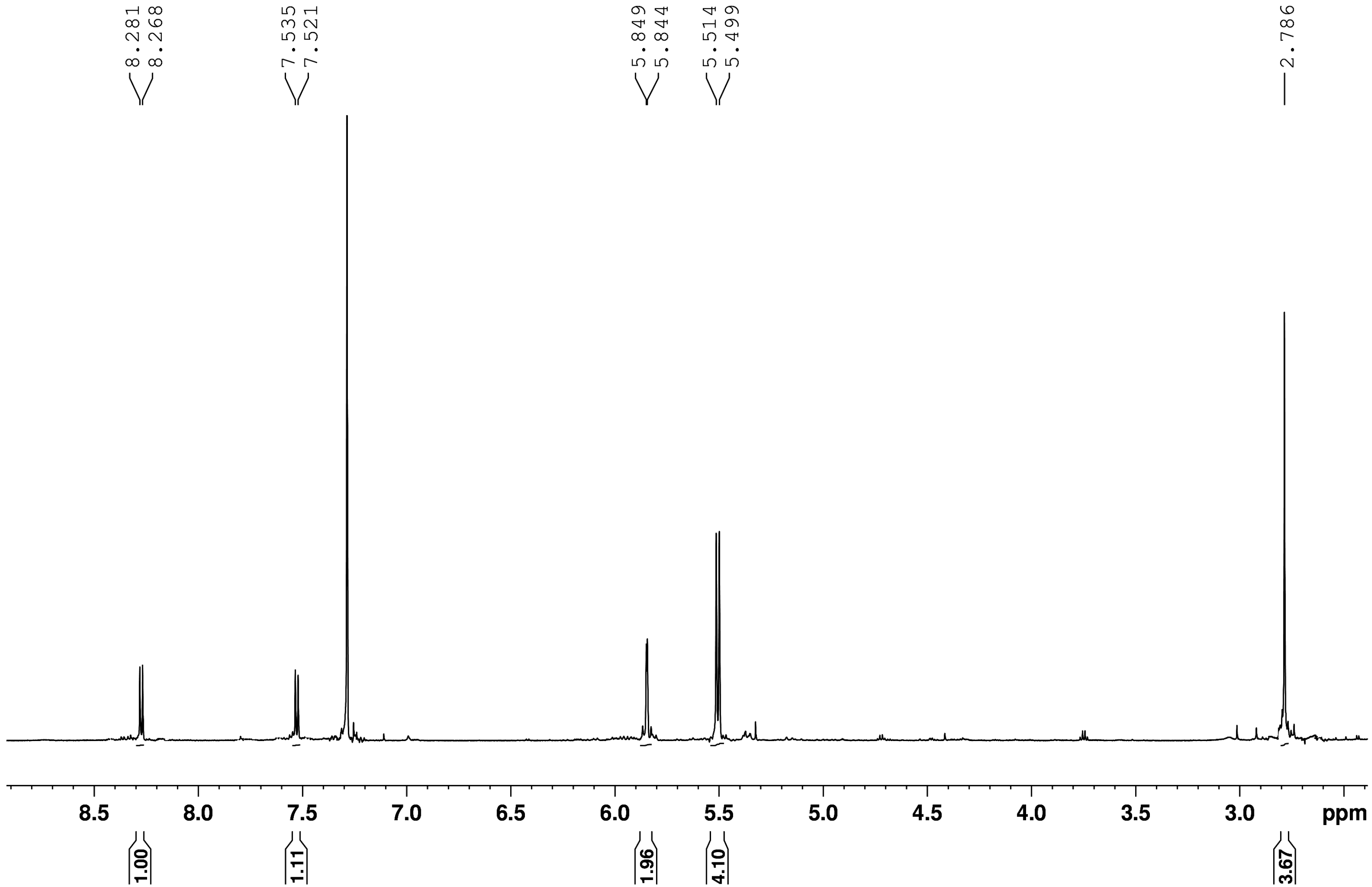

Figure S16 (a): ^{1}H-NMR spectrum of 8-octen-6,10-diynyl-1,4-8-octen-6,10-diynyl-1,4-dioxocyclododeca[2,3-g]-2-methyl-5,8-quinolinedione (**28**).

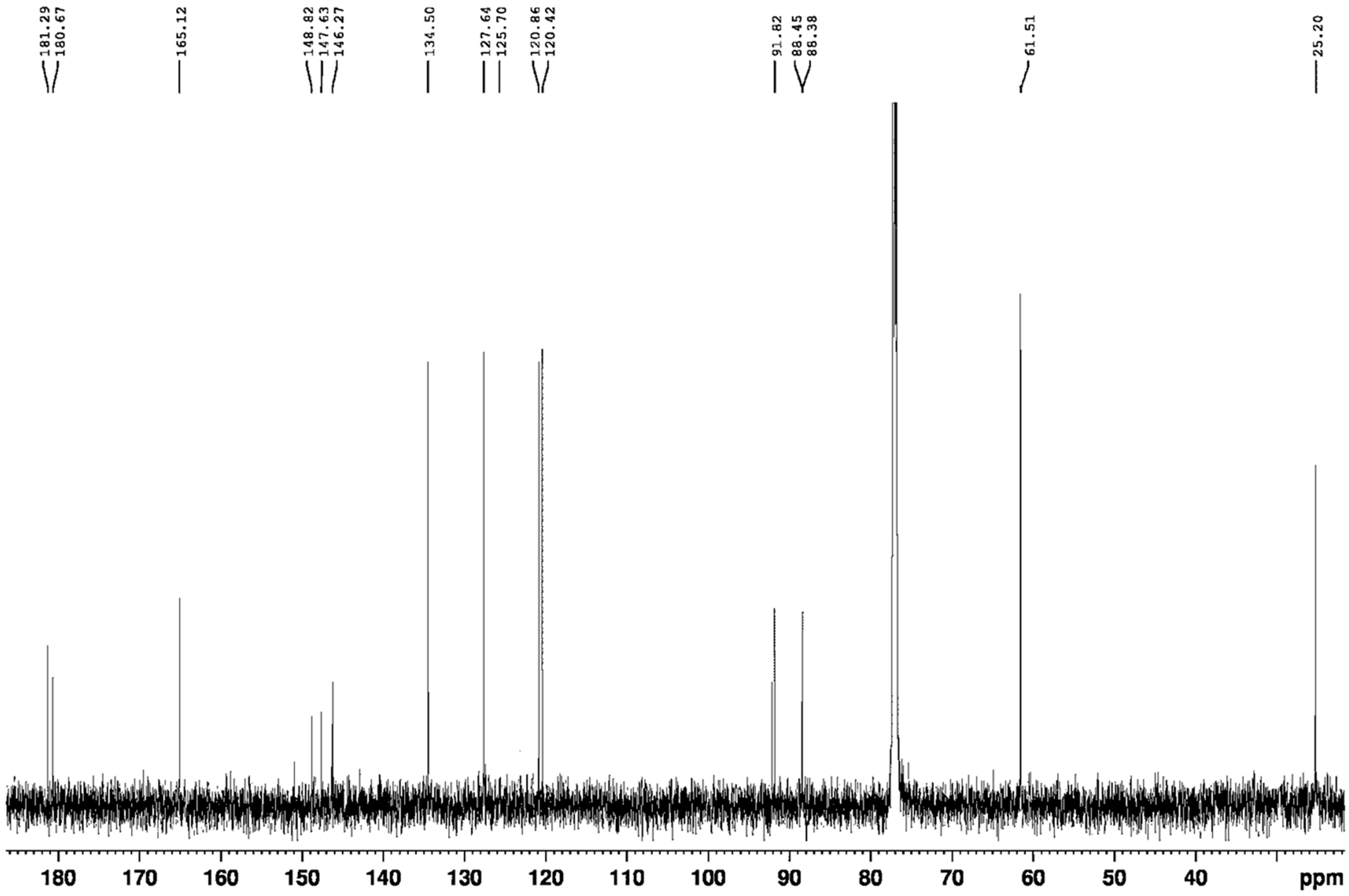

Figure S16 (b): ^{13}C-NMR spectrum of 8-octen-6,10-diynyl-1,4-8-octen-6,10-diynyl-1,4-dioxocyclododeca[2,3-g]-2-methyl-5,8-quinolinedione (**28**).

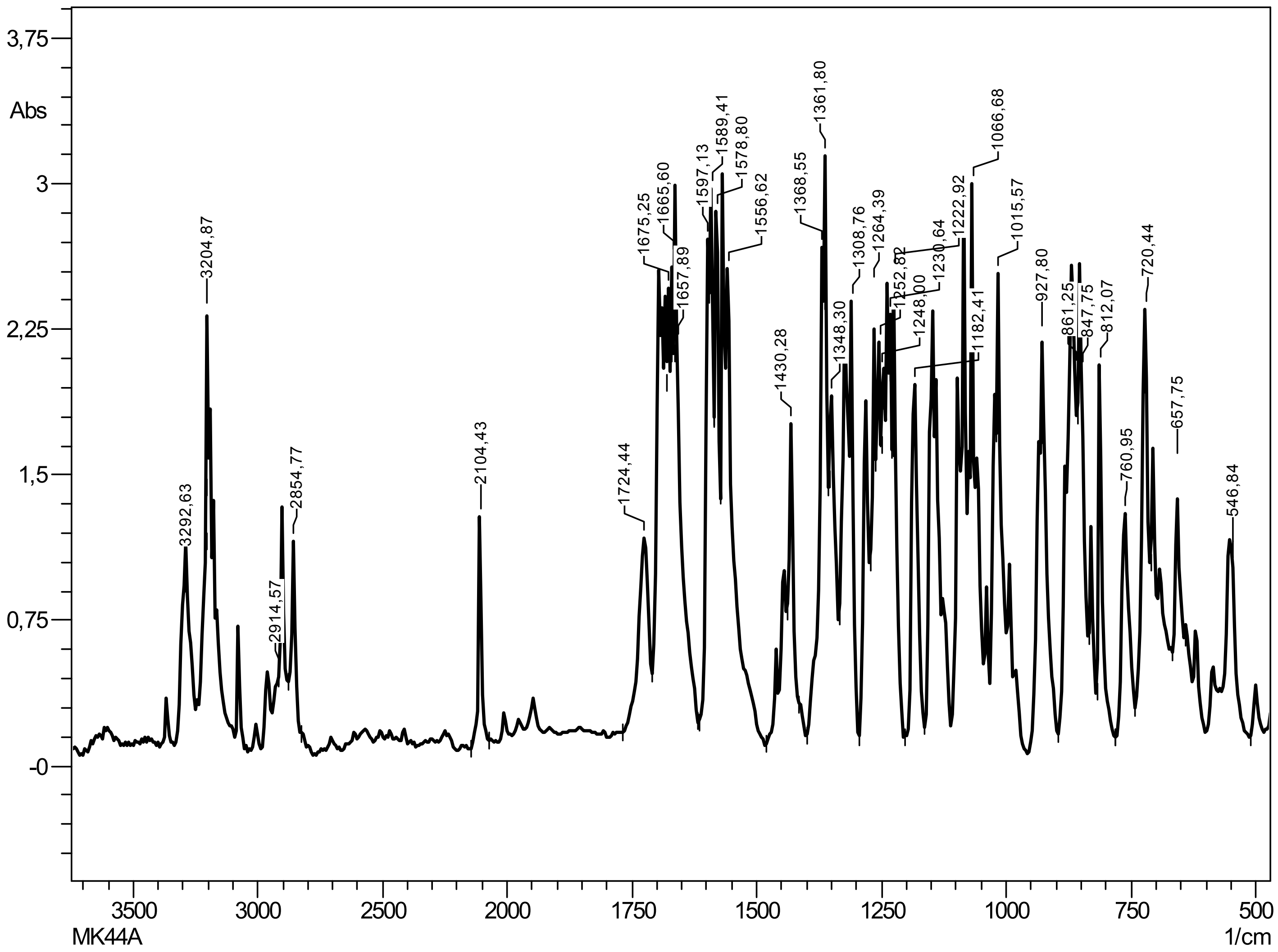

Figure S16 (c): IR spectrum of 8-octen-6,10-diynyl-1,4-8-octen-6,10-diynyl-1,4-dioxocyclododeca[2,3-g]-2-methyl-5,8-quinolinedione (**28**).

Compound Spectrum List Report

Analysis Info

Analysis Name	D:\Data\mk28.d	Acquisition Date	2/17/2017 12:08:53 PM	
Method	low_mass.m	Operator	KM	
Sample Name	TM Low concentration	Instrument	impact II	1825265.10082
Comment				

Acquisition Parameter

Source Type	ESI	Ion Polarity	Positive	Set Nebulizer	0.3 Bar
Focus	Active	Set Capillary	4000 V	Set Dry Heater	240 °C
Scan Begin	100 m/z	Set End Plate Offset	-500 V	Set Dry Gas	4.0 l/min
Scan End	1000 m/z	Set Charging Voltage	2000 V	Set Divert Valve	Source
		Set Corona	0 nA	Set APCI Heater	0 °C

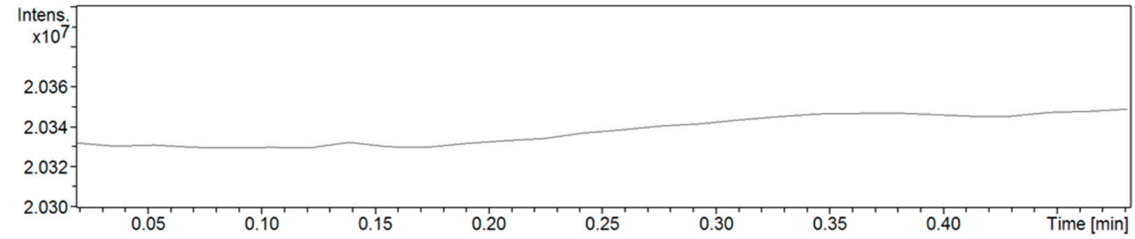

+MS, 0.0-0.4min #2-25

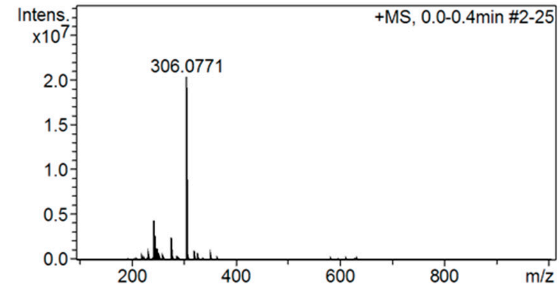

#	m/z	Res.	S/N	I	I %	FWHM
1	243.9932	36038	24252.1	4473754	22.0	0.0068
2	306.0771	25949	61360.4	20337186	100.0	0.0118

Figure S16 (d): HR-MS spectrum of 8-octen-6,10-diynyl-1,4-8-octen-6,10-diynyl-1,4-dioxocyclododeca[2,3-g]-2-methyl-5,8-quinolinedione (**28**).
